# Dopamine-mediated interactions between short- and long-term memory dynamics

**DOI:** 10.1038/s41586-024-07819-w

**Published:** 2024-07-22

**Authors:** Cheng Huang, Junjie Luo, Seung Je Woo, Lucas A. Roitman, Jizhou Li, Vincent A. Pieribone, Madhuvanthi Kannan, Ganesh Vasan, Mark J. Schnitzer

**Affiliations:** 1 James Clark Center, Stanford University, Stanford, CA USA; 2 Department of Biology, Stanford University, Stanford, CA USA; 3 Howard Hughes Medical Institute, Stanford University, Stanford, CA USA; 4 CNC Program, Stanford University, Stanford, CA USA; 5 The John B. Pierce Laboratory, New Haven, CT USA; 6 Department of Cellular & Molecular Physiology, Yale University, New Haven, CT USA; 7 Department of Applied Physics, Stanford University, Stanford, CA USA; 8 Present Address: Dept. of Neuroscience, Washington University School of Medicine, St. Louis, MO USA; 9 Present Address: Department of Electronic Engineering, The Chinese University of Hong Kong, Hong Kong, China; 10 Present Address: Department of Neuroscience, University of Minnesota, Minneapolis, MN USA

**Keywords:** Classical conditioning, Neural circuits, Learning algorithms, Long-term memory

## Abstract

In dynamic environments, animals make behavioural decisions on the basis of the innate valences of sensory cues and information learnt about these cues across multiple timescales^1–3^. However, it remains unclear how the innate valence of a sensory stimulus affects the acquisition of learnt valence information and subsequent memory dynamics. Here we show that in the *Drosophila* brain, interconnected short- and long-term memory units of the mushroom body jointly regulate memory through dopamine signals that encode innate and learnt sensory valences. By performing time-lapse in vivo voltage-imaging studies of neural spiking in more than 500 flies undergoing olfactory associative conditioning, we found that protocerebral posterior lateral 1 dopamine neurons (PPL1-DANs)^4^ heterogeneously and bidirectionally encode innate and learnt valences of punishment, reward and odour cues. During learning, these valence signals regulate memory storage and extinction in mushroom body output neurons (MBONs)^5^. During initial conditioning bouts, PPL1-γ1pedc and PPL1-γ2α′1 neurons control short-term memory formation, which weakens inhibitory feedback from MBON-γ1pedc>α/β to PPL1-α′2α2 and PPL1-α3. During further conditioning, this diminished feedback allows these two PPL1-DANs to encode the net innate plus learnt valence of the conditioned odour cue, which gates long-term memory formation. A computational model constrained by the fly connectome^6,7^ and our spiking data explains how dopamine signals mediate the circuit interactions between short- and long-term memory traces, yielding predictions that our experiments confirmed. Overall, the mushroom body achieves flexible learning through the integration of innate and learnt valences in parallel learning units sharing feedback interconnections. This hybrid physiological–anatomical mechanism may be a general means by which dopamine regulates memory dynamics in other species and brain structures, including the vertebrate basal ganglia.

## Abstract (web summary)

In the brain of fruit flies, the mushroom body achieves flexible learning by using interconnected short- and long-term memory units for dopamine-mediated integration of innate valences with learnt valences obtained through experience.

## Main

When navigating changing environments, animals evaluate the innate and learnt valences of sensory cues. The former represent predictions that may promote survival, such as those about threats or food, whereas the latter represent updates to these predictions based on experience^2^. Many species process innate and learnt valences in distinct neural pathways, which may promote behavioural reliability and flexibility^1,3,8^. Whether innate valences shape the acquisition of learnt valence information, and what functional benefits such interactions might confer, have remained unknown.

One possibility is that innate valences modulate learning through dopamine teaching signals that convey both innate and learnt information. Mammalian dopamine neurons (DANs) encode reward predictions and prediction errors, as well as motivational values^9^ and novelties or identities of unfamiliar cues, showing that DANs can signal certain innate facets of sensory cues^10^. In *Drosophila*, DANs also process innate and learnt valences. The PPL1 and protocerebral anterior medial (PAM) clusters of DANs provide the fly mushroom body with negative and positive reinforcement signals, respectively, that drive synaptic plasticity and learning^5,11^. Notably, co-activation of DANs and odour-responsive, mushroom body Kenyon cells induce olfactory learning^12,13^. But DANs also innately respond to odorants, not just to aversive or rewarding stimuli^14^.

**Fig. 1:**
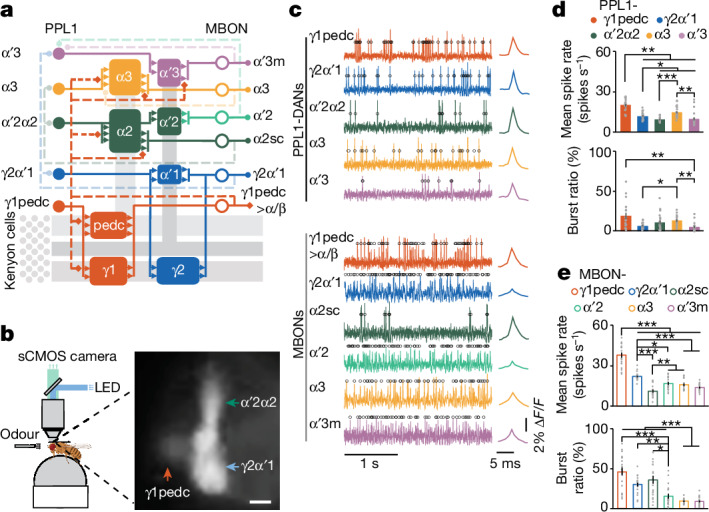
Voltage imaging of PPL1-DANs and MBONs. **a**, PPL1-DAN and MBON connectivity. Five PPL1-DANs innervate eight compartments of the mushroom body and modulate six downstream MBONs. Kenyon cells and their axons are shown in grey. Solid and dashed lines indicate feedforward and feedback connections, respectively. **b**, Left, the voltage-imaging set-up. Flies could walk or run on a trackball, which recorded their locomotor responses to odour presentations. Fluorescence voltage imaging of neural activity was performed using an sCMOS camera. Created with BioRender.com. Right, fluorescence image of pAce voltage-indicator expression in PPL1-γ1pedc, -γ2α′1 and -α′2α2 (fly line *MB504B-GAL4*). Scale bar, 10 μm. **c**, Left, optical voltage traces showing spontaneous spikes in PPL1-DANs and MBONs. Black circles indicate identified spikes. Right, mean optical spike waveforms. Δ*F/F* indicates the change in relative fluorescence intensity. **d**,**e**, Spike rates (top) and spontaneous burst ratios (bottom) from PPL1-DANs (**d**) and MBONs (**e**). Grey dots denote data from 20 individual flies per cell type. *P < 0.05, **P < 0.01, ***P < 0.001; Kruskal–Wallis analysis of variance (ANOVA) and post-hoc *U*-tests with Holm–Bonferroni correction. Data in **d**,**e** are mean ± s.e.m. Source Data

The PPL1-DANs and MBONs interconnect in a parallel-recurrent circuit of multiple learning units sharing widespread feedback connections^5,6^ (Fig. 1a). Multiple memory traces can exist concurrently across different units; in each unit, one DAN controls synaptic plasticity. DANs also receive recurrent signals from MBONs conveying learnt valence information^6,7,15^. We hypothesized that DANs integrate innate valence signals coming from the sensory system (through the mushroom body) and learnt valences stored in the memory (by MBONs).

We tested this hypothesis through long-term optical voltage-imaging studies in more than 500 flies. Unlike Ca^2+^ imaging, voltage imaging reports neural spikes with millisecond resolution, revealing spiking excitation and suppression^16^. We studied how PPL1-DAN teaching signals encode and integrate innate and learnt valences, and found that this integration allows short-term memory to regulate long-term memory formation through MBON → DAN feedback, enabling complex interactions between short- and long-term memory.

## Long-term voltage imaging

**Extended Data Fig. 1:**
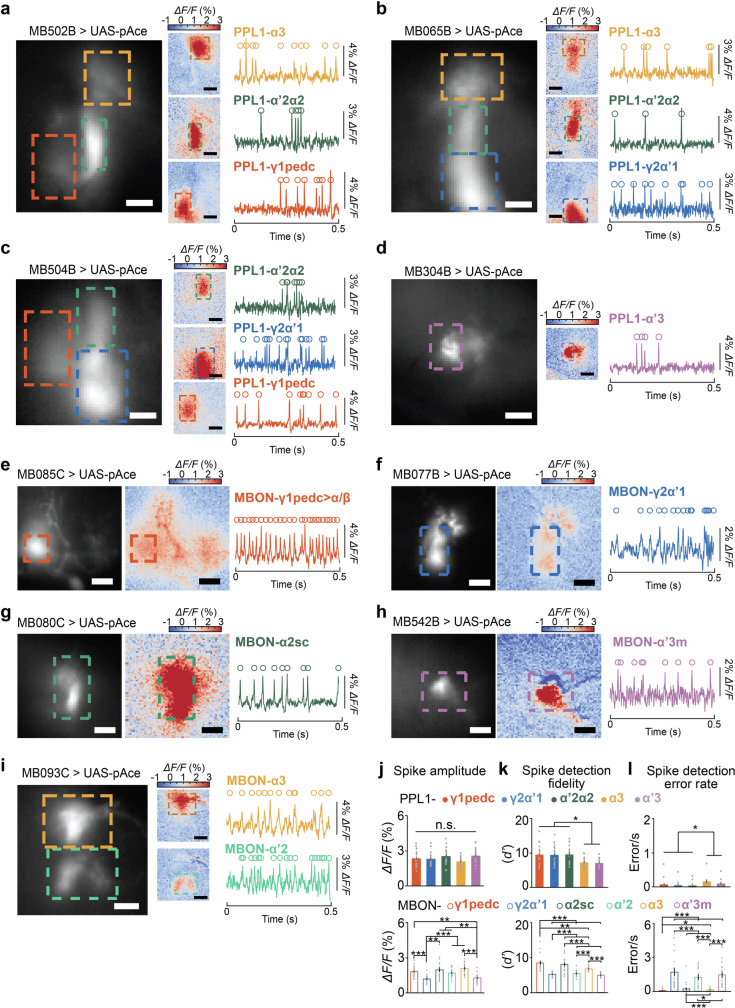
Voltage imaging of neural spiking activity using split-GAL4 lines to label PPL1-DANs and their downstream MBONs. **a**–**i**) Example data from voltage imaging studies of 5 different types of PPL1-DANs and 6 different types of MBONs. *Left panels*, Mean fluorescence images (averaged over 15 s of voltage movies), showing the patterns of pAce expression in 9 different split-GAL4 lines. Dashed boxes demarcate regions-of-interest (ROIs) from which we aggregated fluorescence signals to determine fluorescence traces of transmembrane voltage activity in individual neurons. *Middle panels*, Spatial maps of the mean fluorescence responses (Δ*F*/*F*) of individual neuron-types at the peaks of their action potentials, averaged over all spikes detected within the selected 15-s-intervals. Dashed boxes enclose the same regions as in the left panels. *Right panels*, Traces of each neuron’s relative fluorescence changes (Δ*F*/*F*) during the same 15-s-intervals used to create the spatial maps. Open circles mark detected spikes. All scale bars are 10 μm. **j**–**l**) Mean ± s.e.m. values of the spike amplitude, **j**, spike detection fidelity (*d*′), **k**, and spike detection error rate, **l**, for PPL1-DANs (*top graphs*) and MBONs (*bottom graphs*). Gray dots are data points for 20 individual flies for each neuron-type. Spike-detection fidelity values, *d*′, and error rates were satisfactory in all neuron-types (DAN error rates: 0.05–0.16 s^−1^; MBON error rates: 0.06–1.7 s − 1). MBON-γ2α’1, MBON-α’2, and MBON-α’3 m had the lowest *d*′ values and highest spike detection error rates, likely reflecting their smaller spike amplitudes, as seen in panel **j** and as reported previously for MBON-γ2α’1^12^. All 4 graphs exhibited significant differences across neuron-types (*n* = 20 flies per neuron-type; Kruskal-Wallis ANOVA). Horizontal lines and asterisks mark pairwise comparisons that yielded significant differences in post-hoc Mann-Whitney U-tests performed with a Holm-Bonferroni correction for multiple comparisons (*P < 0.05 and ***P < 0.001). Source Data

To image spiking across an olfactory conditioning assay (Fig. 1b and Supplementary Video 1), we used a laser microsurgical preparation^17^ for long-term imaging^18^ and predominantly the positive-polarity voltage indicator, pAce^16^, a FRET–opsin indicator we created based on our earlier negative-polarity indicator using an opsin from the algae *Acetabularia*^19^. Using Split-GAL4 fly lines^5^, we expressed pAce in five PPL1-DAN (PPL1-γ1pedc, -γ2α′1, -α′2α2, -α3 and -α′3m) and six MBON (MBON-γ1pedc>α/β, -γ2α′1, -α2sc, -α′2, -α3 and -α′3) subtypes (Extended Data Fig. 1a–i and Extended Data Table 1).

**Extended Data Fig. 2:**
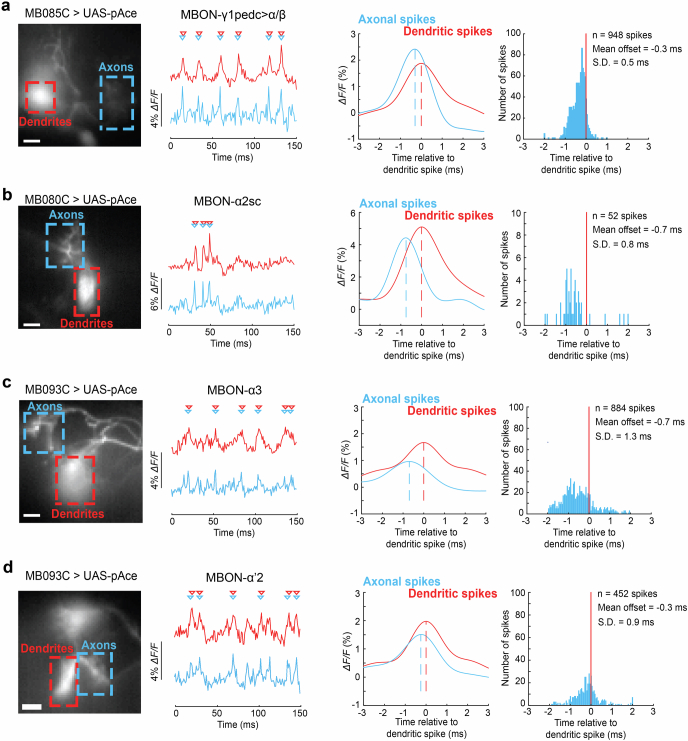
Axonally initiated spikes in MBONs backpropagate into the dendritic arbors. **a**–**d**) Example data from 4 different types of MBONs showing the backpropagation of spikes into the dendritic tree. *Left panels*, Mean fluorescence images (each an average over a 15-s-interval of a voltage movie), showing the spatial pattern of pAce expression in an individual MBON-type. Dashed boxes demarcate regions-of-interest (ROIs) that enclose either axons (*blue*) or dendrites (*red*) of the selected cell. Scale bars are 10 μm. *Left middle panels*, Fluorescence traces of transmembrane voltage activity from each cell’s axonal ROI (blue traces) and dendritic ROI (red traces). Triangles mark the peak of detected action potentials. *Right middle panels*, Mean waveforms of the axonal (blue traces) and dendritic spikes (red traces), computed by spline interpolation of the fluorescence traces determined from the 1-kHz-voltage imaging data. Vertical dashed lines mark the peaks of these spike waveforms and reveal the temporal offsets between axonal and dendritic spikes. *Right panels*, Histograms of the temporal offsets between axonal and dendritic spikes, assessed relative to the time of the dendritic spike. Source Data

Using 1-kHz imaging of mushroom body compartments innervated by PPL1-DAN axons and MBON dendrites, we found variable spontaneous spiking and bursting rates across all 11 neuron types (Fig. 1c–e and Extended Data Fig. 1a–i). Spike-detection fidelity values, *d*′, and error rates were satisfactory in all neuron types (Extended Data Fig. 1j–l). In MBONs, spikes back-propagated from axonal regions into dendrites (Extended Data Fig. 2 and Supplementary Video 2), which might facilitate spike-timing-dependent plasticity^20^.

## PPL1-DANs encode innate valences heterogeneously and bidirectionally

**Fig. 2:**
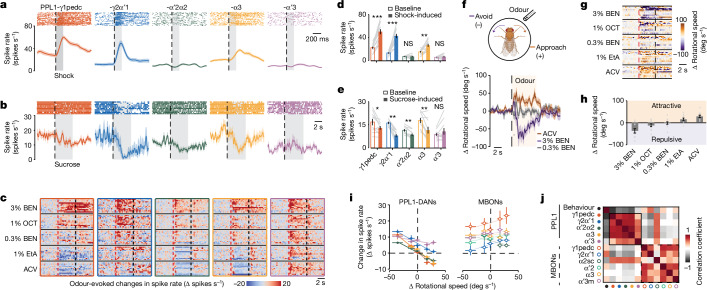
PPL1-DANs heterogeneously and bidirectionally encode punishment, reward and odour valence. **a**,**b**, Top, raster plots of PPL1-DAN spiking on trials when flies received either an individual 200-ms electric-shock pulse (**a**; 36 trials; 12 flies per cell type, 3 trials per fly, spaced 1.8 s apart) or sucrose for 5 s (**b**; 10 flies per cell-type, 1 trial per fly). Dashed lines indicate the onset of shock or sucrose availability; grey shading shows periods of shock or feeding. Bottom, trial-averaged mean spike rates (colour shading, s.e.m.). **c**, Odour-evoked changes in PPL1-DAN spiking relative to the rate in the 5 s before odour delivery (12 flies, 1 trial per odour per fly). Red and black dashed lines indicate odour onset and offset, respectively. Extended Data Fig. 4 has analogous MBON data. BEN, benzaldehyde in mineral oil; OCT, 3-octanol in mineral oil; EtA, ethyl acetate in mineral oil; ACV, apple cider vinegar. **d**,**e**, PPL1-DAN spike rates before (baseline, unfilled bars) and during (filled bars) the shock (**d**; 12 flies per cell type) or sucrose (**e**; 10 flies per cell type). *P < 0.05; **P < 0.01; ***P < 0.001; NS, not significant; signed-rank test. **f**, Top, odour-evoked behavioural responses of flies were measured on a trackball. Created with BioRender.com. Bottom, mean changes in rotational speed of wild-type flies (*w*^*1118*^) in response to a 5-s presentation (shaded interval) of ACV or either 0.3% or 3% BEN. Shading on time traces denotes s.e.m. over 36 total trials in 12 flies. **g**, Odour-evoked rotational responses. Each row shows one fly’s change in rotational speed, averaged over 3 trials per odour for 5 different odours (*n* = 12 flies). Red and black dashed lines indicate odour onset and offset, respectively. **h**,Evoked changes in rotational speed, averaged over 5-s odour presentations and 36 trials per odour (12 flies, 3 trials per fly), plotted left to right from the most repulsive to the most attractive odour. Grey dots represent data from individual flies. **i**, Odour-evoked changes in PPL1-DAN and MBON spike rates plotted against changes in fly turning speed induced by the same odorants. Coloured lines show linear regressions (12 flies per cell type). Source data has *R-*values and *P-*values for the regressions. **j**, A 12 × 12 matrix of correlation coefficients, computed for the 11 neuron types using their mean responses to the 5 odorants in **i**, and the flies’ mean rotational responses to each odour in **h** (12 flies per cell type). Data in **d**,**e**,**h**,**i** are mean ± s.e.m. Source Data

To assess valence coding, we tracked PPL1-DAN responses to punishments (electric shock), rewards (sucrose) and odours. After the onset of shock pulses to the thorax, spiking increased in PPL1-γ1pedc, -γ2α′1 and -α3 neurons, and decreased after shock offset, whereas sucrose-intake decreased spiking in PPL1-γ1pedc, -γ2α′1, -α′2α2 and -α3 neurons (Fig. 2a–e).

**Extended Data Fig. 3:**
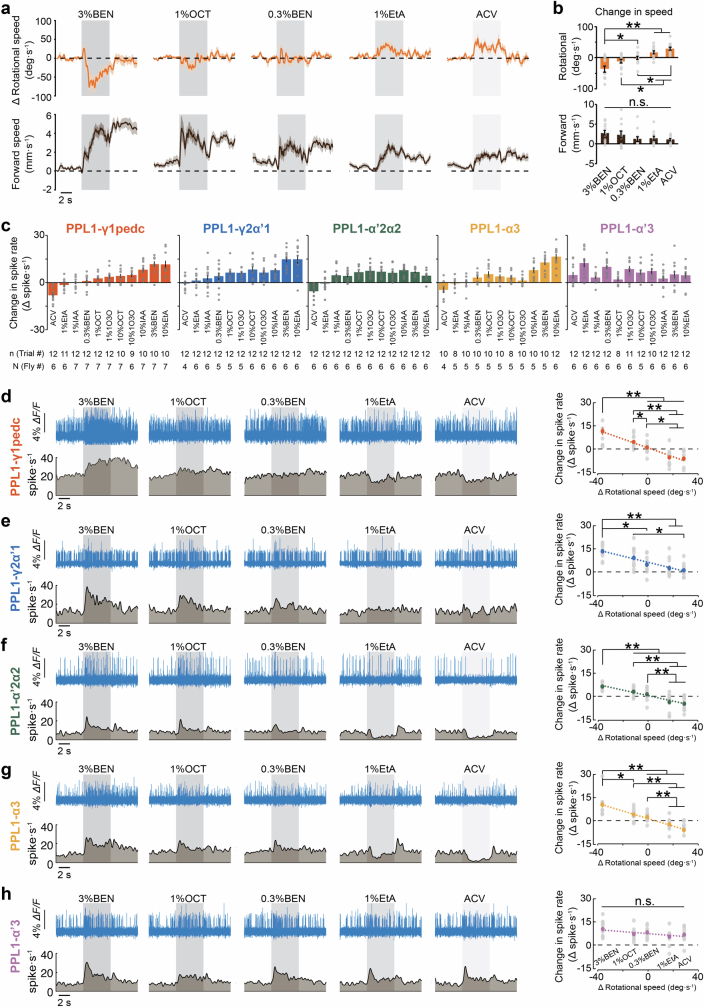
PPL1-DANs bidirectionally encode innate odour valences. **a**) Mean time-dependent forward walking speeds (*lower panels*) and changes in rotational speed (*upper panels*) of wild-type flies (*w*^*1118*^) in response to 5 different odours (3% BEN, 1% OCT, 0.3% BEN, 1% EtA, and ACV), each presented for a duration of 5 s. Gray shading marks the duration of odour delivery. Shading on the time traces: s.e.m. over 36 total trials in 12 flies. **b**) Mean ± s.e.m. changes in the rotational (*upper panel*) and forward walking speeds (*lower panel*) of wild-type flies (*w*^*1118*^) relative to baseline values in the 5 s before odour presentation, in response to the 5 odours used in **a**, averaged over the 5 s of odour presentation and 36 trials per odour (*n* = 12 flies, 3 trials per fly). Individual points denote data from individual flies. (*P < 0.05 and **P < 0.01; *n* = 12 flies per neuron type; Friedman ANOVA followed by post-hoc Wilcoxon signed-rank tests with Holm-Bonferroni correction). **c**) Mean ± s.e.m. changes in spike rates measured during odour exposure (5 s duration), relative to baseline spiking rates, in PPL1-γ1pedc, -γ2α’1, -α’2α2, -α3 and -α’3 neurons (*n* = 4–7 flies per odor, 1–4 trials per fly; the exact total numbers of trials and flies used are indicated under each odour-type in the graphs). Odorants: Apple cider vinegar (ACV), 0.3% or 3% benzaldehyde in mineral oil (0.3% or 3% BEN), 1% or 10% 3-octanol in mineral oil (1% or 10% OCT), 1% or 10% ethyl acetate in mineral oil (1% or 10% EtA), 1% or 10% 1-octen-3-ol in mineral oil (1% or 10% 1O3O). The different PPL1-DANs exhibit different degrees of sensitivity to odour valence. Odours are arranged left to right in the plots according to their evoked spiking changes in PPL1-γ1pedc. **d**–**h**) *Upper left panels*, Example optical voltage traces showing PPL1-DAN responses to presentations of the same 5 odours as in **b**. Gray shading covers the 5-s-duration of odour delivery. *Lower left panels*, Mean time-dependent spike rates in response to each odor, averaged over 12 flies for each neuron-type, 1 trial per odor. *Right panels*, Plots of the mean changes in PPL1-DAN spike rates evoked by odour exposure (5 s exposure duration; the 5 odorants used are listed above the *x*-axis in **h**), plotted as a function of the mean changes in rotational speed induced by each odorant (using the data of Fig. 2h). Gray points show data from 12 individual flies per neuron-type. As in Fig. 2h, odour data are plotted from left to right on the *x*-axis from the most repulsive to the most attractive odor. Dashed lines are linear regressions (Source data has *R-*values and *P-*values for the regressions). Although individual odours induced characteristic spiking patterns in PPL1-DANs, distinct odours of similar valences were not readily distinguished based solely on changes in spike rates, suggesting that the spike rates encode odours’ innate valences rather than their actual identities. Horizontal lines and asterisks mark pairwise comparisons that yielded significant differences in post-hoc Wilcoxon signed-rank tests performed with a Holm-Bonferroni correction for multiple comparisons (*P < 0.05 and **P < 0.01), following a Friedman ANOVA. See Extended Data Fig. 4g for data on odour-evoked spiking in MBONs. Source Data

We next tested behavioural odour preferences without voltage imaging. We delivered odours from a fly’s front left or right side and recorded its locomotor responses on a trackball towards or away from the odour (Fig. 2f). We found five odours that evoked responses from vigorous approach to vigorous avoidance, indicating wide-ranging odour valences (Fig. 2f–h and Extended Data Fig. 3a,b).

**Extended Data Fig. 4:**
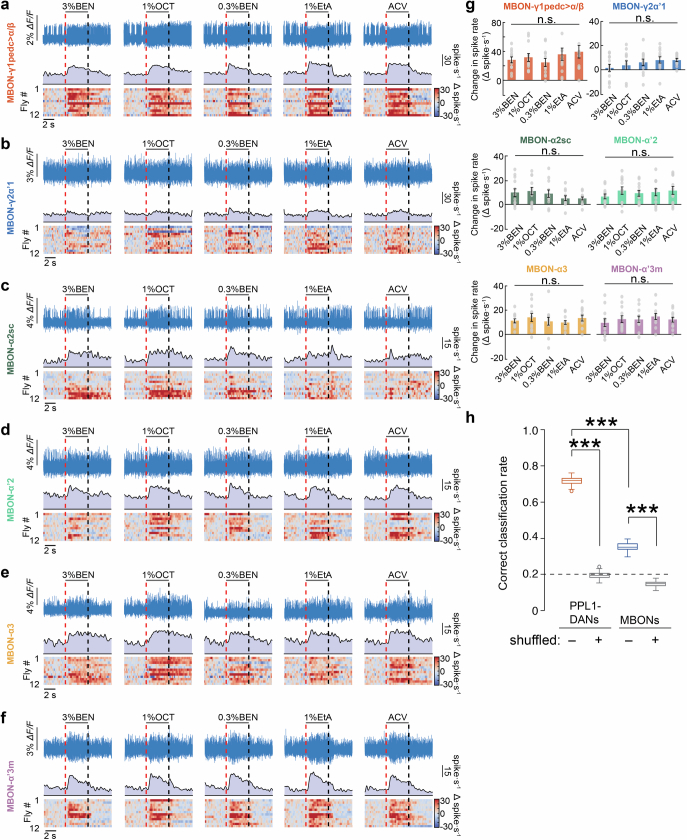
Odour-evoked responses of MBONs do not vary much across odours with different innate valences. **a**–**f**) Example fluorescence voltage traces (*top rows*), time-dependent mean spiking rates (*middle rows*), and odour-evoked changes in 12 individual flies’ spike rates relative to baseline rates (*bottom rows*), for 6 different MBON-types during 5-s-presentations of 5 different odours. Red vertical dashed lines mark the onsets of odour presentation, and black vertical dashed lines mark the offsets. The mean spike rates shown in the middle row of each graph are averages over the same 12 flies, for which data is shown individually in the corresponding graph of the bottom row. **g**) Mean ± s.e.m. changes in spike rates measured for 6 different MBON-types during 5-s-odour presentations, determined relative to baseline spiking rates. Gray dots: data from individual flies. None of the bar graphs exhibited significant differences in the MBON responses to different odorants (Friedman ANOVA; *P* > 0.05). **h**) To estimate the extent to which a fly might be able to distinguish the different odorants used in our study based solely on the odour-evoked patterns of neural activity across the 5 PPL1-DANs or 6 MBONs, we performed a classification analysis using a collection of neural recordings from 117 total flies, in which the dynamics of each neuron-type was recorded in 12 different flies (Some fly lines allowed recordings from more than 1 cell-type at once; Methods; Extended Data Table 1). The box-and-whisker plot shows the accuracy of odour classification using the set of mean odour-evoked changes in neural activity across either the 5 PPL1-DANs or the 6 MBONs (n = 120 sub-testing sets used for each; see Methods). Notwithstanding that there are fewer PPL1-DANs than MBONs, classification was significantly more accurate based on PPL1-DAN activity patterns. We compared the results to those attained using shuffled datasets, in which odour identities were randomly permuted. Boxes span the 25th–75th percentiles, horizontal lines denote median values, whiskers span 1.5 times the interquartile distance, and open circles are outlier data points. Gray dashed line indicates chance level (***P < 0.001; Wilcoxon ranked sum test). Source Data

Using these five odours, we imaged PPL1-DAN and MBON responses. Unlike the odour-evoked excitations observed in Ca^2+^-imaging studies^13,14,21^, we found that odours bidirectionally modulated PPL1-DAN spiking, according to flies’ odour-evoked behavioural responses (Fig. 2c,i; Extended Data Fig. 3c–h). An exception was PPL1-α′3, which mainly exhibited excitations. MBONs had excitatory responses with amplitudes unrelated to odour valence^22^ (Extended Data Fig. 4a–g).

Across the five odours, the responses of DANs and MBONs correlated positively with those of other DANs and MBONs, respectively, but negatively across the two cell classes (Fig. 2i,j). MBON-α2sc was an exception, with odour responses resembling those of PPL1-DANs. PPL1-DAN odour responses correlated well with odour-evoked behavioural responses, apart from PPL1-α′3. The MBON odour responses were more variable across flies and correlated more weakly with behaviour. Thus, PPL1-DAN responses enabled more-accurate classifications of odour valences (Extended Data Fig. 4h), suggesting that DAN odour coding is not merely inherited through feedback from MBONs but instead reflects diverse inputs^6^.

## Learning induces bidirectional plasticity across PPL1-DANs and MBONs

**Fig. 3:**
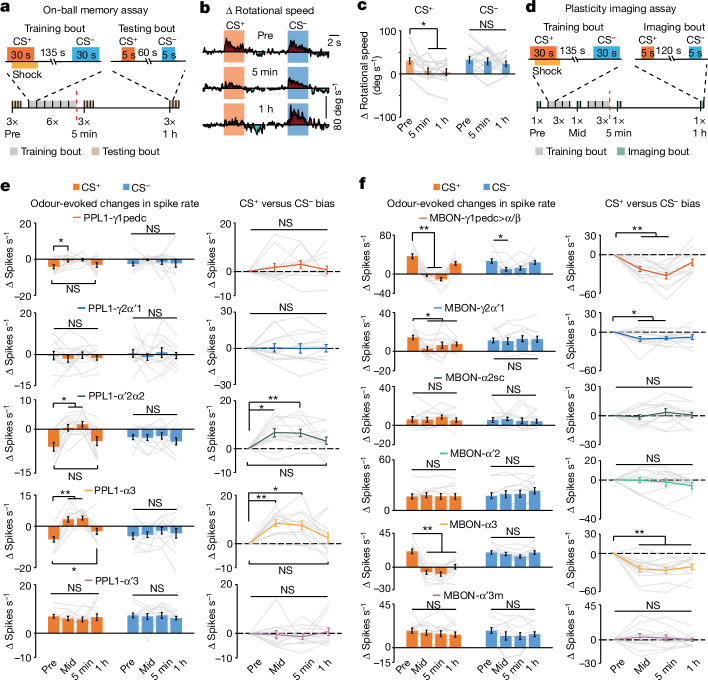
Learning induces distributed bidirectional plasticity in PPL1-DANs and MBONs. **a**, On-ball memory assay. Bottom, timeline of associative learning and memory assay for flies on a trackball. Each fly had three bouts of odour testing before conditioning (Pre), characterizing its initial responses to the CS^+^ and CS^−^ odours. Next, there were six training bouts, each with a paired presentation of the CS^+^ and the unconditioned stimulus (US, electric shock pulses) plus an unpaired CS^−^ presentation. Finally, each fly had three testing bouts at both 5 min and 1 h after conditioning. Red line indicates the end of training. Top, timelines for individual training and testing bouts. A pair of innately attractive odours (ACV and 1% ethyl acetate) were counterbalanced as CS^+^ and CS^−^ across flies for **b**–**f**. **b**, Mean time-dependent behavioural responses on the trackball to the CS^+^ and CS^−^ in odour-testing bouts before training (top) and 5 min (middle) and 1 h (bottom) after conditioning. Each trace is an average over 12 flies and the 3 test bouts in each period. **c**, Rotational speed changes induced by CS^+^ and CS^−^ odours before and 5 min and 1 h after training sessions (*n* = 12 flies). **d**, Bottom, timeline for voltage-imaging assay of learning and memory. Each fly had one imaging bout before conditioning, characterizing spiking responses to the two innately attractive odours. Next, there were three training bouts, each with a paired CS^+^ and electric-shock US, plus an unpaired CS^−^. Then, 5 min after the third training bout, we imaged odour-evoked spiking (mid-training, Mid). Next, 5 min after this mid-training imaging bout, there were three more training bouts. There were additional imaging bouts 5 min and 1 h after conditioning. The red dashed line indicates the end of training. Top, timelines for individual training and imaging bouts. **e**,**f**, Left, changes in spike rates of PPL1-DANs (**e**), or MBONs (**f**), induced by CS^+^ and CS^−^ odours in the four different imaging periods. Right, CS^+^ versus CS^−^ bias in evoked responses, relative to pre-training responses (Methods); *n* = 12 flies per cell-type. In **c**,**e**,**f**, data are mean ± s.e.m. and grey lines show data from individual flies. *P < 0.05, **P < 0.01; Friedman ANOVA and post-hoc signed-rank tests with Holm–Bonferroni correction. Source Data

To probe mushroom body coding and plasticity during learning, we developed an associative conditioning assay for head-fixed flies behaving on a trackball or undergoing voltage imaging (Fig. 3a–d). As with conventional T-maze conditioning assays, ours had six training bouts, each with sequential exposures to a pair of conditioned-stimulus (CS^+^ and CS^−^) odours of the same initial valence. In each bout we paired a 30-s CS^+^ delivery with a 30-s pulsed electric shock to the fly. After conditioning with innately attractive odours, flies reduced their approaches to the CS^+^ but not to the CS^−^ for 1 h or more (Fig. 3b,c).

**Extended Data Fig. 5:**
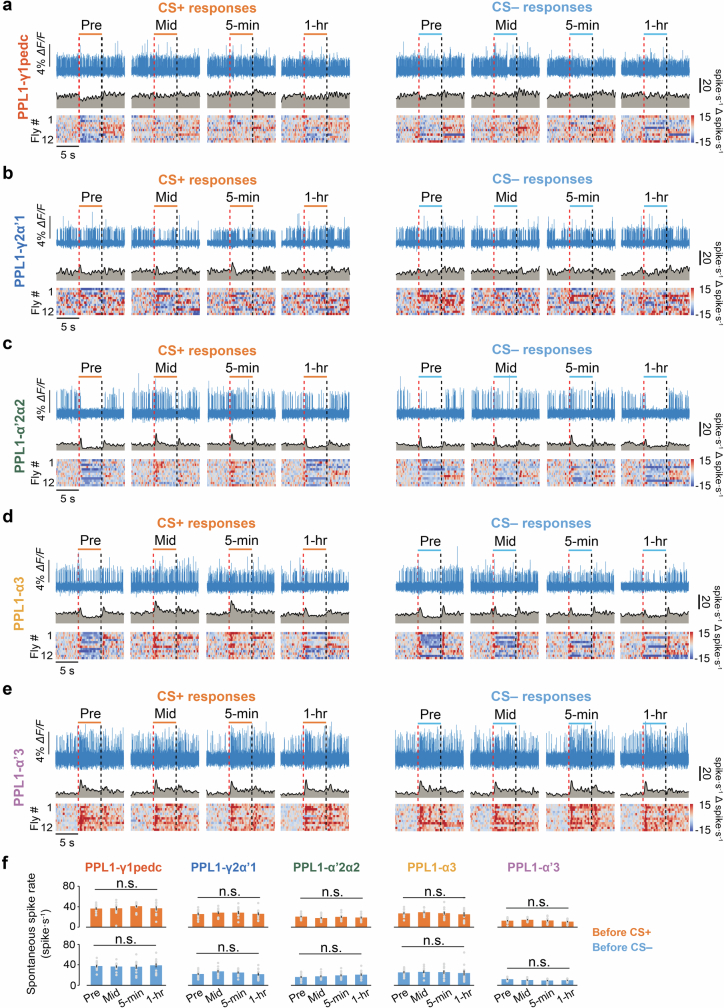
Associative conditioning with an attractive odour pair increases CS^+^-evoked responses in PPL1-α’2α2 and PPL1-α3 but not PPL1-γ1pedc, PPL1-γ2α’1 and PPL1-α’3. **a**–**e**) Example fluorescence voltage traces (*top rows*), time-dependent mean spiking rates (*middle rows*), and odour-evoked changes in spike rates relative to baseline rates in 12 individual flies (*bottom rows*) for PPL1-γ1pedc (**a**), PPL1-γ2α’1 (**b**), PPL1-α’2α2 (**c**), PPL1-α3 (**d**), and PPL1-α’3 (**e**) neurons in response to innately attractive CS^+^ and CS^–^ odours in the pre-training (Pre), mid-training (Mid), 5-min post-training, and 1-hr post-training periods (as defined in Fig. 3d). Red vertical dashed lines mark the onsets of odour presentation, and black vertical dashed lines mark the offsets. The mean spike rates shown in each graph of the middle rows are averages over the same 12 flies, for which data is shown individually in the corresponding graph of the bottom row. For each conditioning experiment, we used apple cider vinegar (ACV) and 1% ethyl acetate and assigned them as CS^+^ and CS^–^ in a counterbalanced manner across 12 flies. **f**) Mean ± s.e.m. baseline spike rates, averaged over the 5-s period before CS^+^ or CS^–^ delivery, in the pre-training (Pre), mid-training (Mid), 5-min post-training, and 1-hr post-training periods for the PPL1-γ1pedc, PPL1-γ2α’1, PPL1-α’2α2, PPL1-α3, and PPL1-α’3 neurons. Gray dots: data from individual flies. None of the bar graphs evidenced significant differences between the different time points (Friedman ANOVA; *P* > 0.05; *n* = 12 female flies per cell-type). Source Data

Voltage-imaging experiments showed that CS^−^-evoked responses in all PPL1-DANs were unaltered by conditioning (Fig. 3e and Extended Data Fig. 5). CS^+^-evoked responses of PPL1-α′2α2 and -α3 evolved across 3–6 training bouts, changing from odour-evoked decreases in spiking to evoked increases and then back to evoked decreases after 1 h, suggesting that learnt valence information transiently alters valence coding in these cells. PPL1-γ1pedc consistently exhibited CS^+^-evoked spiking decreases, but with diminished amplitudes at 5 min, but not 1 h, after conditioning. PPL1-γ2α′1 and -α′3 neurons were unaffected by conditioning, as was spontaneous DAN spiking. After training, the differential changes in evoked spike rates, which we quantified using a CS^+^ versus CS^–^ bias (Methods), were biased to the CS^+^ in PPL1-α′2α2 and -α3 but not in other PPL1-DANs.

**Extended Data Fig. 6:**
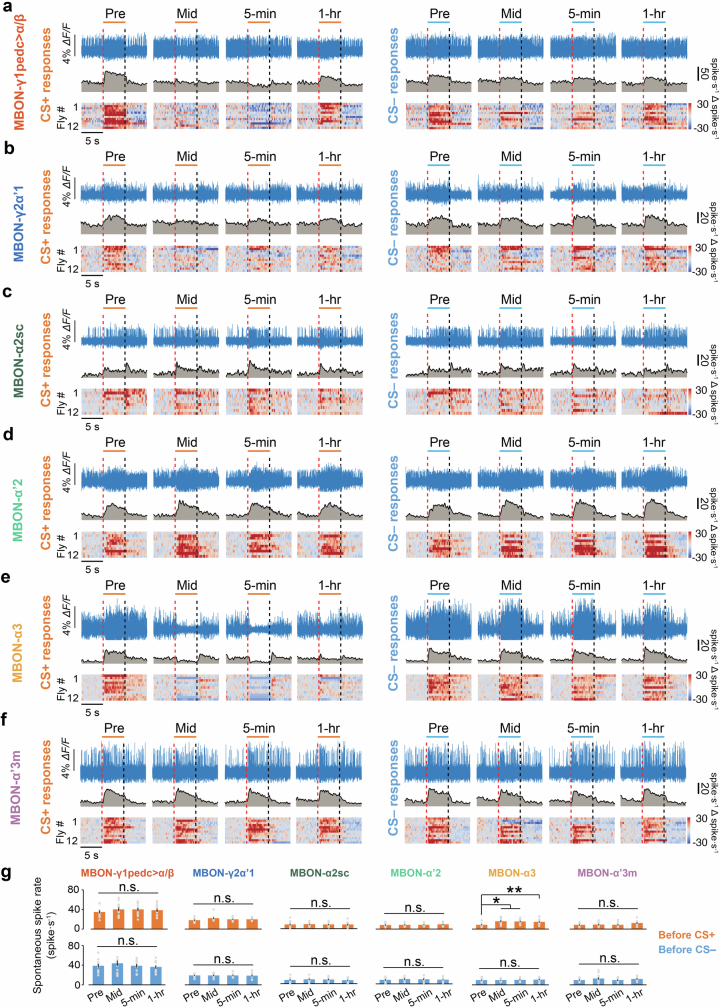
Olfactory conditioning with an attractive odour pair decreases CS^+^ responses in MBON-γ1pedc > α/β, MBON-γ2α’1 and MBON-α3. **a**–**f**) Example fluorescence voltage traces (*top rows*), time-dependent mean spiking rates (*middle rows*), and odour-evoked changes in spike rates relative to baseline rates in 12 individual flies (*bottom rows*) for MBON-γ1pedc > α/β (**a**), MBON-γ2α’1 (**b**), MBON-α2sc (**c**), MBON-α’2 (**d**), MBON-α3 (**e**) and MBON-α’3 m (**f**) in response to CS^+^ and CS^–^ odours in the pre-training (Pre), mid-training (Mid), 5-min post-training, and 1-hr post-training periods (as defined in Fig. 3d). Red vertical dashed lines mark the onsets of odour presentation, and black vertical dashed lines mark the offsets. The mean spike rates shown in each graph of the middle rows are averages over the same 12 flies, for which data is shown individually in the corresponding graph of the bottom row. For each conditioning experiment, we used apple cider vinegar (ACV) and 1% ethyl acetate and assigned them as CS^+^ and CS^–^ in a counterbalanced manner across 12 flies. **g**) Mean ± s.e.m. baseline spike rates, averaged over the 5-s period before CS^+^ or CS^–^ delivery, in the pre-training (Pre), mid-training (Mid), 5-min post-training, and 1-hr post-training periods, for MBON-γ1pedc > α/β, MBON-γ2α’1, MBON-α2sc, MBON-α’2, MBON-α3 and MBON-α’3 m. Gray dots: data from individual flies. (*P < 0.05 and **P < 0.01; *n* = 12 flies per neuron-type; Friedman ANOVA followed by post-hoc Wilcoxon signed-rank tests with Holm-Bonferroni correction). Source Data

Unlike PPL1-DANs, after training, MBONs-γ1pedc>α/β and -γ2α′1 had decreased CS^+^-evoked responses that returned to near-baseline values about 1 h later, in agreement with previous studies^12^, whereas decreased CS^+^-evoked responses in MBON-α3 persisted for more than 1 h (Fig. 3f and Extended Data Fig. 6). Spontaneous spiking by MBON-α3, but not by other MBONs, increased after training.

## MBON-α3 plasticity encodes long-lived memory and depends on innate odour valence

**Fig. 4:**
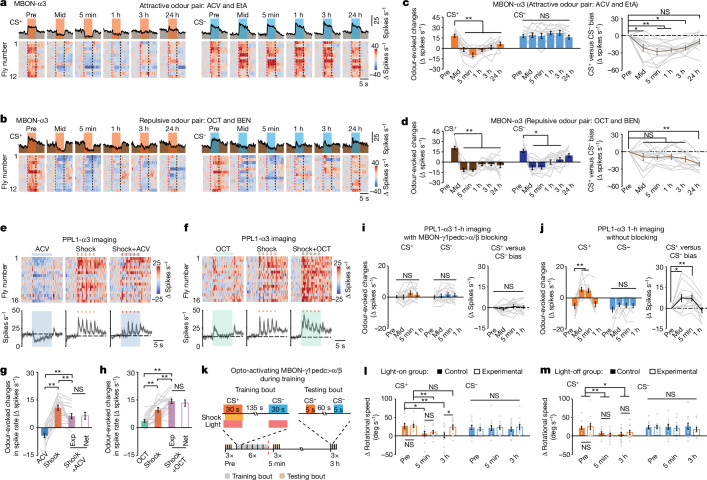
Both innate and learnt valences influence long-lasting plasticity and behaviour. **a**,**b**, Voltage imaging reveals plasticity in MBON-α3 lasting ≥24 h after conditioning with an attractive (**a**; ACV and 1% ethyl acetate) or repulsive (**b**; OCT and 0.3% BEN) odour pair. The protocol was that of Fig. 3d but with additional imaging bouts at 3 h and 24 h after training. Top, time-dependent mean spike rates before, during and after 5-s exposures to CS^+^ (orange) or CS^−^ (blue) odours during the six different imaging sessions. Bottom, odour-evoked changes in MBON-α3 spike rates in 12 flies. Each row shows a single trial of data. **c**,**d**, Left, changes in MBON-α3 spike rates induced by CS^+^ and CS^−^ odours in the six imaging sessions in flies trained with attractive (**c**), or repulsive (**d**) odours. Right, CS^+^ versus CS^−^ bias in evoked spiking, relative to pre-training responses. *P < 0.05, **P < 0.01; *n* = 12 flies. **e**,**f**, Attractive odour attenuates (**e**) but repulsive odour enhances (**f**) punishment-induced spiking by PPL1-α3. Top, spiking changes relative to baseline rates in 16 flies before, during and after 10-s exposures to odours (left; ACV in **e**, blue shading; 1% OCT in **f**, green shading), five electric-shock pulses (red tick marks, middle) or paired odour–shock presentations (right) (one trial per fly for each of the three conditions). Bottom, time-dependent mean spiking responses to each stimulus (shading, s.e.m.). Black dashed lines indicate mean baseline spike rates in the first 5 s of recording. **g**,**h**, Changes (*n* = 16 flies) in PPL1-α3 spiking relative to baseline rates during 10-s exposures to odour (ACV in **g**; 1% OCT in **h**), five shock pulses or paired odour–shock presentations (Exp, purple filled bars). Spiking changes during odour–shock presentations were indistinguishable from the sum of changes induced by the two individual stimuli (Net, purple empty bars). **i**,**j**, Left, changes in PPL1-α3 spike rates in the blocking (**i**; *TH-LexA/13×LexAop-pAce;MB085C/UAS-TnT*) or control (**j**; *TH-LexA/13×LexAop-pAce*) groups, evoked by attractive CS^+^ and CS^−^ odours in four different imaging sessions. Right, CS^+^ versus CS^−^ bias of evoked spiking relative to pre-training responses (Methods; 12 flies per group). **k**, Bottom, timeline for 3-h assay of memory with optogenetic activation for four groups of flies on a trackball. Experimental (*MB085C/UAS-CsChrimson-tdT*) but not control (*MB085C/+*) flies expressed the CsChrimson opsin in MBON-γ1pedc>α/β. Each genotype was split into groups that did (light-on) or did not (light-off) receive optogenetic illumination (30 pulses of 0.5-s red light; red shading) during odour presentation. The protocol is that of Fig. 3a but with memory testing 5 min and 3 h after conditioning. Top, timelines for individual training and testing bouts. Odours (ACV and ethyl acetate) were initially attractive and counterbalanced as CS^+^ and CS^−^ across 12 flies. The results are shown in **l** and **m**. **l**,**m**, At 3 h after conditioning (see **k**), the light-on experimental group had poorer memory performance than the light-on control group (**l**). Flies in both light-off groups (**m**) had normal 3-h memory performance. Plots show rotational speed changes induced by CS^+^ and CS^−^ odours. Empty and filled bars represent experimental and control groups, respectively (12 flies per group). Grey lines (in **c**,**d**,**g**,**h**,**i**,**j**) and grey dots (in **l**,**m**) show data from individual flies, and data are mean ± s.e.m. For these panels, *P < 0.05, **P < 0.01; Friedman ANOVA followed by post-hoc signed-rank tests with Holm–Bonferroni correction for within-group comparisons. In **l** and **m**, across-group comparisons are *U*-tests. Source Data

**Extended Data Fig. 7:**
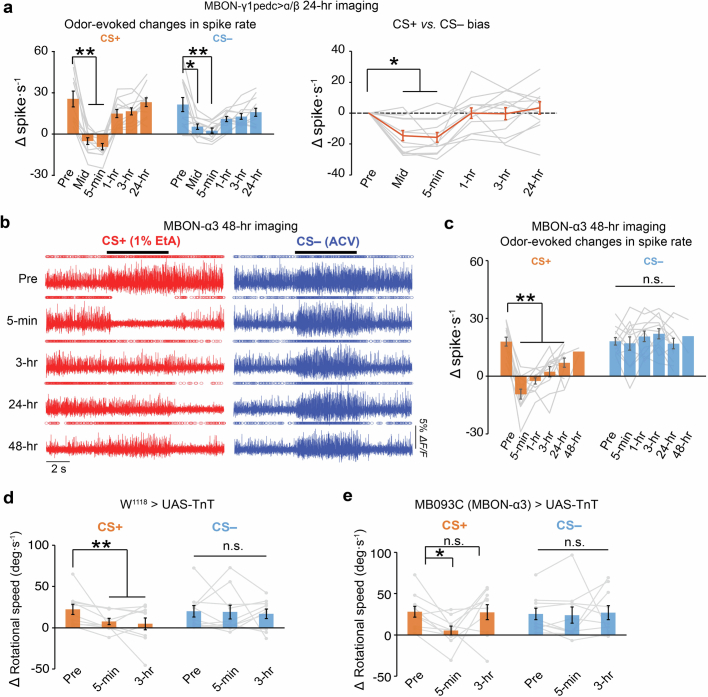
MBON-α3, but not MBON-γ1pedc > α/β, exhibits long-lasting plasticity after olfactory conditioning and is required for 3-hr memory. **a**) *Left*, Mean ± s.e.m. odour-evoked changes in spike rates of MBON-γ1pedc > α/β, induced by the attractive CS^+^ and CS^–^ odours (apple cider vinegar and 1% ethyl acetate; counterbalanced across flies in their assignments as CS^+^ and CS^–^) in the pre-training (Pre), mid-training (Mid), 5-min, 1-hr, 3-hr and 24-hr imaging periods, using the training and imaging protocol of Fig. 3d. *Right*, Changes in the CS^+^ *vs*. CS^–^ bias in evoked spiking responses relative to those in the pre-training session (Methods). (*P < 0.05 and **P < 0.01; *n* = 12 flies per neuron-type; Friedman ANOVA followed by post-hoc Wilcoxon signed-rank tests with Holm-Bonferroni correction). **b**) Example fluorescence voltage traces showing odour-evoked, MBON-α3 spiking responses to 5-s presentations of an innately attractive CS^+^ (1% ethyl acetate, *red*) or CS^–^ odour (apple cider vinegar, *blue*) in the pre-training (Pre) period, or at 5-min, 1-hr, 3-hr, 24-hr or 48-hr after associative conditioning using the training protocol of Fig. 3d. **c**) Mean ± s.e.m. odour-evoked changes in MBON-α3 spike rates, induced by innately attractive CS^+^ and CS^–^ odours in the pre-training (Pre) period, or at the 5-min, 1-hr, 3-hr, 24-hr time points (*n* = 12 flies for each time point; **P < 0.01; Friedman ANOVA followed by post-hoc Wilcoxon signed-rank tests with Holm-Bonferroni correction), or at the 48-hr time-point (n = 2 flies; not included in the ANOVA). **d**, **e**) Mean ± s.e.m. changes in rotational speed induced by CS^+^ and CS^–^ odours in the pre-training (Pre), 5-min and 3-hr testing sessions for *w*^*1118*^ > *UAS-TnT* (**d**) or *MB093C* > *UAS-TnT* (**e**) flies. Both CS^+^ and CS^–^ odours were initially attractive and were either apple cider vinegar (ACV) or 1% ethyl acetate; assignments as CS^+^ and CS^–^ were counterbalanced across 10 flies. *w*^*1118*^ > *UAS-TnT* flies exhibited behavioral conditioning to the CS^+^ but not the CS^–^ odours at 5-min and 3-hr after training. *MB093C* > *UAS-TnT* flies exhibited behavioral conditioning to the CS^+^ odour at 5-min but not at 3-hr after training. Thus, inhibition of synaptic vesicle release in MBON-α3 impedes the longevity but not the formation of associative memory. (*P < 0.05; **P < 0.01; *n* = 10 female flies per genotype; Friedman ANOVA followed by post-hoc Wilcoxon signed-rank tests with Holm-Bonferroni correction). Gray lines in panels **a**, **c**, **d**, **e**: data from individual flies. Source Data

Previous studies have suggested that the γ and α compartments of the mushroom body differentially regulate short- and long-term memories^11,23^. To assess this, our voltage-imaging studies lasted either 24 h or 48 h after associative conditioning (Fig. 4a–d and Extended Data Fig. 7a–c). Depressions of CS^+^-evoked responses endured for less than 1 h in MBON-γ1pedc>α/β neurons, whereas those of MBON-α3 persisted for more than 24 h. To test the necessity of this long-lasting plasticity for long-lasting memory, we blocked neurotransmitter release from MBON-α3; this impaired memory at 3 h but not 5 min after training, verifying the selective importance of MBON-α3 for long-lasting memory (Extended Data Fig. 7d,e).

Next, we examined how innate valences influence long-lasting plasticity by comparing conditioning with attractive versus repulsive odour pairs. After three training bouts with attractive odours, CS^+^- but not CS^−^-evoked responses in MBON-α3 switched from spiking increases to decreases, which became more pronounced after three more bouts (Fig. 4a,c). This plasticity gradually decayed but remained after 24 h. The CS^+^/CS^−^ bias fell below its pretraining level for all postconditioning time points except 24 h.

**Extended Data Fig. 8:**
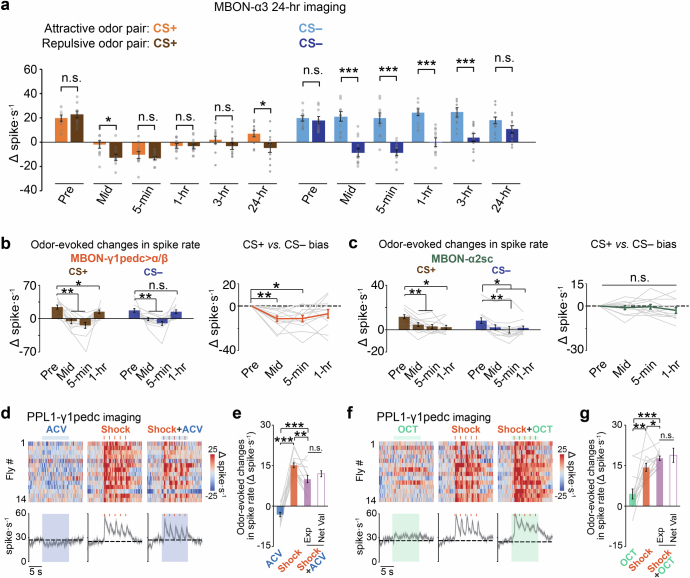
Valence-dependent coding and plasticity: Like PPL1-α3, PPL1-γ1pedc conveys the net valence of jointly presented stimuli, and spiking plasticity in MBON-γ1pedc > α/β, MBON-α2sc and MBON-α3 depends on innate odour valence. **a**) Mean ± s.e.m. odour-evoked changes in MBON-α3 spike rates induced by the CS^+^ and CS^–^ odours at different time-points before, during and after associative conditioning with either a pair of innately attractive (ACV and 1% ethyl acetate (EtA)) or a pair of innately repulsive (OCT and 0.3% benzaldehyde (BEN)) odours. We followed the training and imaging protocol of Fig. 3d but added extra imaging sessions at 3-hr and 24-hr post-training. (*P < 0.05 and ***P < 0.001; *n* = 12 individual flies. Mann-Whitney U-tests). Gray dots: Data from individual flies. **b**, **c**) *Left*, Mean ± s.e.m. odour-evoked changes in spike rates of MBON-γ1pedc > α/β, (**b**), or MBON-α2sc, (**c**), induced by the CS^+^ and CS^–^ odours in the pre-training (Pre), mid-training (Mid), 5-min, and 1-hr imaging periods, using a pair of repulsive odours, OCT and 0.3% benzaldehyde (BEN). *Right*, Changes in the CS^+^ *vs*. CS^–^ bias in evoked spiking responses relative to that of the pre-training session (Methods). These valence-dependent changes in MBON activity patterns (compare to Fig. 3f) might be relayed to PPL1-α3 (Fig. 1a), perhaps amplifying valence-dependent activity in the α3 unit. (*P < 0.05 and **P < 0.01; *n* = 12 flies per neuron-type; Friedman ANOVA followed by post-hoc Wilcoxon signed-rank tests with Holm-Bonferroni correction). **d**) Attractive odour attenuates the punishment-induced spiking responses of PPL1-γ1pedc. *Top*, Changes in PPL1-γ1pedc spike rates relative to baseline levels, immediately before, during and after delivery of either 10-s-exposures to apple cider vinegar (ACV; blue shading; *left*), 5 electric-shock pulses (each 0.2 s in duration with 1.8 s interval between pulses; red tick marks; *middle*), or the paired presentation of ACV and shocks (*right*) to *n* = 14 flies (1 trial per fly for each of the 3 stimulation conditions). *Bottom*, Time-dependent mean spiking rates, averaged over all 14 trials for each stimulus. Dashed lines mark the mean baseline spiking rates, averaged over the first 5 s of recording. Gray shading on the time traces: s.e.m. **e**) Mean ± s.e.m. odour-evoked changes in PPL1-γ1pedc spike rates relative to baseline levels, as measured during 10-s exposures to ACV (blue bar), 5 electric shocks (red bar), or paired presentations of ACV and shocks (purple solid bar). (**P < 0.01 and ***P < 0.001; *n* = 14 flies; Friedman ANOVA followed by post-hoc Wilcoxon signed-rank tests with Holm-Bonferroni correction). Spiking responses to the paired presentations of ACV and shocks were indistinguishable from the sum of the changes induced by the two stimulus-types when each was presented independently (purple hollow bar; *n* = 14 flies; Wilcoxon signed-rank tests). **f**) Repulsive odour enhances punishment-induced spiking responses in PPL1-γ1pedc. *Top*, Changes in spike rates, immediately before, during and after 10-s-exposures to either 1% 3-octanol (OCT; green shading; *left*), 5 pulses of 200-ms-electric-shock (red tick marks; *middle*), or the joint presentation of OCT and shock (*right*) to n = 14 flies (1 trial per fly for each of the 3 stimulation conditions). *Bottom*, Time-dependent mean spiking rates, averaged over all 14 trials for each stimulus. Dashed lines: mean baseline spiking rates, averaged over the first 5 s of recording. Gray shading on the time traces: s.e.m. **g**) Mean ± s.e.m. odour-evoked changes in PPL1-γ1pedc spike rates relative to baseline levels, measured during 10-s exposures to either OCT (green bar), electric shocks (red bar), or joint presentations of OCT and shocks (purple solid bar). (*P < 0.05 and ***P < 0.001; *n* = 14 flies; Friedman ANOVA followed by post-hoc Wilcoxon signed-rank tests with Holm-Bonferroni correction). Spiking responses to the joint presentation of OCT and shocks were indistinguishable from the sum of the changes induced by the two stimulus-types when each was presented independently (purple hollow bar) (*n* = 14 flies; Wilcoxon signed-rank test). Gray lines in **b**, **c**, **e**, **g** denote data from individual flies. Source Data

By comparison, after only three training bouts with repulsive odours, CS^+^ and CS^−^ presentations evoked suppressions of MBON-α3 spiking across >24 h and 3 h, respectively (Fig. 4b,d and Extended Data Fig. 8a). CS^+^/CS^−^ response biases were statistically unchanged until 24 h after conditioning. There were also CS^+^- and CS^−^-evoked suppressions of MBON-γ1pedc>α/β spiking and diminished odour responses in MBON-α2sc, unlike the case with attractive odours (Extended Data Fig. 8b,c). Overall, innate odour valence greatly influenced MBON-α3 plasticity and thereby long-lasting memory.

## MBON-γ1pedc>α/β feedback to PPL1-α3 shapes MBON-α3 plasticity

To investigate long-lasting plasticity in MBON-α3, we studied the bidirectional teaching signals from PPL1-α3 and how PPL1-α3 responds to co-occurring stimuli with similarly or oppositely signed valences. Pairing five shock pulses with either attractive or repulsive odours evoked PPL1-α3 dynamics that combined the specific activity patterns elicited by the odour or shocks individually (Fig. 4e–h). Attractive odours reduced shock-evoked spiking, but repulsive odours did the opposite, in a manner consistent with PPL1-α3 linearly summing its responses to the individual stimuli. PPL1-γ1pedc behaved similarly (Extended Data Fig. 8d–g). Overall, PPL1-DAN spiking conveyed the net valence of paired stimuli, explaining why conditioning with attractive versus repulsive odour pairs yields very different plasticity in downstream MBONs.

We considered candidate circuit mechanisms for net valence coding by PPL1-α3, including feedback from several MBONs^6^, and identified feedback from MBON-γ1pedc>α/β as likely to have a central role. Clues motivating this hypothesis were the matching durations of learning-induced depression of the odour responses of MBON-γ1pedc>α/β and the short-lasting potentiation for PPL1-α3 (Fig. 3e,f), suggesting that depressed inhibitory feedback from MBON-γ1pedc>α/β disinhibits the odour responses of PPL1-α3.

**Extended Data Fig. 9:**
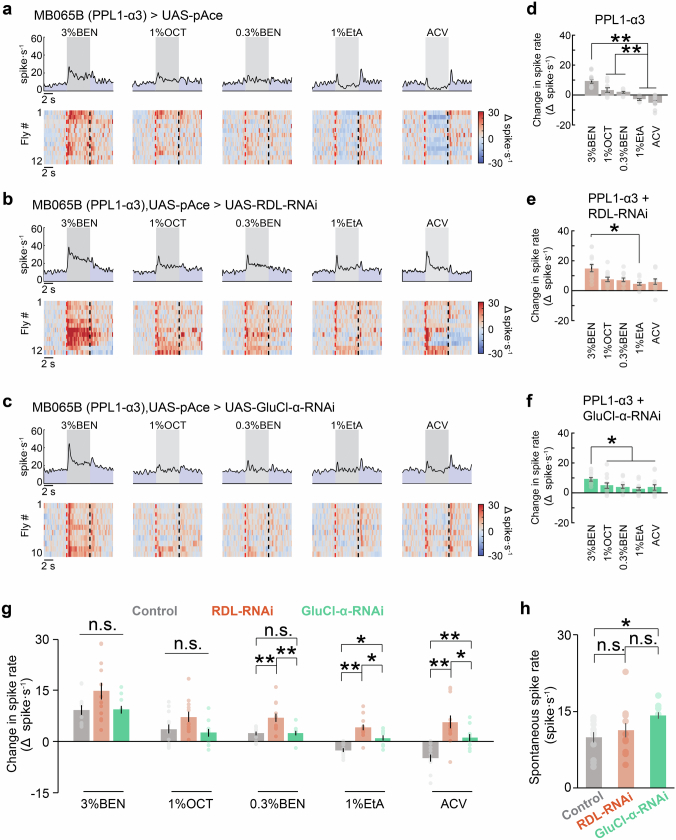
The GABA-A receptor (RDL) and glutamate-gated chloride channel (GluCl-α) are needed for the bidirectional encoding of innate odour-valence by PPL1-α3. **a**–**c**) *Top panels*, Time-dependent mean spiking rates of PPL1-α3 in response to 5 different odours (3% BEN, 1% OCT, 0.3% BEN, 1% EtA, and ACV), in control flies (*MB065B-GAL4* > *20×UAS-pAce*), **a**; flies in which the GABA-A receptor (RDL)^43^ was selectively inhibited in PPL1-α3 using RNAi (*MB065B-GAL4,20×UAS-pAce* > *UAS-RDL-RNAi*), **b**; and flies in which the glutamate-gated chloride channel (GluCl-α)^44^ was selectively inhibited in PPL1-α3 using RNAi (*MB065B-GAL4,20×UAS-pAce* > *UAS-GluCl-α-RNAi*), **c**. Gray shading covers the 5 s of odour presentation. *Bottom panels*, Odour-evoked changes in spike rates, relative to baseline rates, with each row showing single-trial data from an individual fly. Red vertical dashed lines mark the onsets of odour presentation, and the black vertical dashed lines mark the offsets. **d**–**f**) Mean ± s.e.m. changes in PPL1-α3 spike rates during odour exposure (5 s durations) relative to baseline rates in control flies, **d**, flies in the RDL inhibition group, **e**, and flies in the GluCl-α inhibition group, **f**. (*P < 0.05 and **P < 0.01; 12 flies each for the control and GABA-A RNAi groups, 10 flies for the GluCl-α RNAi group; Friedman ANOVA followed by post-hoc Wilcoxon signed-rank tests with Holm-Bonferroni correction). **g**) Mean ± s.e.m. changes in PPL1-α3 spike rates measured during odour presentation (5 s) relative to baseline rates in the control group, the RDL inhibition group, and the GluCl-α inhibition group. (*P < 0.05 and **P < 0.01; 12 flies for the control and GABA-A RNAi groups, 10 flies for the GluCl-α RNAi group; Kruskal-Wallis ANOVA followed by post-hoc Mann-Whitney U-tests with Holm-Bonferroni correction). **h**) Mean ± s.e.m. rates of spontaneous PPL1-α3 spiking for flies in the control group, the RDL inhibition group, and the GluCl-α inhibition group. (*P < 0.05; 12 flies each for the control and GABA-A RNAi groups, 10 flies for the GluCl-α RNAi group; Kruskal-Wallis ANOVA followed by post-hoc Mann-Whitney U-tests with Holm-Bonferroni correction). Gray and colored points in **d**–**h** show measurements from individual flies. Source Data

We tested the role of this feedback in innate odour-valence coding by knockdown of GABA_A_ receptor expression in PPL1-α3 (Extended Data Fig. 9). This disrupted the range and bidirectionality of innate valence coding in PPL1-α3. Downregulating expression of the glutamate-gated chloride channel (GluCl-α) also disrupted innate odour-valence coding in PPL1-α3, suggesting that this coding depends on feedback from multiple MBONs.

Next, we tested the role of feedback inhibition from MBON-γ1pedc>α/β in associative conditioning. Using two genetic expression systems, we imaged PPL1-α3 spiking while blocking MBON-γ1pedc>α/β neurotransmission. In these flies, during training with attractive odours, PPL1-α3 exhibited slight odour-evoked spiking increases that were unaffected by training (Fig. 4i,j). In control flies, odours evoked normal decreases in PPL1-α3 spiking, which after conditioning switched for the CS^+^ to evoked increases. These results suggest that, during learning, the initial depression of CS^+^ responses in MBON-γ1pedc>α/β increases CS^+^ responses in PPL1-α3, which then gates the formation of long-lasting plasticity in MBON-α3 for long-lasting memory.

To test this interpretation, we optogenetically activated MBON-γ1pedc>α/β to maintain its inhibitory feedback signals at a high level during conditioning with attractive odours (Fig. 4k–m). Control flies showed reduced attraction to the CS^+^ at 5 min and 3 h after conditioning, whereas flies receiving MBON-γ1pedc>α/β excitation during conditioning had memory impairments at 3 h but not 5 min after conditioning. This selective impairment shows that removing the strong feedback from MBON-γ1pedc>α/β is crucial for long-lasting memory formation.

## Computational model of valence integration and memory trace interactions

**Fig. 5:**
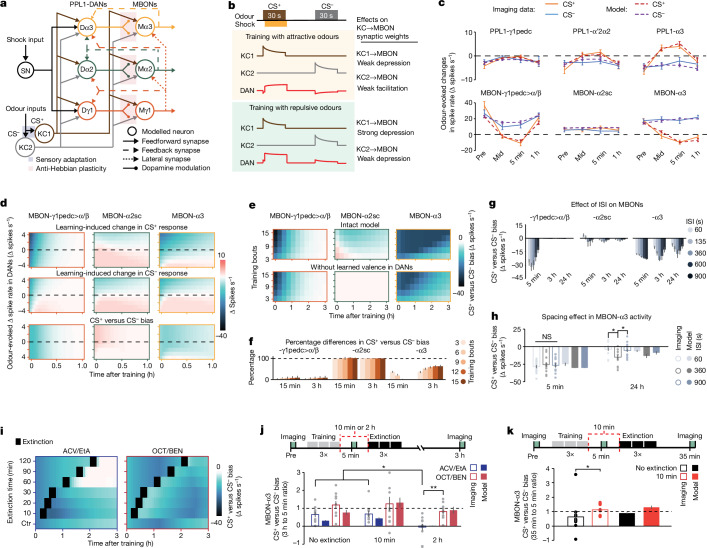
A computational model that captures the interactions between mushroom-body learning units and yields testable predictions. **a**, Connectivity of the model’s three modules (γ1, α2 and α3) and nine neuron types. Kenyon cells (KC1 and KC2) convey olfactory signals to DANs and MBONs (Dγ1, PPL1-γ1pedc; Dα2, PPL1-α′2α2; Dα3, PPL1-α3; Mγ1, MBON-γ1pedc>α/β; Mα2, MBON-α2sc; Mα3, MBON-α3). DANs integrate input from a shock-sensing neuron (SN), olfactory input from KCs and feedback signals from MBONs. Co-activation of a KC and its postsynaptic DAN modifies the weight of the KC→MBON connection through anti-Hebbian plasticity (**b** and Methods). The Supplementary Information lists all model parameter values, obtained by global fits to 86 spike-rate measurements across learning (40 measurements for attractive odours and the relevant DANs and MBONs in Fig. 3e,f except for MBON-α3; 24 measurements for MBON-α3 in Fig. 4c,d; and 22 measurements for aversive odours, comprising 6 in Fig. 2i for DANs and 16 in Extended Data Fig. 8b,c). **b**, Bi-directional anti-Hebbian plasticity rule for KC→MBON connections. In our standard training bout, a 30-s CS^+^ presentation was paired with an unconditioned stimulus (shock) starting 3 s after CS^+^ onset, followed by a 30-s CS^−^ presentation. With either attractive or repulsive odours, KC1 is activated by the CS^+^ and KC2 by the CS^−^, but DAN responses depend on the odour valence. During training with attractive odours, DANs integrate the inhibitory effects of the CS^+^ and excitatory effects of the shock, yielding weak depression of the KC1→MBON synapse. The unpaired CS^−^ presentation suppresses DAN firing, facilitating the KC2→MBON connection. With repulsive odours, joint CS^+^–US presentations strongly activate the DAN, greatly depressing the KC1→MBON synapse. A repulsive CS^−^ mildly depresses the KC2→MBON weight. **c**, Odour-evoked spiking changes (mean ± s.e.m.), from voltage-imaging data (solid lines) and median predictions of the parameter-fitted model (dashed lines) at four time-points in the conditioning protocol of Fig. 3d. Simulated CS^+^ and CS^−^ odours had equal valences, matched to those of ACV and ethyl acetate using odour-evoked changes in DAN spiking before conditioning. For the modelling data in **c**,**f**–**h**,**j**,**k**, *n* = 10,000 simulations per condition, and error bars span 16–84% confidence intervals. **d**, Plots showing how innate valences shape learning in the three MBONs of the model. In each plot, each row has data for one of nine simulated odours, the innate valences of which were specified by evoked changes in DAN spiking (*y* axis). We simulated one training bout (using the protocol of Fig. 3d) with each odour as the CS^+^ (top) or CS^−^ (middle); these six plots show, as a function of time after training, changes in odour-evoked MBON spiking relative to pre-training rates, with time zero denoting immediately after training. The bottom row shows biases between CS^+^- and CS^−^-evoked spiking changes. After one training bout, MBON-γ1pedc>α/β exhibits short-lived depression, MBON-α2sc undergoes a longer-lived plasticity, the sign of which depends on the innate valence of the odour, and MBON-α3 exhibits the longest-lasting plasticity. Extended Data Fig. 10 shows more results. **e**, Simulated biases between CS^+^- and CS^−^-evoked MBON spiking as a function of time after training (*x* axis), given different numbers of training bouts (*y* axis) and with feedback from MBON-γ1pedc>α/β either active (top) or inactivated (bottom). **f**, Changes in CS^+^ versus CS^−^ spiking biases when feedback from MBON-γ1pedc>α/β is removed from the simulations of **e**, for 3–15 training bouts, at 15 min and 3 h after training. **g**, CS^+^ versus CS^−^ spiking biases in the three MBONs of the model, at 5 min, 3 h or 24 h after ten training bouts with simulated CS^+^ and CS^−^ odours of no innate valence, for five different ISI values. **h**, Plasticity is ISI dependent. Plotted are predicted (filled bars, median) and empirically measured (empty bars, mean ± s.e.m.) values of CS^+^ versus CS^−^ spiking bias in MBON-α3 at 5 min and 24 h after six training bouts, using an ISI of 60 s, 360 s or 900 s. Grey dots show data from 14 individual flies. **i**, Simulated MBON-α3 plasticity after extinction training (three bouts of unpaired CS^+^ and CS^−^ presentations). Plots show CS^+^ versus CS^−^ spiking biases over 3 h after three associative conditioning bouts. The top six rows show results for extinction sessions (black squares) occurring at different times after the last conditioning bout, and the bottom row shows results for no extinction. Conditioning used innately attractive odours (left; valences matched to ACV and 1% ethyl acetate) or repulsive ones (right; valences matched to 1% OCT and 0.3% BEN). With attractive odours, extinction training has the greatest effect when it occurs a long time after conditioning. With aversive odours, extinction training is less dependent on timing. **j**, Bottom, simulated (filled bars, median) and measured (open bars, mean ± s.e.m.) values of the CS^+^ versus CS^−^ spiking bias in MBON-α3 at 3 h after conditioning, with either no extinction training or with extinction training (3 bouts) at either 10 min or 2 h after associative conditioning (three bouts). Bias values in **j** and **k** are normalized by their values 5 min after conditioning. Training and extinction used innately attractive (ACV and ethyl acetate) or repulsive (OCT and BEN) odours. Grey dots show data from ten individual flies per group. Top, timeline of training, extinction and imaging. **k**, Bottom, simulated (filled bars, median) and measured (empty bars, mean ± s.e.m.) values of CS^+^ versus CS^–^ spiking biases in MBON-α3 at 35 min after three conditioning bouts with attractive odours, with no extinction training (black) or with three bouts of extinction training at 10 min after conditioning (red bars). Dots show data from ten individual flies per group. Top, timeline of training, extinction and imaging. *P < 0.05, **P < 0.01; Kruskal-Wallis ANOVA followed by post-hoc *U*-tests with Holm–Bonferroni correction for **h** and **j**; *U*-tests for **k**. Source Data

**Extended Data Fig. 10:**
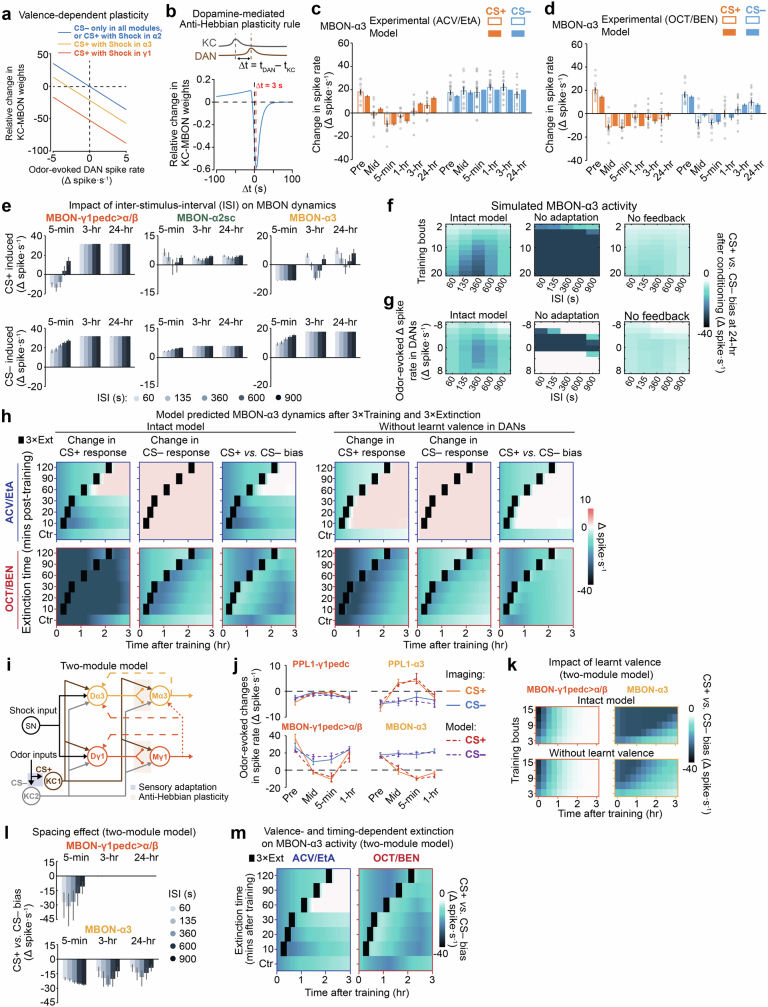
A computational model of the mushroom body captures the interactions between learning units and yields predictions about the dynamics of memory storage and extinction. **a**) Relative changes in KC → MBON weights in the model induced by a single training bout, plotted as a function of the odour-evoked DAN spike rate, which integrates innate and learnt valances. Depending on whether DAN activity is excited or suppressed (*x*-axis), the KC → MBON connection can either become weaker or stronger, respectively. The 3 plots in the graph follow Eq. (5.20) in the Supplementary Information. Weight changes on the *y-*axis are normalized by the ratio of the evoked spike rate in the KC to its maximum spike rate. The CS^+^ is jointly presented with the shock US, thus the integrated valence of both stimuli influences DAN spiking in the α3 and γ1 compartments (orange and red curves). The CS^–^ odour appears alone, but its innate valence also leads to DAN spiking (blue curve). Because PPL1-α’2α2 does not respond to shock, its plasticity curve for a bout of CS^+^–US training is identical to that governing plasticity in any of the modules during a bout of CS^–^ delivery. The *y*-intercept of each plot is the weight change induced by CS^+^–US training with a neutral odor. **b**) Graph schematizing the CS^+^–US timing dependence of the bi-directional, dopamine-mediated, anti-Hebb rule governing plasticity of the KC → MBON connections in the computational model. The curve was based on a prior behavioral study^11^ that used optogenetics to vary the time-scale between KC and DAN activation^60^. The value of Δt (*x*-axis), the interval between the CS^+^ and US presentations, leads to a similar time difference between DAN and KC activation. When a DAN activates before its presynaptic KC, this increases the strength of the downstream KC → MBON connection. In contrast, when a KC activates before its presynaptic DAN, this decreases the strength of the KC → MBON connection. Red dashed line marks the value of Δt (3 s) used for all experiments and simulations in this paper. **c**, **d**) CS^–^- and CS^+^-evoked changes in MBON-α3 spiking at different time points before, during and after associative conditioning, based on our experimental data (*open bars*; means) or the optimized computational model of the MB (*solid bars*; median), for conditioning with attractive (ACV and EtA), **c**, or repulsive odour pairs (OCT and BEN), **d**. For the experimental data, error bars show s.e.m. values over *n* = 12 flies. Gray points: data from individual flies. For the simulation data in panels **c**, **d**, **e**, **j** and **l**, error bars span the 16%–84% C.I. based on results from 10,000 simulations (Methods). **e**) We used the optimized computational model to evaluate how the inter-stimulus-interval (ISI) between CS^+^ and CS^–^ presentations within a training bout influences MBON plasticity at different time points after training. Plots show the median CS^+^- (*top row*) and CS^–^-evoked (*bottom row*) spike rates in each of the 3 MBONs in the model, at 5-min, 3-hr or 24-hr after 10 bouts of training with different ISI values. These studies used hypothetical odour pairs with no innate valence. Our modeling and imaging results reveal a neural embodiment of the ‘spacing effect’ and point to a mechanism; namely, the strength of plasticity in the LTM unit is jointly influenced by the offsetting influences of sensory adaptation and feedback from the STM unit. These two processes have distinct time courses, which jointly set the optimal ISI for LTM formation. When the ISI is less than the optimal ISI duration, sensory adaptation dominates, slowing LTM formation. If the ISI is longer than optimal, the STM decays, weakening LTM induction. This finding motivates future work seeking details of the underlying molecular mechanisms. **f**) To explore how sensory adaptation and neural feedback from MBON-γ1pedc > α/β influence the ISI-dependent plasticity, we used the optimized model to simulate the biases between CS^+^- and CS^–^-evoked MBON-α3 spiking at 24-hr after different numbers of consecutive training bouts (*y*-axis values) using different ISI values (*x*-axis values). These studies used hypothetical odour pairs with no innate valence. Plots show results for the CS^+^ *vs*. CS^–^ bias from simulations of three different conditions, in which: the MB circuitry was intact (*left*); sensory adaptation was removed in the model for both the CS^+^ and CS^–^ odours (*middle*); or feedback signals from MBON-γ1pedc > α/β were absent (*right*). **g**) We used the optimized model to simulate the biases between CS^+^- and CS^–^-evoked MBON-α3 spiking at 24-hr after 10 training bouts of conditioning with one of 9 different hypothetical odour pairs with varying innate valence values (*y*-axis values). The plots show results for the CS^+^ *vs*. CS^–^ bias from simulations of the same three conditions examined in panel **f**. **h**) To examine the effects of extinction training and the role of feedback signals from MBON-γ1pedc > α/β to the DANs, we used the optimized model to simulate extinction training (3 bouts of CS^+^ and CS^–^ presentation but no US presentation) occurring at different times after the last conditioning bout. The plots show the model’s predictions for the learning-induced changes in odour-evoked spiking in MBON-α3 across a 3-hr-duration after 3 bouts of conditioning. The different rows of each plot show odour-evoked spike rates for extinction sessions (marked with black squares) occurring at distinct times after the last training bout; the bottom row of each plot shows results for simulations without extinction training. The simulations used pairs of odours that both had attractive innate valences (matched to those of apple cider vinegar, ACV, and 1% ethyl acetate, EtA; *top plots*) or that both had aversive innate valences (matched to those of 1% 3-octanol, OCT, and 0.3% benzaldehyde, BEN; *bottom plots*). For the innately attractive odours, extinction training has the greatest effect when it occurs at substantial intervals after the end of conditioning. The left three columns show results from simulations using the intact model. The right three columns show results from simulations in which the feedback pathway from MBON-γ1pedc > α/β was inactivated. The difference in efficacy arises from a dynamic competition between the innate appetitive and learnt aversive odour valences, which are both encoded by PPL1-α3 but at relative amplitudes that vary over time and different phases of learning. At times soon after conditioning, PPL1-α3 signals the learnt aversive valence of an innately attractive CS^+^, which allows the CS^+^ to act as its own reinforcer and thus to enhance α3 plasticity, even when the CS^+^ is presented unpaired, without the US. **i**) Diagram showing the neural connectivity in a simplified version of our computational model with only two (γ1 and α3) learning modules. Each of the two modules is shown in a distinct color. Supplementary Fig. 1 of the Supplementary Information shows that, for model parameters common to both the 2-module and 3-module versions of the model, the fitted parameter values are statistically indistinguishable between the two model versions. **j**) After finding optimal parameter values for the 2-module computational model, we examined how well the model’s predictions matched the data used to train it. We compared the odour-evoked changes in spiking for the 2 DAN and 2 MBON neuron-types (*n* = 12 flies per cell-type), as found empirically from voltage-imaging data (*solid lines*; mean ± s.e.m. values) to the model predictions (*dashed lines*; median values) for time points before, mid-way through, and at 5-min and 1-hr after associative conditioning. **k**) To explore how neural feedback from MBON-γ1pedc > α/β to the DANs influences associative conditioning in the 2-module model of panel **i**, we used the optimized model to simulate the biases between CS^+^-evoked and CS^–^-evoked MBON spiking after different numbers of training bouts (*y*-axis values) occurring in immediate succession, with the feedback connections (red dashed lines in panel **i**) that carry learnt valence signals either intact (top row of plots) or removed (bottom row). **l**) We used the 2-module model to evaluate how the inter-stimulus-interval (ISI) between CS^+^ and CS^–^ presentations within a training bout influences MBON plasticity at different time-points after training. The plots show the median CS^+^ *vs*. CS^–^ spiking bias in each of the 2 MBONs in the model, at 5-min, 3-hr or 24-hr after 10 bouts of training using different ISI values. These computational studies used hypothetical odours with no innate valence for either the CS^+^ or CS^–^. The results are highly similar to those in Fig. 5g. **m**) To examine the effects of extinction training in the 2-module model, we used the optimized model and its fitted parameter values to simulate the effects of extinction training (3 bouts of CS^+^ and CS^–^ presentation but no shock presentation) occurring at different times after the last conditioning bout. The plots show model predictions for the CS^+^ *vs*. CS^–^ spiking bias in MBON-α3 across a 3-hr duration following 3 bouts of classical conditioning; the different rows of each plot show the results for extinction sessions (marked with black squares) occurring at distinct times after the last training bout; the bottom row shows results in the absence of extinction training. The simulations used pairs of odours that both had attractive innate valences (*left plot*; valences matched to those of apple cider vinegar, ACV, and 1% ethyl acetate, EtA) or that both had aversive innate valences (*right plot*; valences matched to those of 1% 3-octanol, OCT, and 0.3% benzaldehyde, BEN). For the innately attractive odours, extinction training has the greatest effect when it occurs at substantial intervals after the end of conditioning. For the aversive odours, the effect of extinction training is less dependent on its time of occurrence. Source Data

To analyse how valence signals interact, we modelled three modules (γ1, α2 and α3), interconnected according to the fly connectome^6,7^ (Fig. 5a and Supplementary Information). The nine neurons of the model comprise DANs and MBONs, two Kenyon cells (KCs), which transmit olfactory signals, and a shock-sensing neuron. The DANs integrate shock-related and olfactory signals with MBON→DAN feedback. The bidirectional, anti-Hebbian plasticity of the model was motivated by our findings of bidirectional valence coding and distinct conditioning outcomes using attractive versus repulsive odours. Specifically, KC activation coinciding with DAN activation or suppression respectively weakens or strengthens the corresponding KC→MBON connection (Fig. 5b and Extended Data Fig. 10a,b).

In the model, the initial input strengths of the DANs set their innate odour-valence representations. KC→MBON plasticity allows learnt valences to shape DAN dynamics through MBON→DAN feedback, enabling existing short-term memories to gate long-term memory formation during further training. After conditioning, KC→MBON plasticity decays at rates reflecting the memory-retention properties of each module. Model fits to measured spike rates (Figs. 2i, 3e,f, 4c,d and Extended Data Fig. 8b,c) determined these and other parameter values (Fig. 5c and Extended Data Fig. 10c–m). A roughly 30-min plasticity time constant governs the faster memory decay of the γ compartment, whereas the two α compartments have a roughly 100-min decay. Beyond 3 h after conditioning, plasticity in the α compartments decays with another much slower time constant (Supplementary Information).

**Extended Data Fig. 11:**
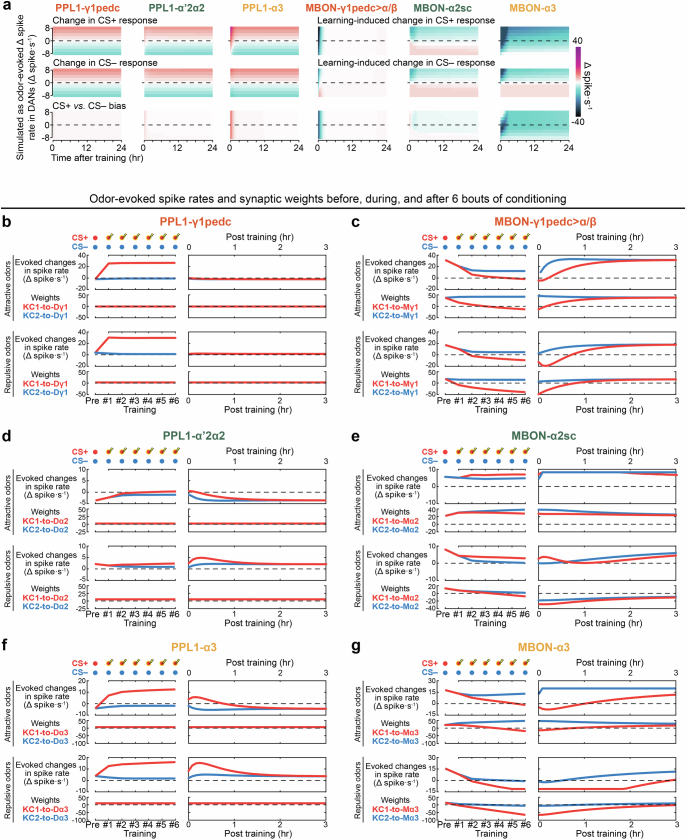
Synaptic weight changes and stimulus-evoked spiking rates in the computational model of the mushroom body after 6 bouts of conditioning with attractive or repulsive odour pairs. **a**) We used the optimized (3-module) model to examine conditioning-induced changes in odour-evoked spiking for the model’s 3 PPL1-DANs and 3 MBONs, after conditioning with one of 17 different hypothetical pairs of odours with varying innate valences as the CS^–^ and CS^+^. Within each odour pair, the two odours had equivalent innate valences. In each of the plots, each row presents data for a single hypothetical odour or odour pair. The innate valence of each odour is specified by the change in DAN spiking that it evokes (*y*-axis values), relative to baseline spiking levels and prior to any associative conditioning. Using the model, we simulated 6 bouts of associative conditioning according to the protocol of Fig. 3d, using each of the 17 odours as either the CS^+^ (top 6 plots) or the CS^–^ (middle 6 plots); these 12 plots show, as a function of time after training, the odour-evoked rates of PPL1-DAN and MBON spiking. The bottom 6 plots show how the biases between CS^+^- and CS^–^-evoked spiking change as a function of time after training. **b**–**g**) We used the optimized (3-module) model to simulate the learning-induced changes in odour-evoked spiking rates and synaptic weights after 6 bouts of conditioning in the PPL1-γ1pedc (**b**), MBON-γ1pedc > α/β (**c**), PPL1-α’2α2 (**d**), MBON-α2sc (**e**), PPL1-α3 (**f**), and MBON-α3 (**g**) neurons. The simulations used pairs of odours that either both had attractive innate valences (top two rows of each panel; valences matched to those of apple cider vinegar, ACV, and 1% ethyl acetate, EtA) or both had aversive innate valences (bottom two rows of each panel; valences matched to those of 1% 3-octanol, OCT, and 0.3% benzaldehyde, BEN). The plots show model predictions for the learning-induced changes in odour-evoked spiking and synaptic weights at pre-conditioning (Pre) and during each of the 6 conditioning bouts (*left plots*), and across a 3-hr-interval after conditioning (*right plots*). The labels of the synaptic weights refer to the cell names, as defined in Fig. 5a. For the 6 conditioning bouts, we simulated neural responses to the paired presentation of the CS^+^ odour and the US, whereas responses to CS^–^- odour were simulated in the absence of the US. PPL1-γ1pedc elevated its spiking rate during paired presentations of the CS^+^ and US, **b**, which led to reduced CS^+^-evoked spiking by MBON-γ1pedc > α/β for up to 1 h after training, **c**. Notably, CS^–^-evoked spiking by MBON-γ1pedc > α/β also decreased during conditioning, mainly due to sensory adaptation. In contrast, the CS^+^-evoked spiking rates of the PPL1-α’2α2 and PPL1-α3 neurons, **d** and **f**, gradually increased across the 6 conditioning bouts and remained higher than CS^–^-evoked spiking rates up to 1 h after training, due to the inhibitory feedback from MBON-γ1pedc > α/β. Under the combined influence of the innate and learnt odour valences encoded by the PPL1-α’2α2 and PPL1-α3 neurons, the downstream MBON-α2sc and MBON-α3 neurons, **e** and **g**, exhibited valence-dependent spiking plasticity that persisted for an hour or more. Source Data

We assessed how odour valences shape plasticity in the model and quantified innate valences of hypothetical attractive or repulsive odorants, respectively, by the negative or positive changes in DAN spiking they evoked. As in real experiments, conditioning involved odour pairs of equal innate valence. One training bout with attractive odours weakly depressed, or even slightly potentiated, odour-evoked spiking in all three MBONs; simulated conditioning with repulsive odours more strongly depressed odour-evoked MBON spiking (Fig. 5d). After six training bouts, MBON-α2sc and -α3 had valence-dependent CS^+^/CS^−^ response biases for more than 24 h (Extended Data Fig. 11). Thus, as found experimentally, the innate valences of sensory cues regulated their plasticity dynamics.

Next, we explored how learnt valences and inhibitory feedback from MBON-γ1pedc>α/β to DANs influence subsequent conditioning. In model versions without this feedback, plasticity decreased by 2–8% in MBON-γ1pedc>α/β, was eliminated in MBON-α2sc, and declined in MBON-α3 by 0–36% at 15 min after conditioning, and by 36–63% at 3 h (Fig. 5e,f). Despite this diminished plasticity of the KC→MBON-α3 connection when MBON-γ1pedc>α/β feedback to PPL1-α3 was removed, at 15 min after conditioning MBON-α3 firing remained almost fully suppressed by odour presentation, as was the case for models with feedback (Fig. 5e). However, without feedback, odour presentation incompletely suppressed MBON-α3 spiking at 3 h after conditioning. Thus, consistent with experimental results, learnt valences promote the long-lasting plasticity of the model in MBON-α3, owing to feedback from MBON-γ1pedc>α/β.

The model made several testable predictions. First, it predicted that synaptic depression levels should depend on the inter-stimulus interval (ISI) between conditioning stimuli. This resembles the ‘spacing effects’ observed in many species, in which learning protocols repeated at longer intervals induce long-term memories more effectively^24,25^. When we increased the ISIs for model training from 60 s to 900 s, short-term (5 min) depression in MBON-γ1pedc>α/β gradually declined (Fig. 5g). MBON-α3 exhibited prominent, ISI-dependent plasticity at 3 h and 24 h after conditioning; plasticity was maximized by an ISI of around 360 s, owing to the countervailing influences of sensory adaptation and MBON-γ1pedc>α/β feedback (Extended Data Fig. 10e–g). In real flies, six training bouts with an ISI of 360 s induced greater long-lasting depression in MBON-α3 than an ISI of 60 s or 900 s (Fig. 5h).

**Extended Data Fig. 12:**
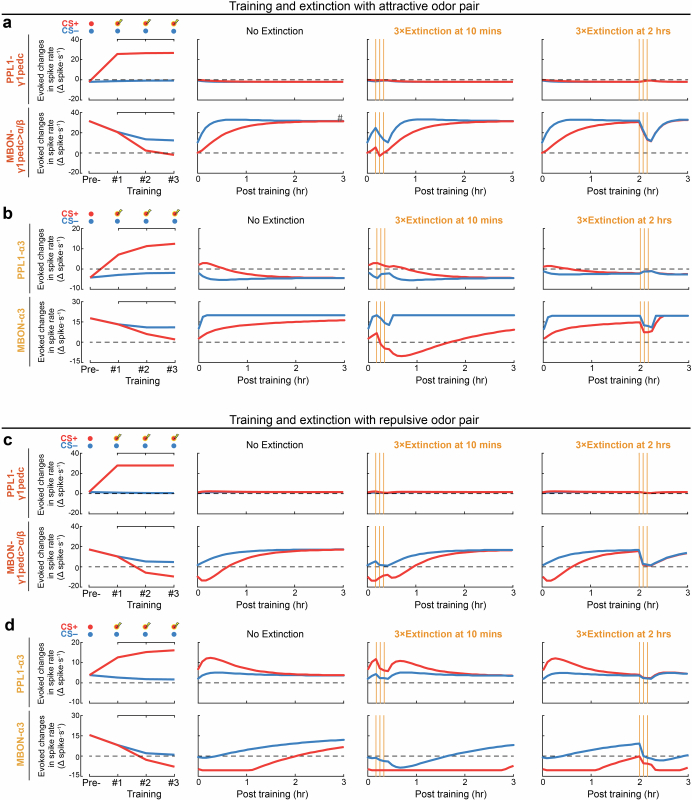
Neural dynamics in short- and long-term memory modules during associative conditioning and extinction. **a**, **b**) We used the optimized 3-module model to simulate the effects of different extinction training protocols (3 bouts of CS^+^ and CS^–^ presentation but no US presentation; see Fig. 5j), in the short-term memory module (PPL1-γ1pedc and MBON-γ1pedc > α/β), **a**, and the long-term memory module (PPL1-α3 and MBON-α3), **b**. The simulations used pairs of odours with attractive innate valences (matched to those of apple cider vinegar, ACV, and 1% ethyl acetate, EtA). The plots show model predictions for the learning-induced changes in odour-evoked spiking at a pre-conditioning time point (Pre) and during 3 successive training bouts (*left column*), across a 3-hr-duration after conditioning with either no extinction training (*middle left column*), or with extinction training at either 10-min (*middle right column*) or 2-hr (*right column*) after the end of conditioning. Vertical orange lines mark the times of 3 successive bouts of extinction training. **c**, **d**) Plots analogous to those of **a** and **b** except that the odour pair used for conditioning had an aversive innate valence. Source Data

The model also predicted that the extinction of a long-lasting memory trace, reflecting MBON-α3 plasticity, should depend on innate odour valence and the time elapsed since conditioning. After repeated conditioning bouts, the CS^+^-evoked responses of the model in PPL1-α3 initially increased but gradually decayed by around 1 h later. Hence, within this first hour, the CS^+^ is not only associated with the unconditioned stimulus (US) in the short-term-learning unit but also acts as a reinforcer in the long-term units (Extended Data Fig. 11d,f). Consequently, postconditioning re-exposures to unpaired CS^+^ and CS^−^ odours influenced MBON-α3 plasticity in a valence- and time-dependent manner (Fig. 5i and Extended Data Fig. 10h). For simulated attractive odours, extinction bouts starting ≥60 min but not ≤30 min after conditioning erased conditioning-induced MBON-α3 depression, as measured 3 h after conditioning (Fig. 5i and Extended Data Figs. 10h and 12). For repulsive odours, extinction bouts induced more modest, transient changes in the CS^+^/CS^−^ response bias of MBON-α3. To test these predictions, 3 h after conditioning we measured MBON-α3 response biases in conditioned flies that either had no extinction sessions or an extinction session at either 10 min or 2 h after conditioning. These measurements supported the model, verifying the predicted valence and time dependence of plasticity after extinction training (Fig. 5j).

In the model, extinction training soon after associative conditioning can paradoxically extend the longevity of plasticity in the long-term memory compartment (Fig. 5i). To test this, we compared the CS^+^/CS^−^ response biases of MBON-α3 in conditioned flies with either no extinction training or with extinction training 10 min after conditioning. Strikingly, extinction training increased MBON-α3 plasticity, assessed 35 min after conditioning (Fig. 5k and Extended Data Fig. 12b). In the model, this effect arises from learnt valences encoded by PPL1-α3, which allows unpaired CS^+^ presentations to reinforce the previously formed plasticity in the long-term memory units, even as they extinguish plasticity in the short-term unit. The learnt valence makes the CS^+^ a self-reinforcer.

## Discussion

**Extended Data Fig. 13:**
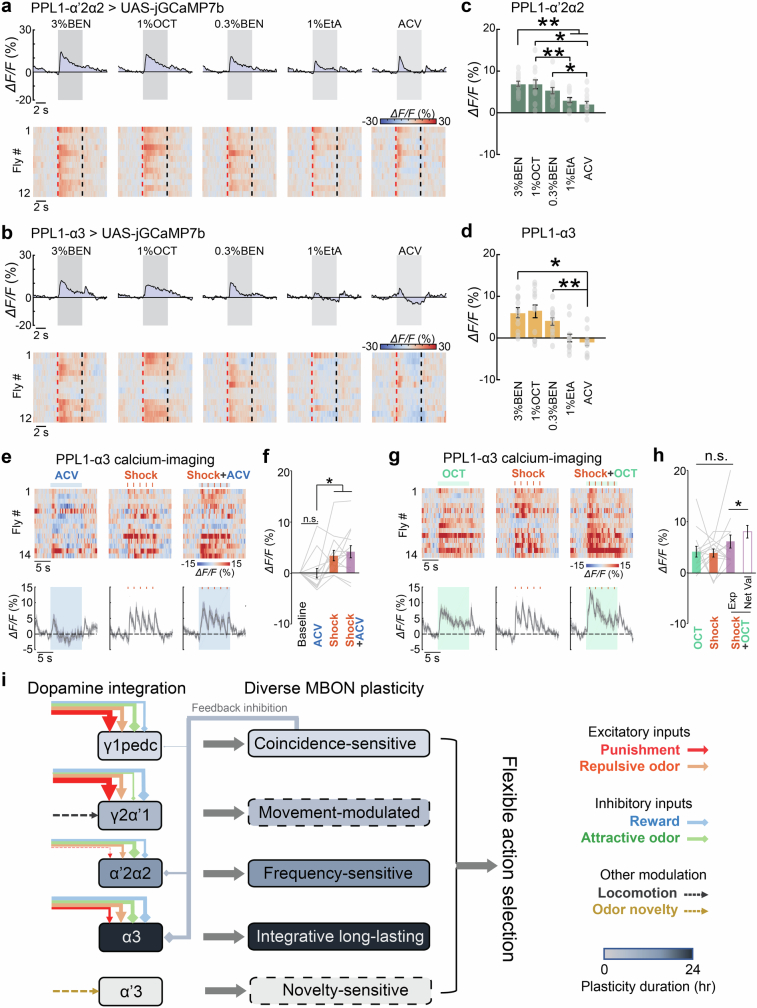
Unlike voltage imaging, Ca^2+^ imaging does not accurately report decreases in spiking and thereby fails to capture the integration of valences encoded by the spiking of PPL1 dopamine neurons. **a**, **b**) *Top plots*, Time-dependent mean fluorescence Ca^2+^ signals (Δ*F/F*) evoked in the PPL1-α’2α2 (*R82C10-LexA* > *13×LexAop-jGCaMP7b*), **a**, and PPL1-α3 neurons (*MB065B* > *20×UAS-jGCaMP7b*), **b**, by 5 different odours (3% BEN, 1% OCT, 0.3% BEN, 1% EtA, and ACV). Gray shading marks the duration of odour presentation. *Bottom plots*, Odour-evoked changes in Ca^2+^ activity in 12 individual flies. Each row shows data from a single fly. Vertical dashed lines mark the onset (red) and offset (*black*) of odour presentation. Comparison to Extended Data Fig. 3f,g shows that Ca^2+^ imaging poorly captures the bidirectional encoding of innate odour valences. **c**, **d**) Mean changes in odour-evoked Ca^2+^ activity relative to baseline levels in PPL1-α’2α2, **c**, and PPL1-α3, **d**, averaged across the 5 s of odour presentation. Error bars: s.e.m. across 12 flies per neuron-type. (*P < 0.05; *n* = 12 flies; Friedman ANOVA followed by post-hoc Wilcoxon signed-rank tests with Holm-Bonferroni correction). Gray dots indicate data from individual flies. **e**) *Top*, Changes in Ca^2+^ activity (Δ*F/F*) in the PPL1-α3 neuron immediately before, during and after 10-s-exposures to apple cider vinegar (ACV; horizontal blue line; *left*), 5 electric-shock pulses (each 0.2 s in duration with 1.8 s interval between pulses; red ticks mark the times of the individual shock pulses; *middle*), or the paired presentation of ACV and shocks (*right*) to *n* = 14 female flies (*MB065B* > *20×UAS-jGCaMP7b*; 1 trial per fly for each of the 3 stimulation conditions). *Bottom*, Traces showing the time-dependent, mean Ca^2+^ activity, averaged over all 14 trials for each stimulus. Horizontal dashed lines: Mean baselines, averaged over the first 5 s of recording. Blue shading covers the periods of odour presentation. Shading on time traces: s.e.m over 14 flies. **f**) Mean ± s.e.m. odour-evoked changes in the Ca^2+^ activity of the PPL1-α3 neuron, as measured during 10-s-exposures to ACV (*blue bar*), 5 electric shocks (*red bar*), or the paired presentation of ACV and shocks (*purple bar*). (*P < 0.05; *n* = 14 flies; Friedman ANOVA followed by post-hoc Wilcoxon signed-rank tests with Holm-Bonferroni correction). Gray lines: Data from individual flies. Changes in the Ca^2+^ activity in response to ACV was statistically indistinguishable from the baseline activity before ACV exposure. Changes in the Ca^2+^ activity in response to shocks alone and joint presentations of ACV and shocks have no significant difference from each other (*n* = 14 flies; Wilcoxon signed-rank test). Comparison to the data of Fig. 4e, g shows that Ca^2+^ imaging poorly captures the ACV-evoked suppression of spiking and the encoding of the net valence of shocks paired with ACV presentation. **g**) Plots analogous to those of **e**, except the odour used (1% OCT) was innately repulsive. **h**) Mean ± s.e.m. odour-evoked changes in the Ca^2+^ activity of the PPL1-α3 neuron, as measured during 10-s-exposures to 1% OCT (*green bar*), 5 electric shocks (*red bar*), or the paired presentation of OCT and shocks (*purple solid bar*). (*n* = 14 flies; Friedman ANOVA followed by post-hoc Wilcoxon signed-rank tests with Holm-Bonferroni correction). Gray lines: Data from individual flies. Changes in Ca^2+^ activity in response to the joint presentation of OCT and shocks were significantly different from the sum of the changes induced by the two stimulus-types, when each was presented independently (*purple hollow bar*) (*P < 0.05; *n* = 14 flies; Wilcoxon signed-rank test). Comparison to the data of Fig. 4f,h shows that Ca^2+^ imaging fails to report the encoding of the net valence of shocks paired with OCT presentation, due to saturation of the Ca^2+^ indicator response during strong neuronal excitation. **i**) Schematic showing how the parallel-recurrent circuitry of the mushroom body (MB) may allow the integration of innate and learnt valence signals in a heterogeneous manner across the different learning units. PPL1-DANs innervate different compartmentalized regions on Kenyon cells (KC) axons and form parallel learning units together with their corresponding downstream mushroom body output neurons (MBONs). Sensory stimuli with innate negative valences, such as punishments (*red*) and repulsive odours (*orange*), heterogeneously excite PPL1-γ1pedc, PPL1-γ2α’1, PPL1-α’2α2, and PPL1-α3. Whereas sensory stimuli with positive valences, such as rewards (*blue*) and attractive odours (*green*), inhibit the 4 PPL1-DANs. During associative conditioning with an aversive US, each individual PPL1-DAN may integrate the valences of stimuli presented concurrently and provide a distinctive teaching signal that drives a depression of KC → MBON synapses. Plasticity in MBON-γ1pedc > α/β lasts for ~1 h and reduces the strength of the inhibitory feedback from MBON-γ1pedc > α/β to PPL1-α’2α2 and PPL1-α3, which in turn facilitates the formation of long-lasting plasticity in those learning units. In addition, the PPL1-γ2α’1 neuron is modulated by the fly’s movement^13,29^, whereas PPL1-α’3 seems to encode odour novelty^21^. Owing to the integration of the innate and learnt valences encoded by PPL1-DAN spiking and to the varying durations of MBON plasticity, the MB’s parallel-recurrent circuitry can enact diverse plasticity patterns that shape fly behavior in a flexible manner. Source Data

Dopamine-based valence integration modulates mushroom-body memory dynamics and seems suited to reserve long-lasting memories, which may be costly energetically^26^, for reliable, frequently encountered associations. Our voltage-imaging studies of these effects involved more than 500 flies and provided some important advantages over Ca^2+^ imaging (Extended Data Fig. 13a–h and Supplementary Discussion). The quantitative, verifiable predictions arising from our model illustrate the potency of combining spike-rate and connectomic data as modelling constraints.

### Valence integration by PPL1-DANs regulates memory dynamics

PPL1-DANs integrate the innate and learnt valences of sensory cues; the tally can be positive or negative, augmenting reports that PPL1-DANs signal negatively valued stimuli^27,28^. During the initial cycles of aversive conditioning, PPL1-DANs sum the innate valences of cue and reinforcer. This leads to the depression of KC→MBON connections, especially in fast (γ1 and γ2) learning units. The net valence signal discourages learning of contradictory associations (such as innately attractive cues paired with punishment), which may not be ecologically reliable and elicit smaller dopamine signals and less plasticity than innately aversive cues paired with punishment. The fast-learning unit (γ1) can rapidly adjust fly behaviour. Through feedback interactions, short-term memory traces in γ1 also gate the formation of long-term traces for repeated, reliable associations. After repeated conditioning, diminished feedback inhibition from γ1 allows DANs of the slower learning units (α2 and α3) to undergo short-lived potentiation of their sensory-evoked responses, which encode the net CS^+^ valence (innate plus learnt) and induce long-lasting plasticity in α2 and α3. Because α2 is more responsive to learnt CS valences than to unconditioned stimulus punishments, this unit seems poised to detect repetitions of cues from prior associative events. In α3, both net odour valence and the unconditioned stimulus drive learning. The γ2 and α′3 compartments respectively respond to locomotion^29^ and odour novelty^21^ and may have specialized learning roles. Sensory data, diverse teaching signals and both short- and long-term memory traces all interact in the mushroom body to execute assorted learning rules in parallel, creating complex memory dynamics (Extended Data Fig. 13i).

### Connectome- and physiology-constrained computational model

Multiple models of the mushroom body include compartmentalized, KC→MBON synaptic plasticity gated by KC–DAN co-activation^15,30–37^ but differ over whether they treat appetitive^33^ or aversive^30,35^ learning (or both^15,31,32,34^), signalling of prediction errors^15,30–32^, interactions between appetitive and aversive learning units^15,31,32,34^, single compartments^35–37^ or both short- and long-term learning units^15,34^, and various lateral and feedback connections. Our model reveals key features of the parallel-recurrent PPL1-DAN/MBON learning system, enacts distinct learning algorithms in short-term and long-term memory (STM and LTM) modules, and makes new predictions. Although several aspects have appeared previously, the model distinctively combines quantitative use of the fly connectome to determine connections, parameter optimization through global fits to spike-rate data, bidirectional plasticity, KC→DAN inputs driving innate odour-valence coding, and MBON→DAN feedback from STM to LTM units for DAN integration of innate and learnt valences, which no previous model we have seen describes.

The bidirectional plasticity implies that KC activation paired with DAN inhibition strengthens the KC→MBON synapse. This model feature is based on the bidirectional encoding of innate valences, distinct plasticity induced by attractive and repulsive odours, and memory-trace extinction induced by unpaired attractive CS^+^ odours. In mammals, negative dopamine signals can encode negative prediction errors^38^. In flies, inactivation or activation of PAM-γ3 DAN, which is normally inhibited by rewards, artificially reinforces appetitive or aversive memories, respectively^39^. Analogous studies should be done with PPL1-DANs.

In our model, anti-Hebbian plasticity supports distinct learning algorithms in different compartments. In the STM (γ1) module, stimulus-evoked DAN signalling is invariant across learning, and MBON-γ1pedc>α/β plasticity relies on the CS^+^–US co-occurrence. The slower-acting (α2 and α3) modules enact prediction-based algorithms by means of CS^+^-evoked dopamine signals that increase over repeated conditioning cycles to convey the learnt valence of the CS^+^ and promote LTM formation. This highlights how inhibitory feedback from MBON-γ1pedc>α/β to PPL1-α′2α2 and PPL1-α3 regulates LTM formation, assigning functions to these recently identified connections^6^.

Excitatory feedback connections from MBON-α2sc and MBON-α3 to PPL1-α3, which might in principle support ‘prediction error’ computations, were fitted to negligible strengths in our model, implying that the α2 unit in the model does not influence long-term plasticity in MBON-α3. Thus, prediction error signals may not be prime drivers of plasticity for our training protocol. Previous work implicates α2 in diverse memory-related functions^11,40^, but studies of α2 plasticity report varying results^23,41^. Here, PPL1-α′2α2 encoded learnt valences after training with odours of either valence, but MBON-α2sc exhibited plasticity only for repulsive odours (Extended Data Fig. 8c). A two-module version of our model with γ1 and α3 units generated almost identical parameter values and LTM dynamics in α3 as the three-module version (Extended Data Fig. 10i–m and Supplementary Fig. 1).

The circuit implementation of valence integration in our model leads to testable predictions about LTM. The model exhibits a spacing effect in memory encoding and predicts enhanced long-lasting plasticity when associative events are spaced at particular optimal time intervals. Mechanistically, plasticity strength in the LTM unit depends on the offsetting influences of sensory adaptation and feedback from the STM unit. These processes have distinct time courses, which jointly set the optimal ISI. For ISI values shorter than optimal, sensory adaptation dominates, slowing LTM formation. For ISI values longer than optimal, the STM decays, weakening LTM induction.

The model also predicts that extinction of a long-lasting memory trace depends on the timing of extinction bouts and the innate valences of the sensory cues. Notably, in flies conditioned with attractive odours, the timing of subsequent re-exposures to the CS^+^ odour strongly influenced MBON-α3 plasticity; extinction bouts 10 min after conditioning strengthened the original α3 plasticity, whereas extinction bouts 2 h after conditioning weakened it (Fig. 5i,j). These effects reflect dynamic competition between innate appetitive and learnt aversive valences, which PPL1-α3 encodes with relative amplitudes that vary across time and learning phases. Soon after conditioning, PPL1-α3 encodes the learnt aversive valence of an innately attractive CS^+^, enabling the CS^+^ to act as its own reinforcer and enhance α3 plasticity.

This competition between innate and learnt valences drives an interplay between STM and LTM that is mediated by feedback inhibition. Unlike previous conceptions, in which spontaneous neural activity during an offline consolidation period transfers memory from STM to LTM modules, in our model, memory is not directly transferred. Instead, feedback from the STM module gates plasticity in the LTM module during training bouts subsequent to the first. Frequent co-occurrences of two stimuli may indicate a reliable relationship that an animal should remember. Crucially, the gating mechanism allows rapid LTM formation in α3 once the repeated association has been detected, unlike in models that encode LTM in slow-changing synapses. The gating also adjusts learning and extinction speeds for odours of different valences; CS^+^–US pairs of opposite valences lead to slower LTM formation plus a memory trace that is more extinction prone. The Supplementary Discussion describes additional valence interactions and explains why CS^−^ stimuli can induce MBON plasticity.

### Outlook

Our model focuses on aversive conditioning and the γ1, α2 and α3 modules, but it neglects modules central to appetitive conditioning^6,11^. Future voltage-imaging experiments should examine the full mushroom body circuit, whether appetitive learning involves valence integration, and paradigms mixing appetitive and aversive reinforcement. How DAN spiking relates to dopamine release should be directly measured, which might clarify plasticity dynamics in α2. Our model neglects plasticity induced by DAN activity in the absence of KC excitation^13,29^, which future models should explore.

Overall, the parallel-recurrent DAN and MBON circuitry flexibly regulates memory by using innate and learnt valences, and exhibits some striking effects, such as self-reinforcement of an unpaired, previously learnt CS^+^. When extrapolated to the mammalian basal ganglia, this finding (Fig. 5k) suggests why habits can be so hard to break. Because many facets of dopamine-based learning are evolutionarily conserved, mushroom-body mechanisms that guide decision-making over multiple timescales may provide insights into how heterogeneous dopamine signalling and recurrent connections between learning modules shape memory dynamics in other species.

## Methods

### Fly stocks

The FlyLight Project Team at Janelia Research Campus provided flies of the Split-GAL4 lines *MB504B-GAL4*, *MB502B-GAL4*, *MB065B-GAL4*, *MB304B-GAL4*, *MB085C-GAL4*, *MB093C-GAL4*, *MB080C-GAL4*, *MB077B-GAL4* and *MB542B-GAL4*. We obtained *R82C10-LexA* (54981), *20×UAS-jGCaMP7b* (79029) and *13×LexAop-jGCaMP7b* (80915) from the Bloomington Drosophila Stock Center. In some experiments, we used other Bloomington fly lines to block neurotransmission through expression of the tetanus toxin light chain^42^ (*UAS-TnT*, 28838) or to knockdown expression of either the GABA_A_ receptor^43^ (*UAS×RDL-RNAi*, 52903) or the glutamate-gated Cl^−^-channel^44^ (*UAS×GluCl-α-RNAi*, 53356). R. Davis (Scripps Institute) provided *TH-LexA* flies and D. Anderson (Caltech) provided *20×UAS-CsChrimson-tdTomato* flies. We outcrossed all strains with *w*^*1118*^ wild-type flies for five generations to minimize differences in genetic background.

To create *20×UAS-Ace2N-mNeon-v2*, *20×UAS-pAce* and *13×LexAop-pAce* flies that express the Ace-2N-mNeon-v2 and pAce FRET–opsin voltage indicators^16,19,45^, we synthesized codon-optimized *Ace2N-mNeon-v2* and *pAce* genes (GenScript Biotech) with a *Drosophila* Kozak sequence before the start codon. We then subcloned the *Ace2N-mNeon-v2* and *pAce* cDNA into the XbaI and XhoI restriction sites of the *pJFRC7-20×UAS-IVS-mCD8::GFP* and *pJFRC19-13×LexAop2-IVS-myr::GFP* vectors (Addgene 26220 and 26224). After verifying the constructed plasmids *pJFRC7-20×UAS-IVS-Ace2N-mNeon-v2*, *pJFRC7-20×UAS-IVS-pAce* and *pJFRC19-13×UAS-IVS-pAce* by sequencing, we used a commercial transformation service (Bestgene) to create two transgenic fly lines for each construct by inserting them into two phiC31 docking sites, the attP40 on the second chromosome and VK00027 on the third chromosome, for further combination with GAL4 or LexA driver lines.

All imaging and behavioural experiments used female flies (3–8 days old at the time of laser surgery). We raised flies on standard cornmeal agar medium with a 12:12 h light:dark cycle at 25 °C and 50% relative humidity. Before surgery or behavioural tests, we chose flies informally in a random manner from a much larger group raised together for all studies; there was no formal randomization procedure for selecting flies. Extended Data Table 1 lists the transgenic fly lines we created for this study, as well as the genotypes and total number of flies used in each imaging and behavioural experiment and for each figure panel. Experimenters were not blind to the genotypes of the flies used. All data collection and analyses were done automatically using computer software that was uniformly applied to all flies irrespective of their genotype.

When imaging flies with more than one fluorescently labelled neuron type, in most cases we focused on one neuron type per fly to achieve recordings with a sufficiently high signal-to-noise ratio. In a subset of flies, we were able to image two or even three neuron types concurrently with satisfactory signal-to-noise ratios. This means the total number of flies imaged is less than the sum of the *n*-values reported in the figure panels. In most experiments, we tested at least 12 flies per neuron type, except that we tested 4–6 flies per type for Extended Data Fig. 3c and 10 flies per condition for Figs. 2b,e and 5j,k and Extended Data Figs. 7d,e and 9c,f–h.

### Odorants

We tested flies’ responses to the following monomolecular odours: ethyl acetate (CAS 141-78-6, Sigma-Aldrich), isoamyl acetate (CAS 123-92-2, Sigma-Aldrich), benzaldehyde (BEN; CAS 100-52-7, Sigma-Aldrich), 1-octen-3-ol (CAS 3391-86-4, Sigma-Aldrich) and 3-octanol (CAS 589-98-0, Sigma-Aldrich). We diluted ethyl acetate, isoamyl acetate, 1-octen-3-ol and 3-octanol into 1% and 10% concentrations and BEN into 0.3% and 3% concentrations (v/v) with mineral oil. We also tested a natural odour, apple cider vinegar (ACV, Bragg).

### Mounting of flies for behavioural, voltage-imaging or optogenetic experiments

In brief, to mount flies for behavioural experiments on a trackball or for in vivo imaging studies of neural activity, we anaesthetized the flies on ice for 1 min. We then transferred them to the cooled surface (around 4 °C) of an aluminium thermoelectric cooling block. When viewing the fly through a dissection microscope (MZ6, Leica) and using a multi-axis stage to manipulate the entire cooling block, we brought the posterior of the fly’s thorax into contact with a fused silica optical fibre 125 μm in diameter (PLMA-YDF-10/125, Nufern) on a custom-made plastic fixture that was secured on the mounting apparatus directly above the fly. We applied around 1 μl of ultraviolet light-curing epoxy (NOA 89, Norland) to the contact point between the fibre and the fly’s thorax and cured the epoxy with ultraviolet for 30 s. Finally, to reduce head motion, we fixed the fly’s head to the thorax using ultraviolet-curable epoxy, after which we considered the fly to be fully mounted. We conducted behavioural testing and imaging experiments in separate sets of flies, because we found that the blue illumination used for voltage imaging substantially disrupted normal, odour-driven fly behaviour.

### Laser microsurgery

To create an imaging window in the fly’s cuticle, we used a laser microsurgery system based on an excimer laser with a wavelength of 193 nm (EX5 ArF, GamLaser), as detailed previously^17,18^. After transferring a mounted fly to the surgery station, we created an optical window in the cuticle by laser drilling a hole 150 μm in diameter (30–40 laser pulses delivered at 100 Hz, 36 μJ per pulse, as measured at the specimen plane). This microsurgical procedure normally removed the cuticle, air sacs and fat bodies, exposing the underlying brain tissue. Occasionally, further rounds of laser dissection or manual cleaning of the cuticle were needed owing to variations in head size and fly age. Immediately after surgery, we applied 1 μl of ultraviolet-curable epoxy (NOA 68, Norland; 1.54 refractive index; approximately 100% optical transmission for wavelengths between 420 nm and1,000 nm) and cured it for 30 s to seal the cuticle opening. We did this under a dissection microscope while using a desktop ultrasonic humidifier (AOS 7146, Air-O-Swiss) to keep the local environment around the fly at around 60% humidity. After mounting the fly, we put a coverslip (22 × 22 mm, number 0, Electron Microscope Sciences) above the fly’s head and placed a small drop (about 1 μl) of water between the coverslip and the fly cuticle.

### High-speed fluorescence voltage imaging

To image neuronal voltage dynamics, we used a custom-built upright epi-fluorescence microscope and a 1.0 NA water-immersion objective lens with a 20× magnification (XLUMPlanFL, Olympus). We used a 503/20 nm excitation filter (Chroma), a 518 nm dichroic mirror (Chroma) and a 534/30 nm emission filter (BrightLine). Using the 500-nm-wavelength module of a solid-state light source (Spectra X, Lumencor), we illuminated the sample with an intensity of 3–7 mW mm^−2^ at the specimen plane. We acquired images at 1,000 Hz, using a scientific-grade camera (Zyla 4.2, Andor) and 2 × 2 pixel binning. For Ca^2+^ imaging experiments with the jGCaMP7b indicator^46^, we used the same set-up and illumination conditions but acquired images at 100 Hz.

### Odour delivery to awake flies

To deliver odours to flies’ antennae, we used a custom-built olfactometer that delivered a constant airflow (200 ml min^−1^) to the fly through either a control path (air passed through mineral oil) or an odour path (air passed through mineral oil with dissolved odorant). Airflow and odours went through a probe needle (1.7 mm inner diameter, Grainger) placed at a 45-degree angle in the horizontal plane and around 3 mm away from the fly’s antennae on the right side (Fig. 2f). Each trial lasted for 15 s, and odour was delivered during the (5 s, 10 s) interval in the trial. For all experiments in which we studied a fly’s responses to multiple different odours, we presented the odours in a pseudo-random order with intervals of at least 2 min between odours. In neural-imaging experiments, as we delivered odours, we imaged neuronal voltage dynamics through the transparent window in the cuticle made above the brain’s right hemisphere by laser microsurgery.

### Electric shock and sucrose delivery

To deliver electric shocks to a fly, we glued a pair of electric wires (0.4 mm diameter; R26Y-0100, OK Industries) to both sides of the thorax with electrically conductive glue (Wire Glue). After the glue dried, the resistance between the two wires was 10–30 MΩ. During each trial, we delivered either three (Fig. 2a,d) or five (Fig. 4g,h and Extended Data Figs. 8d–g and 13d–g) electric shock pulses (0.2 s long, 20 V, delivered 1.8 s apart) using a constant-voltage stimulator (STM200, Biopac Systems), starting 5 s after trial onset.

To image neural responses to sucrose feeding, we positioned the tip of a microlitre syringe (Microliter 701, Hamilton) about 1 mm below the fly’s proboscis. By manually pushing the syringe to deliver around 1 µl of saturated sucrose solution, we allowed the fly to sample the liquid with its proboscis, inducing feeding. In each 15-s imaging trial, we delivered the sucrose solution during the (5 s, 10 s) interval in the trial.

### Measurements of fly locomotion on the trackball

To determine the locomotion of individual flies walking on a trackball (Figs. 2f–h, 3a–c and 4l,m and Extended Data Figs. 3a,b and 7d,e), we used a set-up similar to that of previous studies^47–49^. Specifically, two optical USB pen mice (i-pen mouse, Finger System) were aimed at the equator of an air-suspended, hollow, high-density polyethylene ball (6.35 mm diameter with a mass of around 80 mg; Precision Plastic Ball). The pen mice were 2.3 cm away from the ball and tracked its rotational motion (120 Hz read-out). We converted the pair of digital readouts from the pen mice into a forward displacement on the ball plus a rotational angle for each time bin. We computed the fly’s forward and turning velocities using code written in MATLAB (v.2018b, MathWorks).

### Olfactory conditioning on the trackball

After mounting flies and attaching electric wires to the thorax (see above), we positioned flies on the trackball using a 3D translation stage. Before olfactory conditioning began, we allowed flies to rest on the trackball for at least 30 min to minimize the effect of the cold anaesthesia used during the mounting process. For all conditioning studies, we used two attractive odours, ACV and 1% ethyl acetate, each of which served as either the CS^+^ or the CS^−^ in a counterbalanced manner across the flies used in each group. A 1 h memory experiment comprised one training session and three testing sessions (Fig. 3a–c).

In the training session, we delivered six bouts of CS^+^ and CS^−^ odour pairs to flies sequentially (30 s per odour exposure, with 135 s of fresh air between successive odours). During delivery of the CS^+^ odour, we also administered to the fly 16 electric shock pulses of 20 V amplitude (each pulse of 0.2 s duration, with 1.8 s between successive pulses), starting 3 s after the onset of the CS^+^ odour. This 30-s pairing is longer than the CS^+^–US pairings of 1 s or 5 s often used in conditioning assays for tethered flies^12,50^, because we found that brief pairing durations did not reliably induce behavioural changes that lasted for hours. To capture the time course of learning, we measured behavioural responses to the CS^+^ and CS^−^ before, during and after conditioning, instead of assessing the conditioned response at only one time point, as in the T-maze assay^51^.

In each testing session, we delivered three bouts of CS^+^ and CS^−^ odour pairs to flies (5 s per odour exposure, with 60 s of fresh air between presentations of the CS^+^ and CS^−^ odours and also between bouts). We recorded flies’ forward and turning velocities on the trackball in the three testing sessions (‘before’, 5 min and 1 h). The ‘before’ session occurred 5 min before the training session and assessed the odour-induced behaviour of the flies when they were still naive. The 5 min and 1 h memory-testing sessions respectively began 5 min and 1 h after the end of the training session. In a 3 h memory experiment (Fig. 4l,m and Extended Data Fig. 7d,e), the third testing session occurred 3 h after the training session.

To minimize the bias of flies’ turning behaviour on the trackball, we delivered odours to the left side of flies’ antennae in half of the experiments and to the right side in the other half. Positive values of the fly’s walking speed represent walking forwards, and negative values represent walking backwards. Positive values for rotational velocity indicate that the fly turned towards the direction of odour delivery, and negative values indicate that it turned in the opposite direction.

### Measurements of conditioning-induced neural plasticity

After mounting flies and attaching a pair of wires to deliver electric shocks to the thorax (see above), we allowed flies to rest for more than 30 min before training to minimize the effect of the cold anaesthesia we used in the mounting process. Each 1-h imaging experiment to study memory comprised two training and four testing sessions (Fig. 3d).

Each fly first underwent one bout of imaging before conditioning, in which we examined neural spiking responses to the two odours to be used during conditioning (CS^+^ and CS^−^; each odour was presented for a duration of 5 s with an interval of 120 s between odours). Next, each fly had three bouts of training, in each of which the fly received a paired presentation of the CS^+^ (30 s in duration) and the unconditioned stimulus (electric shocks; 16 pulses of 0.2 s duration, 20 V in amplitude, spaced 1.8 s apart; the first pulse started 3 s after the onset of the CS^+^), and an unpaired presentation of the CS^−^ (30 s in duration, with intervals of 135 s between odours). Then, 5 min after the end of the training bouts, each fly had a mid-training imaging bout to assess the odour-evoked spiking responses. At 5 min after the end of the mid-training imaging bout, each fly had three more bouts of training. Then the fly had another two imaging bouts at 5 min and 1 h after the training. All imaging bouts had the same internal timing structure (Fig. 3d).

In the 24-h memory imaging experiments (Fig. 4a–d and Extended Data Fig. 7a), we used the same protocol for odour and shock delivery as in the 1-h experiments, except that we added two more testing sessions, 3 h and 24 h after training. We kept the flies glued on the optical fibre across the entire 24 h period. To avoid any potential effects caused by food deprivation over this period, we fed the flies with sucrose water at 3 min after the 3 h session and at 30 min before the 24-h and 48-h sessions.

At each time point across associative conditioning, for each neuron type studied we calculated for each fly the differences between its CS^+^- and CS^−^-evoked spike rates, subtracted the bias value measured for the same fly before conditioning in the before-training imaging session, and termed the result the CS^+^ versus CS^−^ bias. We then averaged the bias values across flies. This bias, which, by definition, is zero in the before-training session, was inspired by the two-way choice index that is commonly used to characterize flies’ responses in the T-maze behavioural assay^51^.

In the experiments of Fig. 5j, studying memory extinction, we used three groups of flies: a control group that received memory training (three bouts of CS^+^/US association, as in the training session of 1-h experiments); an ‘early’ extinction group that received memory training and then underwent an extinction session starting 10 min after the end of the training; and a ‘late’ extinction group that received training and then had an extinction session starting 2 h after training. For all three groups, we imaged the neural activity in three testing sessions (before, 5 min and 3 h). The before-training session was 5 min before the training session, and the 5 min and 3 h sessions respectively started 5 min and 3 h after the end of the training session. The extinction session involved three bouts of CS^+^ and CS^−^ odour exposure, as in the training session but without electric shocks. The experiments reported in Fig. 5k had a similar structure, except that there was no late-extinction group and the 3 h imaging session was replaced with an imaging session 35 min after associative conditioning.

### Analyses of imaging data

To extract traces of neuronal voltage activity, we first used an algorithm, NoRMCorre^52^, to correct computationally the raw (1 kHz) fluorescence videos (see the High-speed fluorescence voltage imaging section above) for lateral displacements of the brain. To improve the signal-to-noise ratios of the videos, we applied a denoising algorithm that was based on a singular value decomposition. This involved first reshaping the raw video into a matrix, ${\bf{Y}}\in {{\mathbb{R}}}^{p\times d}$, where *p* is the total number of video frames and *d* is the number of pixels in the field of view. We then decomposed **Y** as a product, **Y** = **UC**, where **U** is a set of *k* low-rank components ${\bf{U}}\in {{\mathbb{R}}}^{p\times k}$, and ${\bf{C}}\in {{\mathbb{R}}}^{k\times d}$ are weighting coefficients. The components **U** are assumed to be semi-unitary, without loss of generality, and were obtained by computing the singular value decomposition of **Y**. The number, *k*, of low-rank components that we retained in **U** was determined by requiring that the set of retained singular vectors captured more than 95% of the variance in the raw video. We then calculated the coefficients as **C** = **U**^***T***^**Y**. For each row of the coefficient matrix, after reshaping it back into a two-dimensional image, we applied the BM3D image-denoising algorithm^53^, which applied a nonlinear thresholding operation to obtain a denoised set of coefficients, $\widehat{{\bf{C}}}$. We determined the denoised video as $\widehat{{\bf{Y}}}={\bf{U}}\widehat{{\bf{C}}}$ and reshaped it back to its original dimensions.

After denoising the fluorescence videos, we manually selected one to three regions of interest that contained the anatomical structures of the targeted cell types expressing the voltage indicator (Extended Data Fig. 1 shows regions of interest for all the fly lines used for imaging). We then computed spatially averaged, time-dependent changes in relative fluorescence intensity, Δ*F*(*t*)/*F*_0_, where *F*_0_ is the mean fluorescence in the region of interest averaged over the entire video and *t* is time. Next, we computationally corrected the resulting fluorescence traces for photobleaching by parametrically fitting a sum of two exponential functions to the mean fluorescence trace, *F*_0_, and then normalizing *F*_0_ by the parametrically fitted trace. To identify neural spikes, we high-pass filtered the Δ*F*(*t*)*/F*_0_ trace by subtracting a median-filtered (40 ms window) version of the trace and then identifying as spikes the local peaks that surpassed a threshold value. Because different cell types had distinct spiking rates and signal-to-noise ratios, we used different threshold values for spike detection in different cell types (more than 3 s.d. for PPL1-DANs and MBON-α2sc, more than 2 s.d. for MBON-γ1pedc>α/β and MBON-γ2α′1, and more than 2.5 s.d. for MBON-α′2, -α3 and -α′3m). We calculated the spiking rate using the number of spikes that occurred in a sliding 100 ms window. Burst ratio was computed as the number of spikes occurring less than 20 ms after the preceding spike divided by the total number of spikes in the trial.

To compute mean optical spike waveforms, we temporally aligned each identified spike in a trial to the time at which its peak value of Δ*F*(*t*)*/F*_0_ occurred. We performed a spline interpolation (10 μs sampling) of the mean waveform, and from this we determined the spike amplitude.

We also used a signal-detection framework to compute the spike-detection fidelity, *d*′, which characterizes the ability to correctly distinguish instances of a spike from background noise fluctuations in the fluorescence trace^19,54^. As we described previously^54^, when we use *N* successive samples of photon counts from a photodetector, *F* = (*F*_1_, *F*_2_, …, *F*_*N*_), to detect spikes, the distribution of *F* follows Poisson statistics in the shot-noise-limited regime. We can use the distribution to express two mutually exclusive hypotheses: the null hypothesis, H^(0)^, which posits the absence of a spike; and the alternative, H^(1)^, which posits that a spike occurred at time zero. The *d*′ value was calculated as *d*′ = (*μ*_*L*_^(1)^*−* *μ*_*L*_^(0)^)/*σ*_*L*_, in which *μ*_*L*_ and *σ*_*L*_ represent the mean and variance, respectively, of the distributions of the log-likelihood ratio, *L*(*f*), for each of the two hypotheses.

The mean, *μ*_*L*_, and variance, *σ*_*L*_, of the distribution of *L*(*f*) under the null hypothesis, H^(0)^, of no spike having occurred, and under the alternative hypothesis, H^(1)^, that a spike occurred, are given by:

$$\begin{array}{l}{\mu }_{L}^{(0)}=\frac{{F}_{0}}{\nu }\mathop{\sum }\limits_{n=1}^{N}\log (1+{s}_{n})-\frac{{F}_{0}}{\nu }\mathop{\sum }\limits_{n=1}^{N}{s}_{n}\\ {\mu }_{L}^{(1)}=\frac{{F}_{0}}{\nu }\mathop{\sum }\limits_{n=1}^{N}(1+{s}_{n})\log (1+{s}_{n})-\frac{{F}_{0}}{\nu }\mathop{\sum }\limits_{n=1}^{N}{s}_{n}\\ {\sigma }_{L}\approx {\sigma }_{L}^{(1)}\approx {\sigma }_{L}^{(0)}=\sqrt{\frac{{F}_{0}}{\nu }\mathop{\sum }\limits_{n=1}^{N}{\log }^{2}(1+{s}_{n})},\end{array}$$

where *ν* denotes the sampling rate, *F*_0_ represents the baseline fluorescence intensity from time periods that contained no neural spike, and *s*_*n*_ is the mean fluorescence signal at each time bin in a time period that contains the averaged waveform of the identified spikes for each imaging trial (*N* = 51 bins, 1 ms per bin).

### Odour classification

For odour classification analysis (Extended Data Fig. 4h), we used PyTorch^55^ (v.1.7.1; www.pytorch.org) to train computational classifiers that identified odours on the basis of patterns of activity in PPL1-DAN or MBON neural populations. Because we imaged different cell types in different flies, we first constructed datasets of the responses of neural populations from ‘virtual flies’^22^ by combining data from the five subtypes of PPL1-DAN or the six subtypes of MBON to produce aggregate PPL1-DAN or MBON population datasets. For each cell type, we used recordings from 12 different real flies and their neural responses to each of five odours. To construct a dataset of PPL1-DAN neural-population odour-evoked responses for an individual virtual fly, we randomly selected one of the 12 real flies studied for each of the *N*_PPL1_ = 5 different PPL1-DAN cell types and combined their odour-evoked responses. This enabled us to create response datasets for 12^5^ different virtual PPL1-DANs, each of which responded to *N*_odours_ = 5 different odours. We used an analogous approach to construct datasets of MBON neural-population odour-evoked responses and combined the data from randomly selected flies for each of the *N*_MBON_ = 6 different MBON types. This approach would have allowed us in principle to create 12^6^ different datasets of virtual MBON population responses, but in practice we constructed only 12^5^ such datasets so we would have an equal number of MBON and PPL1-DAN virtual flies. To create shuffled datasets, we took the same two sets of 12^5^ virtual flies and within each set we randomly reassigned the neural responses across the set of odours.

To create classifiers of odour identity on the basis of the odour-evoked neural-population responses of virtual flies, we randomly assigned 90% of the virtual flies to a training set, 5% of the virtual flies to a validation set, and the remaining 5% to a testing set. We used the validation set to evaluate trained classifiers and tune hyperparameters, but we used the testing set only at the end to determine the rate of correct classifications attained with the optimized classifier. We used linear support vector machines^56^ to create a multiclass linear classifier of odour identity. To perform odour classification using the set of all *N*_odours_ × 12^5^ virtual PPL1-DAN odour-evoked responses, we created a vector classifier function, **f**, for which the value for the *i*-th odour-evoked neural response (1 ≤ *i* ≤ *N*_odours_ × 12^5^) was

$${\bf{f}}({{\bf{x}}}_{i}\,,{W}_{{\rm{PPL}}1-{\rm{DAN}}},{\bf{b}})={W}_{{\rm{PPL}}1-{\rm{DAN}}}\,{{\bf{x}}}_{i}+{\bf{b}},$$

where *W*_PPL1−DAN_ is a matrix of size *N*_odours_ × *N*_PPL1_, **x**_*i*_ is a vector of size *N*_PPL1_ that expresses the PPL1 responses of a specific virtual fly to one of the odours, and **b** is a bias vector of size *N*_odours_. For computational purposes, we rewrote **f** as

$${\bf{f}}\left({{\bf{x}}}_{i}^{{\prime} },W\right)=W{{\bf{x}}}_{i}^{{\prime} },$$

where *W* is a matrix of size *N*_odours_ × (*N*_PPL1_ + 1) comprising *W*_PPL1−DAN_ in its top *N*_PPL1_ rows and the vector **b** in its last row, and ${{\bf{x}}}_{i}^{{\prime} }$ is a vector of size (*N*_PPL1_ + 1) comprising **x**_*i*_ in its first *N*_PPL1_ entries and 1 in its last entry. Given a set of odour-evoked neural responses, the multiclass linear classifier predicted the odour identity, *j*, as ${{\rm{a}}{\rm{r}}{\rm{g}}{\rm{m}}{\rm{a}}{\rm{x}}}_{j}\{{\bf{f}}({{\bf{x}}}_{i}^{{\prime} },W)\}$, that is, according to the entry of the vector classifier function that yielded the maximum value.

To train the model, we optimized *W* by using a hinge loss function that penalized incorrect odour predictions. For the *i*-th odour-evoked response (1 ≤ *i* ≤ *N*_odours_ × 12^5^), its contribution to the total loss was found by summing the penalties incurred for all incorrect classifications

$${{\rm{loss}}}_{i}(W)=\sum _{j\ne {{\rm{odour}}}_{{\rm{true}},i}}\max (0,{\bf{f}}{({{\bf{x}}}_{i}^{{\prime} },W)}_{j}-{\bf{f}}{({{\bf{x}}}_{i}^{{\prime} },W)}_{{{\rm{odour}}}_{{\rm{true}},i}}+1),$$

where the index *j* runs over the individual odours, odour_true,*i*_ refers to the odour that evoked the *i*-th neural response and which is thus the correct classifier result for the *i*-th response, and 1 is used as a margin to help enforce successful classifications. To optimize *W*, we averaged the loss function across individual batches of *N*_batch_ = 200 odour responses chosen randomly without replacement from the full set of *N*_odours_ × 12^5^ responses, with inclusion of an *L*_2_ regularization penalty to minimize the entries of *W*:

$${\rm{l}}{\rm{o}}{\rm{s}}{\rm{s}}(W)=\frac{1}{N}\sum _{i}\sum _{j\ne {{\rm{o}}{\rm{d}}{\rm{o}}{\rm{u}}{\rm{r}}}_{{\rm{t}}{\rm{r}}{\rm{u}}{\rm{e}},i}}max(0,{\bf{f}}{({{\bf{x}}}_{i}^{{\prime} },W)}_{j}-{\bf{f}}{({{\bf{x}}}_{i}^{{\prime} },W)}_{{{\rm{o}}{\rm{d}}{\rm{o}}{\rm{u}}{\rm{r}}}_{{\rm{t}}{\rm{r}}{\rm{u}}{\rm{e}},i}}+1)+\lambda \sum _{k}\sum _{l}{W}_{k,l}^{2}.$$

Here *λ* = 10^−4^ is an *L*_2_ regularization hyperparameter that we optimized empirically using the validation dataset. We then used the Adam optimizer to update the matrix elements of *W*:

$$W:=W-\alpha \times {\rm{Adam}}({\nabla }_{W}{\rm{loss}}(W)),$$

where *α* = 5 × 10^–4^ is a hyperparameter that specifies the learning rate and that was optimized using the validation dataset, and Adam(∇_*W*_loss(*W*)) refers to the Adam optimizer, an extension of stochastic gradient descent that adjusts the learning rate during training to improve convergence. (We used the standard parameter values of the Adam optimizer to adjust the learning rate^57^). To train the classifier, we optimized *W* by updating its matrix elements across ten full passes through the entire set of *N*_odours_ × 12^5^ odour responses. Empirically, we found that extra training did not further improve classification accuracy. We used the same procedures and optimization parameters to train odour classifiers that were based on the set of odour-evoked MBON responses.

Finally, to test the performance of our classifiers, we divided the testing dataset of virtual flies into 120 different sub-testing sets, each with 200 different virtual flies, each with 5 different odour responses. For each sub-testing set, we computed the classification performance as the sum of the number of correctly identified odours divided by 1,000. The box-and-whisker plot of Extended Data Fig. 4h shows the distribution of classification performance values across these 120 different datasets. Note that this classification analysis provides an underestimate of the extent to which individual real flies could classify odour valences on the basis of MBON responses, because our classifiers cannot make full use of the intra-fly correlations between the responses of different MBONs, whereas real flies could in principle use such correlations to create better classifiers.

### Optogenetic studies

To provide all-trans-retinal, which is an essential cofactor for CsChrimson activation^58^, we dissolved all-trans-retinal powder in 95% ethanol to make a 20 mM stock and diluted it with fly food to 400 µM. We collected adult female flies (2 days old) and transferred them to the 400 µM retinal food for 3–5 days before the optogenetics experiments (Fig. 4l,m). To the light-on group of flies, we delivered 30 pulses, each lasting 0.5 s, of red light (625 nm, 0.5 Hz, 25 µW mm^−2^) during CS^+^ and CS^−^ exposures by using a collimated LED (M625L4, ThorLabs), whereas the light-off group did not receive these pulses of illumination.

### Computational model

We simulated computationally a model of the neural circuitry that controls associative conditioning-induced aversive behaviours in *Drosophila*. The model characterized the interactions of KCs, MBONs and DANs in three interconnected learning modules (γ1, α2 and α3) of the mushroom body (Fig. 5a). The KCs sparsely encode the CS^+^ and CS^–^ odour stimuli, and the DANs encode the electric-shock punishments. Dopamine modulates the strengths of the synaptic connections between the KCs and the MBONs, thereby altering the strength of the associative memory. The MBONs gather signals from the KCs to control approach or avoidance motor behaviours (Fig. 5a). Our model used a set of ordinary differential equations to capture how the neural activity patterns of the mushroom body and the synaptic weights change with time. The model thereby describes how associative information is stored and retrieved in the short-term (γ1 module) and long-term memory compartments (α2 and α3 modules) of the mushroom body.

The Supplementary Information presents differential equations that characterize the dynamics of the neural spiking rates and synaptic weights in the model (**§2** and **§3**, respectively, of the Supplementary Information). The network architecture (Fig. 5a) is based on the synaptic connections in the fly brain connectome^7^ (Janelia hemibrain v.1.2.1). If the number of synapses between two neurons is less than 5 in the connectome, we set the corresponding synaptic weight term in the model to be zero. This approximation substantially reduced the number of parameters used in the model. We inferred the values of non-zero synaptic weights by parametric fits of the model to the experimental data on neural spike rates, without further consideration of the number of synapses between neurons (Supplementary Tables 1 and 3).

Concurrent activation of a KC and its corresponding DAN in the model modifies the synaptic weight of the KC→MBON connection according to a bidirectional, anti-Hebbian plasticity rule. The anti-Hebbian rule implies that the KC→MBON synaptic weight decreases if a punishment appears just after odour presentation but increases if the punishment precedes the odour (Extended Data Fig. 10b). In all of our experimental and simulation studies, we used a fixed CS^+^–US interval of 3 s (Figs. 3a,d, 4k,5b and Extended Data Fig. 10b).

The differential equations in sections 2 and 3 of the Supplementary Information form a complete set that models the time-varying neural activity and synaptic weights in the γ1, α2 and α3 modules. We simulated these equations using the MATLAB (Mathworks) function *ode15s()*, which solves the differential equations numerically. However, this approach is time consuming and takes around 14 s to obtain results using each set of parameters. For this reason, we simplified the model using several approximations (see sections 4 and 5 of the Supplementary Information for details). First, we approximated the activation functions of KCs and DANs as linear functions. Second, we assumed that the membrane time constants of KCs, DANs and MBONs (*τ*_KC,*i*_, *τ*_MBON,*j*_ and *τ*_DAN,*j*_) are sufficiently brief (about 10 ms) to allow the spike rates of KCs, DANs and MBONs to attain their steady-state values in associative-conditioning and testing bouts. Third, we assumed that the resting intervals between training and testing bouts or between successive training bouts are much longer than the duration of the training and testing bouts (Figs. 3a,d and 4k), which allowed us to focus our analyses on discrete time points corresponding to the individual training and testing bouts. Finally, we used time-averaged values of KC and DAN spike-rate changes to calculate the changes in the values of the synaptic weights between KCs and MBONs. Using these approximations, we simplified the computational model into a recursive set of equations using discrete time-points (sections 4 and 5 of the Supplementary Information). The time needed to simulate each set of parameters for the simplified model was only about 0.02 s, which is roughly 700-fold faster than the time needed to simulate results for one parameter set of the full model. The recursive formulation of the model also helped us to understand key facets of mushroom-body circuit dynamics and plasticity (sections 4–6 of the Supplementary Information). Notably, Supplementary Information equation 5.20 is plotted in Extended Data Fig. 10a and shows that, given the fixed CS^+^–US interval (3 s) used in all of our experiments and simulations, in the model’s recursive formulation the anti-Hebbian plasticity rule reduces to one in which a linear integration of the innate and learnt valences governs the change in the KC→MBON synaptic weight. The amplitudes of these changes differ between the different compartments of the mushroom body because the different PPL1-DANs respond with distinct amplitudes to the unconditioned stimulus (an electric shock).

By putting the experimental conditions and model parameters (*θ*) into the recursive formulation of the model, we simulated the spike rates of DANs and MBONs in our experiments. Then we optimized the parameters of the model by fitting the model outputs to our experimental spike-rate data (Figs. 2i, 3e,f and 4c,d and Extended Data Fig. 8b,c). The fitted results from the optimized model are shown in Fig. 5c and Extended Data Fig. 10c,d. We assumed that the measured neural spike rates, under all experimental conditions, were governed by independent normal distributions. This assumption allowed us to estimate the optimized values of the model parameters and their confidence intervals (Supplementary Information section 7 and Supplementary Table 3). Using the model and its optimized parameters, we predicted the neural firing rates and their confidence intervals for experiments that had not yet been done. These predictions well matched the subsequent experimental results (Fig. 5g–k).

Because PPL1-α′2α2 does not respond to the unconditioned stimulus electric shock (Fig. 2a,d), and because the α2 compartment does not influence the long-term plasticity of MBON-α3 in the model using the conditioning protocols of this paper, we simplified the model with γ1, α2 and α3 compartments (Fig. 5a) into a two-module model with only the γ1 and α3 compartments (Extended Data Fig. 10i). The two different model variants generated nearly identical predictions for valence-dependent long-term memory formation and extinction in the α3 compartment (Fig. 5e–k and Extended Data Fig. 10k–m). Moreover, for all parameters common to both model variants, the fitted parameter values were statistically indistinguishable between the two-module and three-module versions (Supplementary Table 3 and Supplementary Fig. 1).

### Statistical analyses

We performed all statistical analyses using MATLAB (v.2018b and v.2020b, Mathworks) software. We chose sample sizes using our own and published empirical measurements to estimate effect magnitudes. For statistical testing, we performed non-parametric Kruskal–Wallis and Friedman ANOVAs to avoid making assumptions about normal distributions or equal variances across groups. To perform post hoc pairwise statistical comparisons, we used two-sided versions of the Mann-Whitney *U*-test or the Wilcoxon signed-rank test (respectively abbreviated to ‘*U*-test’ or signed-rank test’ in the figure captions) with a Holm–Bonferroni correction for multiple comparisons.

### Reporting summary

Further information on research design is available in the Nature Portfolio Reporting Summary linked to this article.

## Online content

Any methods, additional references, Nature Portfolio reporting summaries, source data, extended data, supplementary information, acknowledgements, peer review information; details of author contributions and competing interests; and statements of data and code availability are available at 10.1038/s41586-024-07819-w.

## Supplementary information

Supplementary Discussion This file contains the Supplementary Discussion and extra references.

Reporting Summary

Supplementary Information This contains the computational model of PPL1-DAN/MBON dynamics and plasticity, including Supplementary Tables 1–3, Supplementary Fig. 1 and extra references.

Supplementary Video 1 High-speed voltage imaging at single-spike resolution in a behaving fly. Bottom left, video of a fly running in place on a trackball under the microscope objective lens that we used for high-speed fluorescence voltage imaging of the fly’s neural activity. The frame acquisition rate of the fly behaviour movie was 30 frames per second (fps). Bottom right, time traces of the fly’s forward running speed and rotational speed on the trackball. Top left, voltage-imaging movie, recorded simultaneously with the behavioural video, showing the fluorescence changes of the Ace2N-mNeon-v2 voltage indicator in a pair of MBON-γ1pedc>α/β neurons located in opposite hemispheres of the fly brain. The voltage movie was recorded at a frame rate of 500 fps. To synchronize playback with the behaviour movie, we subsampled 1,800 frames of the 30,000 frame-long voltage movie and displayed it at 30 fps. Top right, fluorescence traces and spike raster plots show the spiking dynamics of the two MBON-γ1pedc>α/β neurons.

Supplementary Video 2 Spike back-propagation into the dendritic tree of an MBON-γ1pedc>α/β neuron. A movie of the spike-triggered average fluorescence responses (Δ*F/F*) before, during and after firing of an action potential in the MBON-γ1pedc>α/β neuron of Extended Data Fig. 2a, imaged using the pAce voltage indicator in a behaving fly. The movie shows the time-dependent, mean fluorescence changes averaged over 846 identified spikes from −3 ms to 3 ms relative to the time of the action potential peak within the dendritic tree. Depolarization starts at about −1.5 ms in a region adjacent to one of the axon branches, propagates to the soma and other axon branches, and back-propagates into the dendritic tree. The interval between successive image frames is 50 μs, which we achieved through spline interpolations from the original 1,000 fps acquisition rate. The playback speed is 10 fps. Scale bar: 10 µm.

## Source data

Source Data Fig. 1

Source Data Fig. 2

Source Data Fig. 3

Source Data Fig. 4

Source Data Fig. 5

Source Data Extended Data Fig. 1

Source Data Extended Data Fig. 2

Source Data Extended Data Fig. 3

Source Data Extended Data Fig. 4

Source Data Extended Data Fig. 5

Source Data Extended Data Fig. 6

Source Data Extended Data Fig. 7

Source Data Extended Data Fig. 8

Source Data Extended Data Fig. 9

Source Data Extended Data Fig. 10

Source Data Extended Data Fig. 11

Source Data Extended Data Fig. 12

Source Data Extended Data Fig. 13

## Figures and Tables

**Extended Data Table 1:**
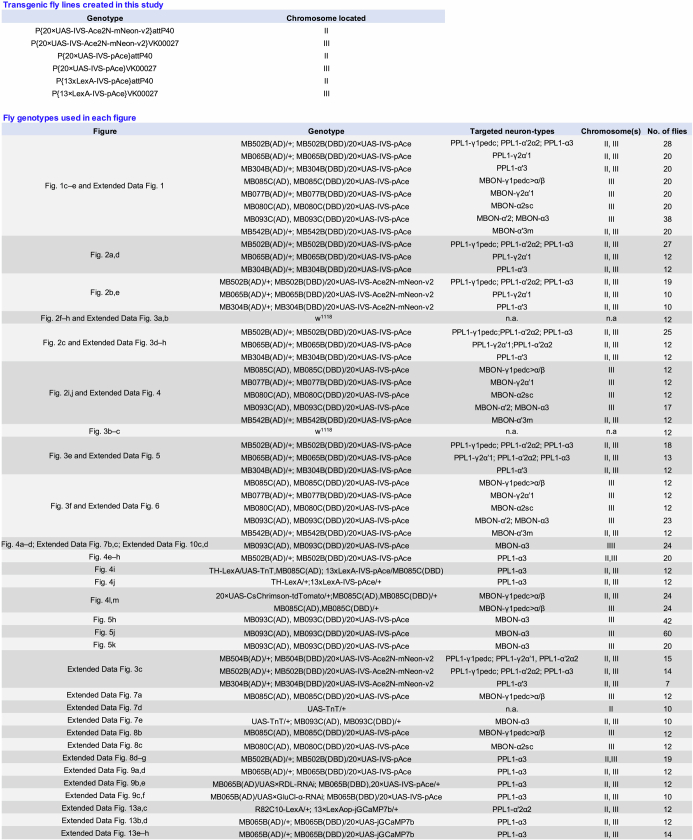
Fly genotypes used in each figure panel

A table of the fly lines created in this study and of the fly genotypes used in each figure panel.

## Data Availability

The Source data files for each figure and Extended data figure in this paper contain data for individual figure panels and statistical results, including for statistical comparisons that are not explicitly marked in the figures. The raw voltage-imaging data are available from the corresponding authors on reasonable request. The neural voltage activity traces are available at Zenodo at https://zenodo.org/uploads/10998457 (ref. ^59^). Source data are provided with this paper.

## Code availability

We used open-source image algorithms for motion correction (NoRMCorre^52^; https://github.com/flatironinstitute/NoRMCorre) and for image denoising (BM3D^53^; https://webpages.tuni.fi/foi/GCF-BM3D). We used the PyTorch^55^ machine learning framework (v1.7.1; www.pytorch.org) for classification analysis. The software code to run our computational model of the MB is available at https://github.com/schnitzer-lab/Luo_Huang_2024_MB_model.
