# Supplementary material for: Dopamine-mediated interactions between short- and long-term memory dynamics
Source: Nature. 2024 Jul 22;634(8036):1141–9. doi: 10.1038/s41586-024-07819-w (PMC11525173; doi:10.1038/s41586-024-07819-w)
Supplement: Supplementary file 1 — This file contains the Supplementary Discussion and extra references. [file 41586_2024_7819_MOESM1_ESM.pdf]

nature portfolio

https://doi.org/10.1038/s41586-024-07819-w

Supplementary information

Dopamine-mediated interactions between short- and long-term memory dynamics

In the format provided by the
authors and unedited

Supplementary Discussion

Benefits of voltage imaging.

Voltage imaging provided 5 advantages for our study, enabling biological findings and the construction and empirical testing of a computational model that would have been unattainable had we used neural Ca^{2+} imaging.

First, unlike Ca^{2+} imaging[1], voltage imaging with FRET-opsin indicators[2–4] allowed neural spikes to be counted digitally, enabling more accurate quantifications of both declines and rises in neural activity. Crucially, we observed declines in PPL1-DAN spiking evoked by attractive odors and rewards (**Fig. 2b**, **c**, **e**), neither of which had been seen by Ca^{2+} imaging[5–7]. The accurate reporting of bidirectional DAN activity changes that reflect the linear integration of innate and learnt valences led directly to our model's incorporation of a bidirectional anti-Hebbian plasticity rule and the gating of long-term memory formation by cues conveying learnt valence information. For comparison, our own Ca^{2+} imaging studies did show the encoding of stimulus valence by PPL1-DANs, but Ca^{2+} activity poorly reported spiking declines and the encoding of net valences when two stimuli were jointly presented (**Extended Data Fig. 13a–h**).

Second, in our longitudinal recordings, voltage imaging allowed direct quantifications of neural firing rates that were unaffected by changes in cells' baseline fluorescence brightness. This robustness allowed us to detect subtle changes in MBON spike rates after conditioning (*e.g.* of MBON-α3; **Extended Data Fig. 6g**).

Third, the direct visualization of neural spikes in voltage imaging facilitated longitudinal monitoring of cell health, thereby benefiting data quality. We found that subtle damage during fly surgery to the neuron to be imaged or adjacent brain tissue could lead to abnormal voltage activity, such as large fluctuations in baseline voltage or an absence of spikes. These abnormalities also

occasionally arose after a few imaging sessions, well after surgery. In such cases, Ca^{2+} imaging would likely not have given as clear a report of a cell's compromised health.

Fourth, voltage imaging's millisecond-scale temporal resolution offers a new means of investigating the genetic and biophysical mechanisms of neuronal signal processing, an area previously hampered by technological constraints. To this point, our research yielded the first evidence of voltage signals in MBON dendritic regions that originated from various subcellular locations and backpropagated to the dendrites (**Extended Data Fig. 2**; **Supplementary Video 2**).

Fifth, accurate quantifications of neural spike rates benefit computational modeling and empirical validation of a model's predictions. Notably, the linear summation of valences by PPL1-DANs, as revealed by voltage imaging (**Fig. 4e–h**), is a crucial property of the learning rule in our model (**Supplementary Appendix, Eq.** (**5.20**)). Further, our model was parametrically fitted to mean spike rate values determined by voltage imaging, which then yielded predictions of spike rate changes induced by training protocols we had not yet tried. We were then able to test and verify these predictions experimentally (**Fig. 5g–k**), because we could directly compare the model-predicted and measured firing rates. The nonlinear $[Ca^{2+}]$ sensitivity of GCaMP, the present most widely used Ca^{2+} indicator, would have clouded these comparisons.

Overall, through its use of voltage-imaging in >500 flies, our work sets an experimental standard for what is now feasible in fly neurophysiology research.

Origin of innate valence coding in PPL1-DANs.

While our data show the importance of odor cues' innate valences to memory formation, it is unclear how PPL1-DANs gain their representations of innate valence. Various cell-types, including olfactory projection, MB anterior paired lateral, Kenyon cells and MBON neurons, send olfactory information to PPL1-DANs[8–11]. Notably, RNAi-mediated downregulation in the PPL1-

DANs of either the GABA-A receptor or the glutamate-gated Cl⁻-channel (GluCl-α) disrupted innate valence coding (**Extended Data Fig. 9b–h**). Thus, representations of innate and learnt valence by PPL1-DANs may be interconnected, owing to the GABAergic feedback from MBON-γ1pedc>α/β. Glutamatergic neurons, such as MBON-β1>α, may also contribute to DAN innate valence coding[10]. In sum, innate valence coding may result from a delicate balance between multiple excitatory and inhibitory inputs.

Effects of conditioning on PPL1-γ1pedc.

Our computational model helps to clarify some curious empirical results. Namely, associative learning with attractive odors leads to positive, CS^+-evoked responses in PPL1-α2 and -α3 but not -γ1pedc (**Fig. 3e**). Although PPL1-γ1pedc receives strong feedback inhibition from MBON-γ1pedc>α/β, as seen in connectomic data, the fitted value of the synaptic weight between these cells is surprisingly weak in our model (**Supplementary Table 3**). Crucially, the fitted weight value represents a functional connection strength that accounts for the combined impact of direct and indirect connections. The low weight value suggests that indirect connections between these two cells may exert offsetting effects, minimizing the impact of MBON-γ1pedc>α/β spiking plasticity on PPL1-γ1pedc.

Plasticity induced by unpaired CS^- stimuli.

Another point clarified by the model relates to the time-dependent plasticity of MBON responses to CS^- stimuli, especially for aversive odors (**Fig. 4b**,**d**; **Extended Data Fig. 8b**,**c**). Our model accounts for this phenomenon using two mechanisms, sensory adaptation and dopamine-mediated plasticity. Repeated exposures to CS^- odors diminish the evoked responses of cell-types upstream of the MB; this adaptation effect is valence-independent and recovers quickly in the model with a characteristic time-constant. Additionally, aversive CS^- odors co-activate KCs and DANs, leading

to a more lasting decline in the KC→MBON weights.

Interactions between aversive associative memory circuits and other forms of memory. Previous work revealed that the γ1 compartment gates two different memory consolidation pathways, which promote either anesthesia-resistant or long-term memory[12]. Interestingly, internal states, such as hunger[13,14] or mating[15,16], seem capable of modulating the function of the γ1 compartment. These findings suggest that different motivational states might have distinct influences on the various direct and indirect feedback pathways that shape PPL1-γ1pedc activity, enabling a flexible regulation of this neuron's dynamics and capabilities for sculpting memory to suit situational needs.

Additionally, the γ1 and α3 compartments contribute to the consolidation and retrieval of appetitive long-term memories[17,18], implying that the interactions between short- and long-term memory traces may involve both the aversive and appetitive memory systems. Notably, recent work[19–21] found interactions between PPL1-DANs and PAM-DANs implicated in appetitive learning[22–24]. Like PPL1-DANs, PAM-DANs appear to encode reward and punishment valences bi-directionally[25]. Future studies should explore the full range of connections in the MB and whether PAM-DANs guide learning using analogous principles of valence integration to those implemented by PPL1-DANs.

References for Supplementary Discussion

1. Chen, T.-W. *et al.* Ultrasensitive fluorescent proteins for imaging neuronal activity. *Nature* **499**, 295–300 (2013).
2. Gong, Y. *et al.* High-speed recording of neural spikes in awake mice and flies with a fluorescent voltage sensor. *Science* **350**, 1361–1366 (2015).
3. Kannan, M. *et al.* Fast, in vivo voltage imaging using a red fluorescent indicator. *Nat. Methods* **15**, 1108–1116 (2018).
4. Kannan, M. *et al.* Dual-polarity voltage imaging of the concurrent dynamics of multiple neuron types. *Science* **378**, eabm8797 (2022).
5. Cohn, R., Morantte, I. & Ruta, V. Coordinated and Compartmentalized Neuromodulation Shapes Sensory Processing in Drosophila. *Cell* **163**, 1742–1755 (2015).
6. Siju, K. P. *et al.* Valence and State-Dependent Population Coding in Dopaminergic Neurons in the Fly Mushroom Body. *Curr. Biol.* **30**, 2104-2115.e4 (2020).
7. Kato, A., Ohta, K., Okanoya, K. & Kazama, H. Dopaminergic neurons dynamically update sensory values during olfactory maneuver. *Cell Rep.* **42**, 113122 (2023).
8. Aso, Y. *et al.* The neuronal architecture of the mushroom body provides a logic for associative learning. *Elife* **3**, e04577 (2014).
9. Takemura, S.-Y. *et al.* A connectome of a learning and memory center in the adult Drosophila brain. *Elife* **6**, (2017).
10. Li, F. *et al.* The connectome of the adult Drosophila mushroom body provides insights into function. *Elife* **9**, (2020).

11. Scheffer, L. K. *et al.* A connectome and analysis of the adult Drosophila central brain. *Elife* **9**, (2020).

12. Plaçais, P.-Y. *et al.* Slow oscillations in two pairs of dopaminergic neurons gate long-term memory formation in Drosophila. *Nat. Neurosci.* **15**, 592–599 (2012).

13. Krashes, M. J. *et al.* A neural circuit mechanism integrating motivational state with memory expression in Drosophila. *Cell* **139**, 416–427 (2009).

14. Tsao, C.-H., Chen, C.-C., Lin, C.-H., Yang, H.-Y. & Lin, S. Drosophila mushroom bodies integrate hunger and satiety signals to control innate food-seeking behavior. *Elife* **7**, (2018).

15. Scheunemann, L., Plaçais, P.-Y., Dromard, Y., Schwärzel, M. & Preat, T. Dunce phosphodiesterase acts as a checkpoint for Drosophila long-term memory in a pair of serotonergic neurons. *Neuron* **98**, 350-365.e5 (2018).

16. Scheunemann, L., Lampin-Saint-Amaux, A., Schor, J. & Preat, T. A sperm peptide enhances long-term memory in female Drosophila. *Sci. Adv.* **5**, eaax3432 (2019).

17. Plaçais, P.-Y., Trannoy, S., Friedrich, A. B., Tanimoto, H. & Preat, T. Two pairs of mushroom body efferent neurons are required for appetitive long-term memory retrieval in Drosophila. *Cell Rep.* **5**, 769–780 (2013).

18. Pavlowsky, A., Schor, J., Plaçais, P.-Y. & Preat, T. A GABAergic Feedback Shapes Dopaminergic Input on the Drosophila Mushroom Body to Promote Appetitive Long-Term Memory. *Curr. Biol.* **28**, 1783-1793.e4 (2018).

19. Felsenberg, J., Barnstedt, O., Cognigni, P., Lin, S. & Waddell, S. Re-evaluation of learned information in Drosophila. *Nature* **544**, 240–244 (2017).

20. Felsenberg, J. *et al.* Integration of parallel opposing memories underlies memory extinction. *Cell* **175**, 709-722.e15 (2018).

21. Jovanoski, K. D. *et al.* Dopaminergic systems create reward seeking despite adverse consequences. *Nature* **623**, 356–365 (2023).

22. Aso, Y. & Rubin, G. M. Dopaminergic neurons write and update memories with cell-type-specific rules. *Elife* **5**, (2016).

23. Liu, C. *et al.* A subset of dopamine neurons signals reward for odour memory in Drosophila. *Nature* **488**, 512–516 (2012).

24. Burke, C. J. *et al.* Layered reward signalling through octopamine and dopamine in Drosophila. *Nature* **492**, 433–437 (2012).

25. Weber, D., Vogt, K., Miroschnikow, A., Pankratz, M. & Thum, A. S. Four individually identified paired dopamine neurons signal taste punishment in larval Drosophila. *Elife* (2023) doi:10.7554/elife.91387.1.
