# Supplementary material for: Dopamine-mediated interactions between short- and long-term memory dynamics
Source: Nature. 2024 Jul 22;634(8036):1141–9. doi: 10.1038/s41586-024-07819-w (PMC11525173; doi:10.1038/s41586-024-07819-w)
Supplement: Supplementary file 3 — This contains the computational model of PPL1-DAN/MBON dynamics and plasticity, including Supplementary Tables 1–3, Supplementary Fig. 1 and extra references. [file 41586_2024_7819_MOESM3_ESM.pdf]

Supplementary Appendix |

Computational model of PPL1-DAN/MBON dynamics and plasticity

Contents

§1. Overview....2

§2. A neural network model comprised of KCs, DANs and MBONs....4

§3. Modeling synaptic plasticity: sensory adaptation and an anti-Hebbian learning rule....8

§4. Mathematical simplifications of the neural network dynamics....11

§5. Mathematical simplifications of the synaptic plasticity dynamics....14

§6. Theoretical analysis of the recurrent circuitry connecting DANs and MBONs....20

§7. Estimating the parameter values of the model....22

§8. Supplementary Tables and Figure....26

Supplementary Table 1 | Synapse numbers in the MB circuit of the fly connectome....26

Supplementary Table 2 | Empirically measured parameter values input directly into the model....27

Supplementary Table 3 | Definitions and values of parameters found by model fitting....28

Supplementary Figure 1 | Comparisons of parameter values in the 2- and 3-module model variants. 29

§9. References....30

§1. Overview.

To simulate associative conditioning and extinction processes in *Drosophila melanogaster*, we created a computational model of the *Drosophila* mushroom body's neural circuit dynamics. The model describes the activity and plasticity patterns of Kenyon cells (KCs), mushroom body output neurons (MBONs), and dopamine neurons (DANs) in 3 interconnected learning modules (γ1, α2, and α3) of the mushroom body (MB) (**Fig. 5a**). The KCs sparsely encode the odor stimuli used in our conditioning experiments. The DANs encode shock punishments received by the fly and modulate the strengths of synaptic connections between KCs and MBONs, thereby shaping the storage and extinction of memories stored in the MB. The MBONs gather signals from the KCs to control motor behaviors. The model thus depicts how associative information is stored and retrieved in both the short-term (γ1 module) and long-term (α2 and α3 modules) learning compartments, including via the interactions between compartments. The implementation of associative learning and memory in the model depends crucially on both the network architecture, which is based on the fly connectome, and two different forms of neural plasticity, sensory adaptation that affects the incoming olfactory signals and anti-Hebbian plasticity that modifies the strengths of KC→MBON synapses. As the modeling results reveal, the feedback connection from the output of the γ1 module to the input of the α3 module is pivotal for a previously unknown gating effect, through which an existing short-term memory trace gates the formation of a long-term memory. Specifically, after a short-term associative memory trace has been formed, during subsequent conditioning the expression of the short-term memory trace gates the initial encoding of the long-term memory trace. This gating effect is distinct from memory consolidation, in that the gating is only active during additional bouts of conditioning, whereas memory consolidation typically occurs offline, between bouts of conditioning. Experimental data strongly support the model's predictions regarding the influence of this feedback-mediated gating (**Fig. 4i–m**).

In **§2**, we first model the circuit comprised of KCs, DANs and MBONs as a recurrent network by using data from the fly connectome[1,2] to define the set of neural connections in the model. We mathematically model the dynamical interactions between the different neurons in the model using a coupled set of ordinary differential equations, one equation for each neuron.

In **§3**, we mathematically model the two forms of plasticity. We implement an adaptation of olfactory sensory neuron activity, such that sensory neurons decrease the amplitudes of their odor-evoked responses during a sustained odor presentation (**Fig. 5b**); further, after the termination of an odor cue, these neurons gradually recover their odor sensitivity[3]. In addition to this form of sensory adaptation, we implement an anti-Hebbian plasticity rule that modifies the strengths of KC→MBON synaptic connections. Specifically, if a KC spikes just before its corresponding DAN activates, the strength of the corresponding KC→MBON connection decreases; if the KC spikes just after DAN activation, the strength of the KC→MBON connection increases[4,5] (**Extended Data Fig. 10b**). The inclusion of both forms of plasticity in the model is important to account for the full range of phenomena seen in our experimental data. For instance, both forms of plasticity are needed for the model to capture the influence of odor valence on the formation and extinction of long-term memory in the α3 compartment (**Fig. 5g–k** and **Extended Data Fig. 10l, m**).

In **§4** and **§5**, we describe several mathematical simplifications and approximations that we used to speed the solution of the ordinary differential equations developed in **§2** and **§3**. While these differential equations provide a rigorous and complete definition of the model, in practice we found that full simulations of the model dynamics were unduly time-consuming. Using the fact that our experiments had temporally defined bouts of conditioning, rest, and extinction training, one can simplify the differential equations governing the network interactions and plasticity processes into a set of recursive equations, for which the discrete time steps correspond to the different bouts of our experiments. In **§4**,

we first transform the differential equations governing the neural dynamics into recursive equations. We also use several additional approximations to simplify the activation functions of the KCs, DANs, and MBONs. In **§5**, we transform the differential equations that describe the plasticity dynamics into recursive equations. This transformation makes use of the fact that our experiments had a fixed interval (3 s) between the onset of the CS^+ odor stimulus and that of the US electric shock. This fixed interval implies that, by design, our experiments did not explore how anti-Hebbian plasticity at the KC→MBON synapse depends on the CS^+–US interval; the fixed interval also reduces to two the number of anti-Hebbian plasticity-related parameters needed to fit our spiking data. Readers wishing to apply our model to more variable experimental conditions should use the general formulations of **§2** and **§3**, while those seeking mainly to understand our fits of the model to experimental data can concentrate on **§4** and **§5**.

In **§6**, we analyze the recurrent feedforward and feedback interactions between the DANs and MBONs in the model, with the aim of understanding how a previously formed short-term memory gates the formation of a long-term memory during subsequent rounds of associative conditioning. In **§7**, we describe the statistical procedures used to fit model parameters to the experimental data, for both the full, three-compartment version of the model and a two-compartment version in which the α2 compartment is omitted. **§8** presents tables showing how the fly connectome constrained the neural connections in the model, the values of numerical parameters that we input directly into the model, and the values of parameters determined for both model versions through statistical fits to the experimental data.

§2. A neural network model comprised of KCs, DANs and MBONs.

In this section, we formulate a recurrent neural network to characterize the dynamics of the individual neurons in the model and their interactions. The recurrent circuit architecture of the model is based directly on the physical connections revealed in recent electron microscopy studies of the fly brain connectome[1,2] (**Fig. 5a**).

The fly connectome data show the feedback and feedforward interactions between MBONs and DANs. For each MBON→DAN or MBON→MBON interaction, if the number of synapses between each pair of cells is <5 in the electron microscopy data[2], we set the corresponding synaptic weights in our model to be zero (**Supplementary Table 1**). However, after applying the threshold, we did not use the numbers of synapses found in the connectome data to determine the model's non-zero synaptic weight values (**Supplementary Tables 1, 3**). The rationale for this choice was that we did not want to over-constrain or oversimplify the important role that synaptic plasticity might play in setting the functional strengths of the MB neural connections. Instead, we determined the values of all non-zero synaptic weights in the model through parametric fits to the spiking rate data recorded experimentally. We did, however, set the signs of the MBON→DAN and MBON→MBON connections in the model based on the neurotransmitter used by each pre-synaptic neuron.

The fly connectome data also show that KCs influence DANs and MBONs via combinations of direct and indirect neural activation and inhibition. Consequently, unlike for MBON→DAN and MBON→MBON connections, we did not constrain the signs of the KC→DAN and KC→MBON connections. This lack of constraint allows the model to capture the net effects of how the KCs influence the DANs and MBONs via direct and indirect interactions; thus, the synaptic weight values in the model for these connections can be thought of as characterizing the effective functional interactions.

In our experiments, olfactory conditioning involved odor pairs with distinct molecular structures, implying that the two odors were likely to be encoded orthogonally. To capture this, the model has 4 KCs, each of which responds to a single odor. The 4 odors included in the model are an attractive CS^{+} odor, an attractive CS^{-} odor, a repulsive CS^{+} odor, and a repulsive CS^{-} odor. Using the subscript i to refer to an individual KC, Eq. (2.1) describes the sparse activation of each KC by its preferred odor input,

$$\tau_{KC,i} \frac{d}{dt} x_{KC,i} = f_{a,KC,i}\left(w_{odor,i}(t)\, x_{odor,i}(t) + b_{KC,i} \right) - x_{KC,i} \quad , \tag{2.1}$$

where $x_{KC,i}(t)$ is the spiking rate of the ith KC, $f_{a,KC,i}$ is the activation function of this KC, $\tau_{KC,i}$ is a time-constant characterizing the rate at which the cell's spiking rate converges to its steady state value, $x_{odor,i}(t)$ equals 1 when the cell's preferred odor is presented to the fly and 0 otherwise, $w_{odor,i}(t)$ is the time-dependent amplitude of neural input signals conveying the presence of odor *i*, and $b_{KC,i}$ is a bias term that sets the cell's baseline spiking rate ($B_{KC,i}$) in the absence of odor *i* and prior to any conditioning, $B_{KC,i} = f_{a,KC,i}\left(b_{KC,i}\right)$.

DANs are activated by electric shocks and receive feedforward, feedback, and cross-module signals from KCs and MBONs (**Fig. 5a**). The MBONs gather signals from the KCs and transmit signals between different MB learning units. For the DANs, we used the subscripts 1, 2, and 3 to refer to PPL1-γ1pedc, -α'2α2, and -α3. For the MBONs, these subscripts refer to each DAN's corresponding MBON, namely MBON-γ1pedc>α/β, -α2sc and -α3. Defining $x_{DAN,j}$ as the spike rate of DAN *j*, $x_{MBON,j}$ as the spike rate of MBON *j,* and $f_{a,DAN,j}$ and $f_{a,MBON,j}$ as activation functions, the dynamics of $x_{DAN,j}$ and $x_{MBON,j}$ are governed by

$$\tau_{DAN,j}\frac{d}{dt}x_{DAN,j} = f_{a,DAN,j}\begin{pmatrix} w_{punish,j}x_{punish}\left(t\right)+\sum_i w_{KD,i,j}\left(x_{KC,i}-B_{KC,i}\right)\ldots \\ +\sum_l w_{MD,l,j}\left(x_{MBON,l}-B_{MBON,l}\right)+b_{DAN,j} \end{pmatrix} - x_{DAN,j} \qquad (2.2)$$

$$\tau_{MBON,j}\frac{d}{dt}x_{MBON,j} = f_{a,MBON,j}\begin{pmatrix} \sum_i w_{KM,i,j}\left(x_{KC,i}-B_{KC,i}\right)\ldots \\ +\sum_l w_{MM,l,j}\left(x_{MBON,l}-B_{MBON,l}\right)+b_{MBON,j} \end{pmatrix} - x_{MBON,j} \quad , \qquad (2.3)$$

where $\tau_{DAN,j}$ and $\tau_{MBON,j}$ are time-constants of spiking adaptation, $w_{KD,i,j}$ is the synaptic weight from KC *i* to DAN *j*, $w_{KM,i,j}$ is the synaptic weight from KC *i* to MBON *j*, $w_{MD,l,j}$ is the synaptic weight from

MBON l to DAN j, $w_{MM,l,j}$ is the synaptic weight from MBON l to MBON j, $B_{KC,i}$, $B_{DAN,j}$ and $B_{MBON,j}$ are baseline spiking rates, and $b_{DAN,j}$ and $b_{MBON,j}$ are bias terms analogous to that in Eq. (2.1).

For the MBONs, the signs of $w_{MD,l,j}$ and $w_{MM,l,j}$ are set according to the MBON neurotransmitters. MBON-γ1pedc>α/β neurotransmission is GABAergic[6]; thus, $w_{MD,1,j}$ and $w_{MM,1,j}$ are set to be non-positive. MBON-α2sc and -α3 signaling are cholinergic[6–8]. Thus, $w_{MD,2,j}$, $w_{MM,2,j}$, $w_{MD,3,j}$ and $w_{MM,3,j}$ are set to be non-negative. We did not set sign constraints on $w_{KD,i,j}$ and $w_{KM,i,j}$ based on KC neurotransmission, because these two weights reflect the net results of direct and indirect neural activation and inhibition. KCs may activate or inhibit DANs through various indirect pathways, such as through lateral horn, anterior paired lateral (APL), and dorsal paired medial neurons[1,2]. One subtype of KCs releases neurotransmitter at the synapses between KCs and MBONs. However, another subtype of KCs also regulates the dynamics of other KCs through the APL neurons[9–11]. The weight $w_{KM,i,j}$ summarizes the net effect of the direct and indirect inputs from KC i to MBON j. Similarly, we also refrained from constraining the sign of $w_{KD,i,j}$ based on the valence of odor i. While it is true that the DANs represent an odor's net valence, DAN activity is shaped by *both* inputs from KCs and feedback from MBONs. These two sources of input could in principle oppose each other. Hence, we left the sign of $w_{KD,i,j}$ unconstrained during parametric fitting of the model to empirical data.

When a fly receives an electric shock punishment, $x_{punish}(t)$ equals 1; otherwise, it equals 0. $w_{punish,j}$ is the weight of punishment signals received by DAN j. Because the spiking rate of PPL1-α′2α2 did not significantly increase during electric shocks (**Fig. 2a,d**), this DAN received no punishment signals in the model. We also found that the change in the spike rate of PPL1-γ1pedc is ~2.44-fold that of PPL1-α3 during the electric shocks (**Fig. 2a,d**). Thus, we manually set $w_{punish,1} / w_{punish,3}$= 2.44, and

$w_{punish,2} = 0$ as input values to the model (**Supplemental Table 2**). Further, since the presentation of different odors with similar innate valences led to comparable levels of DAN activation (**Fig. 2i; Extended Data Fig. 3**), we modeled this empirical result by setting $w_{KD,1,j} = w_{KD,2,j}$ and $w_{KD,3,j} = w_{KD,4,j}$. In other words, the model incorporates the finding that each odor of an attractive odor pair with similar innate valences drives equivalent levels of DAN spiking, as do the two odors of a repulsive odor pair with similar innate valences.

§3. Modeling synaptic plasticity: sensory adaptation and an anti-Hebbian learning rule.

Our model has two types of plasticity in the circuit of KCs, DANs and MBONs (**Fig. 5b**): the adaptation of olfactory sensory neurons and the anti-Hebbian plasticity rules of the KC→MBON synaptic weights. By combining the network architecture articulated in **§2** and the plasticity here in **§3**, we will arrive at a full set of ordinary differential equations that characterize both the firing rate dynamics and the neural plasticity in the model. In **§4** and **§5**, we will simplify these dynamics into a set of recursive equations that we used for parametric fitting of the experimental data. Readers wishing to apply the more general formulation of the model to their own experiments, *e.g.*, for studies with variable CS^+–US intervals, should use the equations developed in **§2** and here in **§3**, where we treat the plasticity dynamics.

When odor is present continuously at a uniform level, the activity levels of olfactory sensory neurons do not stay uniform but instead adapt over time[3] (**Fig. 5b**). The model captures this phenomenon through the time-dependence of $w_{odor,i}(t)$:

$$\frac{d}{dt} w_{odor,i} = -\frac{x_{odor,i}(t)}{\tau_{KC,adapt}} w_{odor,i} + \frac{1 - x_{odor,i}(t)}{\tau_{KC,recover}} \left(A_{odor,i} - w_{odor,i} \right) \quad , \qquad (3.1)$$

where $A_{odor,i}$ denotes the amplitude of odor *i*, and $\tau_{KC,adapt}$ and $\tau_{KC,recover}$ are time-constants that respectively characterize the rates of adaptation in the presence of odor and following the offset of odor presentation.

Based on the biological finding that concurrent activation of a DAN and KCs leads to plasticity of the odor-evoked spiking responses by the DAN's corresponding MBON[4,5], in our model the concurrent activation of KC *i* and DAN *j* modifies the weight of the KC *i* to MBON *j* synapse, $w_{KM,i,j}$, according to an anti-Hebbian rule (**Extended Data Fig. 10b**). If the fly receives a punishment following odor presentation (a forward pairing), $w_{KM,i,j}$ decreases, whereas $w_{KM,i,j}$ increases if the punishment occurs before odor presentation (a backward pairing) (**Extended Data Fig. 10b**). We modeled these effects as follows[12]:

$$\frac{d}{dt}y_{KC,i} = k_{KC}\left(x_{KC,i} - B_{KC,i}\right) - \gamma_{KC}y_{KC,i} \tag{3.2}$$

$$\frac{d}{dt}y_{DAN,j} = k_{DAN}\left(x_{DAN,j} - B_{DAN,j}\right) - \gamma_{DAN}y_{DAN,j} \tag{3.3}$$

$$\frac{d}{dt}u_{KM,i,j} = \left[\left(x_{KC,i} - B_{KC,i}\right)y_{DAN,j} - \left(x_{DAN,j} - B_{DAN,j}\right)y_{KC,i}\right] - \frac{u_{KM,i,j}}{\tau_{u,j}} \tag{3.4}$$

$$\tau_{w,j}\frac{d}{dt}w_{KM,i,j} = u_{KM,i,j} - w_{KM,i,j} \quad . \tag{3.5}$$

In Eqs. (3.2) and (3.3), $y_{KC,i}$ and $y_{DAN,j}$ are low-pass filtered versions of the spiking signals conveyed to the MBON by KC *i* and DAN *j*. Unlike past modeling studies[12] but in accord with prior experimental findings[4,5], our model has distinct amplitudes (k_{KC} and k_{DAN}) and time-constants (γ_{KC} and γ_{DAN}) characterizing the plasticity that arises from forward *vs.* backward pairings. In Eq. (3.4), $\tau_{u,j}$ is a plasticity time constant for MBON *j*. $w_{KM,i,j}$ is the synaptic weight between KC *i* and MBON *j*. $u_{KM,i,j}$ is an intermediate variable used to calculate $w_{KM,i,j}$ and $\tau_{w,j}$ is the time-constant of the low-pass filter in Eq. (3.5), which ensures that the synaptic weight $w_{KM,i,j}$ is a temporally low-pass filtered version of $u_{KM,i,j}$. This prevents rapid fluctuations of the synaptic weights in the model, which might have occurred

if we had directly used $u_{KM,i,j}$ as the synaptic weight between KC *i* and MBON *j*.

The KC→MBON-γ1pedc>α/β synaptic connections store short-term memories, and the KC→MBON-α2sc and KC→MBON-α3 connections store long-term memories[5,13,14]. In our experiments, learning-induced plasticity in MBON-γ1pedc>α/β persisted for <1 hr (**Fig. 3f** and **Extended Data Fig. 7a**), whereas MBON-α3 plasticity lasted >24 hrs (**Fig. 4a–d**). Our modeling efforts accounted for this difference between short- and long-term memory in two ways.

First, we assigned distinct time-constants for the weights of the KC→MBON-γ1pedc>α/β synapse ($\tau_{u,1}$) and those of the KC→MBON-α2sc and -α3 synapses ($\tau_{u,2}$ and $\tau_{u,3}$). Parametric fits of the model to the spiking data confirmed that the KC→MBON-γ1pedc>α/β synaptic time-constant is much briefer than the time-constants of the KC→MBON-α2sc and KC→MBON-α3 connections (**Supplementary Table 3**).

Second, to allow for the possibility of long-term memory consolidation processes, we let the model use two different sets of values for the time-constants, $\tau_{u,2}$ and $\tau_{u,3}$. When we parametrically fit the model to the experimental spiking data, one pair of values for $\tau_{u,2}$ and $\tau_{u,3}$ was used for data collected in the first 3 hours after associative conditioning; these time-constant values were relatively brief. Another set of values for $\tau_{u,2}$ and $\tau_{u,3}$ was used for data collected at time points after 3 hours; these values for $\tau_{u,2}$ and $\tau_{u,3}$ were considerably longer (**Supplementary Table 3**). The rationale for implementing this switch in the values of $\tau_{u,2}$ and $\tau_{u,3}$ was the key finding that memory decay in the first 3 hours after conditioning is much faster than that afterward (**Fig. 4c,d**). Notably, we were unable to fit the empirical data by modeling memory decay with a single decaying exponential function; this inability led us to introduce the second set of time-constant values, which in actuality could arise from a bi-exponential memory decay process[15–17].

§4. Mathematical simplifications of the neural network dynamics.

We simulated the differential equations governing the neuronal firing rate (2.1–2.3) and plasticity (3.1–3.5) dynamics using the MATLAB (Mathworks Inc.) function ode15s(), which solves differential equations numerically. This approach was time-consuming and required ~14 s to provide results for a single, fixed set of parameter values. To accelerate the simulations and analyze key features of the neural network across many different sets of parameter values, we simplified the model by applying several approximations regarding the spiking dynamics (**§4**) and plasticity processes (**§5**).

Here, in **§4**, we simplify equations 2.1–2.3 into recursive equations. We also simplify the activation functions of the KCs, DANs, and MBONs based on the following three observations. (1) In our experiments, we set the odorant concentrations within ranges that do not saturate the flies' odorant receptors (**Extended Data Fig. 3**). (2) Our voltage imaging studies of DANs showed that the spiking rate changes triggered by an odor and shock presented jointly approximately equal the sum of the spiking rate changes triggered by the individual stimuli (**Fig. 4e–h**). This indicates that DANs operate approximately in their activation functions' linear range. This is a key property of our model and implies that DANs linearly integrate the valences of jointly presented stimuli. (3) Our experiments showed that, unlike for KCs and DANs, the spiking rates of MBONs can attain upper and lower bounds (**Fig. 4a–d**; **Extended Data Figs. 4,6**). Hence, while we approximated the activation functions of KCs and DANs as linear functions, this approximation would be incorrect for MBONs, for which we retain baseline and maximum firing rates in the model (**Supplementary Table 2**). Further, after transforming the model equations from a continuous time formulation to a recursive version with discrete time steps, at each time step we describe each cell's activity using mean values of its spike rate, time-averaged across each individual training, testing, or resting bout.

First, we approximated the activation functions of KCs and DANs as linear functions. This

approximation allows us to cancel the baseline firing rates of KCs and DANs from their respective dynamical equations and reduces the number of parameters needed to fit the model to data. In multiple of this paper's figures and extended data figures, we plotted changes in neural spiking rates from baseline levels prior to conditioning:

$$\Delta x_{KC,i} = x_{KC,i} - B_{KC,i} \tag{4.1}$$

$$\Delta x_{DAN,j} = x_{DAN,j} - B_{DAN,j} \tag{4.2}$$

$$\Delta x_{MBON,l} = x_{MBON,l} - B_{MBON,l} \quad . \tag{4.3}$$

In our studies, we did not use extremely high concentrations of odorants, which might have saturated the flies' odorant receptors (**Extended Data Fig. 3**). Thus, we approximated KC activation functions as linear, $f_{a,KC,i}(x) = x$. Using this approximation and combining equations (2.1) and (4.1) yields:

$$\tau_{KC,i} \frac{d}{dt} \Delta x_{KC,i} = w_{odor,i}(t) x_{odor,i}(t) - \Delta x_{KC,i} \quad . \tag{4.4}$$

In our imaging studies of DANs, we found that they linearly integrate the valences of jointly presented stimuli, such that spiking rate changes triggered by an odor and shock presented jointly approximately equal the sum of the spiking rate changes triggered by the individual stimuli (**Fig. 4e–h**). Thus, DANs operate approximately in the linear range of their activation function, *i.e.,* $f_{a,DAN,j}(x) = x$, implying that Eq. (2.2) can be simplified as follows:

$$\tau_{DAN,j} \frac{d}{dt} \Delta x_{DAN,j} = w_{punish,j} x_{punish}(t) + \sum_i w_{KD,i,j} \Delta x_{KC,i} + \sum_l w_{MD,l,j} \Delta x_{MBON,l} - \Delta x_{DAN,j} \quad . \tag{4.5}$$

Unlike our results from DANs, we observed experimentally that MBON spiking rates could attain upper and lower bounds (**Fig. 4a–d**; **Extended Data Figs. 4,6**). Thus, we did not approximate MBON activation functions as linear. Instead, we used piecewise linear functions:

$$f_{a,MBON,j}\left(x\right)=\begin{cases}0 & x<0\\ x & 0\le x<M_{MBON,j}\\ M_{MBON,j} & x\ge M_{MBON,j}\end{cases}\quad , \tag{4.6}$$

where $M_{MBON,j}$ is the maximum spiking rate of MBON *j*. Based on our empirically measured values, we set $M_{MBON,1}$ = 71.6 s^{-1}, $M_{MBON,2}$ = 17.9 s^{-1}, $M_{MBON,3}$ = 31.2 s^{-1}, $B_{MBON,1}$ = 35.2 s^{-1}, $B_{MBON,2}$ = 9.0 s^{-1}, and $B_{MBON,3}$ = 11.25 s^{-1} (**Extended Data Figs. 4,6**; **Supplementary Table 2**). Plainly, the baseline spiking rates of MBONs do not reach the upper and lower limits. Thus, we concluded that $b_{MBON,j}=B_{MBON,j}$. Combining (2.3), (4.1) and (4.3) yields (4.7), which describes the spike rate changes:

$$\tau_{MBON,j}\frac{d}{dt}\Delta x_{MBON,j}=f_{a,MBON,j}\left(\begin{array}{l}\sum_i w_{KM,i,j}\Delta x_{KC,i}\\ +\sum_l w_{MM,l,j}\Delta x_{MBON,l}+B_{MBON,j}\end{array}\right)-B_{MBON,j}-\Delta x_{MBON,j}\quad . \tag{4.7}$$

The time-constants $\tau_{KC,i}$, $\tau_{DAN,j}$ and $\tau_{MBON,j}$ are sufficiently brief (~10 ms) that the dynamics of Eqs. (4.4) and (4.5) quickly reach steady-state values when the input values of $x_{odor,i}\left(t\right)$ and $x_{punish}\left(t\right)$ change. This allows us to simplify (4.4), (4.5), and (4.7) as follows:

$$\Delta x_{KC,i}=w_{odor,i}\left(t\right)x_{odor,i}\left(t\right) \tag{4.8}$$

$$\Delta x_{DAN,j}=w_{punish,j}x_{punish}\left(t\right)+\sum_i w_{KD,i,j}\Delta x_{KC,i}+\sum_l w_{MD,l,j}\Delta x_{MBON,l} \tag{4.9}$$

$$\Delta x_{MBON,j}=f_{a,MBON,j}\left(\sum_i w_{KM,i,j}\Delta x_{KC,i}+\sum_l w_{MM,l,j}\Delta x_{MBON,l}+B_{MBON,j}\right)-B_{MBON,j}\quad . \tag{4.10}$$

During resting bouts, $x_{odor,i}\left(t\right)$ = 0 and $x_{punish}\left(t\right)$ = 0. From Eqs. (4.8), (4.9), and (4.10), one finds that $\Delta x_{KC,i}$, $\Delta x_{DAN,j}$ and $\Delta x_{MBON,j}$ are all zero during resting bouts. During training, testing and imaging bouts, at least one of $x_{odor,i}\left(t\right)$ and $x_{punish}\left(t\right)$ is non-zero, implying that $\Delta x_{KC,i}$, $\Delta x_{DAN,j}$ and $\Delta x_{MBON,j}$ may be non-zero. Since the durations of training, testing and imaging bouts are much less than

the rest intervals between them (**Figs. 3a, 3d, 4k** and **5j,k**), we focused on the set of discrete time points, $\{t_k\}$, corresponding to each training, testing, and imaging bout, and we used a single, time-averaged mean spiking rate to represent each cell's activity level at each time point. With this approach, Eqs. (4.8), (4.9) and (4.10) become:

$$\overline{\Delta x_{KC,i}(t_k)} = \overline{w_{odor,i}(t_k)} x_{odor,i}(t_k) \tag{4.11}$$

$$\overline{\Delta x_{DAN,j}(t_k)} = w_{punish,j} x_{punish}(t_k) + \sum_i w_{KD,i,j} \overline{\Delta x_{KC,i}(t_k)} + \sum_l w_{MD,l,j} \overline{\Delta x_{MBON,l}(t_k)} \tag{4.12}$$

$$\overline{\Delta x_{MBON,j}(t_k)} = f_{a,MBON,j}\left(\begin{array}{l} \sum_i \overline{w_{KM,i,j}(t_k)} \overline{\Delta x_{KC,i}(t_k)} \\ + \sum_l w_{MM,l,j} \overline{\Delta x_{MBON,l}(t_k)} + B_{MBON,j} \end{array} \right) - B_{MBON,j} \quad , \tag{4.13}$$

where, for any variable $a(t)$, the notation $\overline{a(t_k)}$ denotes the mean value of $a(t)$, time-averaged over the kth training, testing, or imaging bout, *i.e.,* at time-point t_k. Hence, $\overline{\Delta x_{KC,i}(t_k)}$, $\overline{\Delta x_{DAN,j}(t_k)}$ and $\overline{\Delta x_{MBON,j}(t_k)}$ denote mean spike rates for the kth bout. $\overline{w_{odor,i}(t_k)}$ is the mean weight of odor *i* on KC *i*, and $\overline{w_{KM,i,j}(t_k)}$ is the mean connection weight from KC *i* to MBON *j* at the kth bout.

§5. Mathematical simplifications of the synaptic plasticity dynamics.

Having simplified the neural dynamics and interactions into a coupled set of recursive equations in **§4**, in this section we reduce the plasticity dynamics to a set of recursive equations. The plasticity rule derived here in **§5** shows how the linear integration of innate and learnt valences by the DANs shapes plasticity in the long-term (α3) memory module. Altogether, the use of the combined set of recursive equations developed in **§4** and **§5** accelerated our simulations of the model, facilitated fits to experimental data, and yielded a clearer understanding of the model dynamics.

To simplify the model's implementation of KC→MBON synaptic plasticity, we exploited the

linear dependence of the anti-Hebbian plasticity rule as a function of its input levels of neural spiking, the finding that DANs linearly integrate the valences of jointly presented stimuli (**Fig. 4e–h**), and the observation that odors' innate and learnt valences concurrently activate the DANs and KCs (**Fig. 5b**). These observations imply that, during associative conditioning, the plasticity induced at a KC→MBON connection is the linear sum of the plasticity that would be induced by the US valence and the innate and learnt odor valences, if these were hypothetically presented individually to the fly in conjunction with odor delivery (**Extended Data Fig. 10a**). The innate and learnt odor valences are signaled concurrently by the DAN and hence use a shared weighting parameter in the computation of plasticity induction. Since our experiments used a fixed value (Δt_{punish}= 3 s) for the CS$^+$–US interval, the weight of the US on plasticity induction in our model is determined by a single fitted parameter value.

In equations (4.11–4.13) above, $w_{odor,i}$ and $w_{KM,i,j}$ change with time, due to sensory adaptation and anti-Hebbian plasticity, respectively, whereas the other synaptic weights remain constant. First, we sought recursive equations from which we could calculate the mean weight, $w_{odor,i}$, of the odor input:

$$w_{odor,i}\left(t_1, start\right) = A_{odor,i} \tag{5.1}$$

$$w_{odor,i}\left(t_k, end\right) = \begin{cases} w_{odor,i}\left(t_k, start\right) e^{-t_{on,k}/\tau_{KC,adapt}} & x_{odor,i}\left(t_k\right) = 1 \\ A_{odor,i} - \left(A_{odor,i} - w_{odor,i}\left(t_k, start\right)\right) e^{-t_{on,k}/\tau_{KC,recover}} & x_{odor,i}\left(t_k\right) = 0 \end{cases} \tag{5.2}$$

$$w_{odor,i}\left(t_{k+1}, start\right) = A_{odor,i} - \left(A_{odor,i} - w_{odor,i}\left(t_k, end\right)\right) e^{-t_{off,k}/\tau_{KC,recover}} \tag{5.3}$$

$$\overline{w_{odor,i}\left(t_k\right)} \approx \left[w_{odor,i}\left(t_k, start\right) + w_{odor,i}\left(t_k, end\right)\right] / 2 \quad , \tag{5.4}$$

where $w_{odor,i}\left(t_k, start\right)$ and $w_{odor,i}\left(t_k, end\right)$ are the weights with which odor *i* activates KC *i* at the starting and ending time-points of the kth training, testing or imaging bout. $t_{on,k}$ is the duration of the kth

such bout. $t_{off,k}$ is the duration of the rest interval after the kth experimental bout. Since $A_{odor,i}$ can be absorbed into $w_{odor,i}$, we set $A_{odor,i}$ = 1. We set $\tau_{KC,adapt}$ = 20 s based on past results from fly olfactory receptor neurons[3] (**Supplementary Table 2**) and determined the value of $\tau_{KC,recover}$ via our fits to data (**Supplementary Table 3**).

We used the changes in the time-averaged KC and DAN spike rates to calculate the changes in the KC→MBON synaptic weights. Since the durations of training, testing and imaging bouts are much less than the time constant, $\tau_{u,j}$, we approximated $u_{KM,i,j}$ as follows:

$$u_{KM,i,j}\left(t_k,end\right) \approx \int_0^{t_{on,k}} \left(\Delta x_{KC,i} y_{DAN,j} - \Delta x_{DAN,j} y_{KC,i}\right) dt + u_{KM,i,j}\left(t_k,start\right) \tag{5.5}$$

$$u_{KM,i,j}\left(t_{k+1},start\right) \approx u_{KM,i,j}\left(t_k,end\right) e^{-t_{off,k}/\tau_{u,j}} \quad , \tag{5.6}$$

where $u_{KM,i,j}\left(t_k,start\right)$ and $u_{KM,i,j}\left(t_k,end\right)$ are the values of $u_{KM,i,j}\left(t\right)$ at the starting and ending time-points of the kth training, testing or imaging bout.

Because the time constant $\tau_{w,j}$ is much greater than $t_{on,k}$ but much smaller than $t_{off,k}$, the weight $w_{KM,i,j}$ does not significantly change during the kth experimental bout but instead converges to $u_{KM,i,j}$ at the end of the subsequent resting bout:

$$\overline{w_{KM,i,j}\left(t_k\right)} \approx u_{KM,i,j}\left(t_k,start\right) \quad . \tag{5.7}$$

By combining Eqs. (5.5–5.7), we derived the recursion formula Eq. (5.8):

$$\overline{w_{KM,i,j}\left(t_{k+1}\right)} \approx \left(\overline{\Delta w_{KM,i,j}\left(t_k\right)} + \overline{w_{KM,i,j}\left(t_k\right)}\right) e^{-t_{off}/\tau_{u,j}} \quad , \tag{5.8}$$

where $\overline{\Delta w_{KM,i,j}\left(t_k\right)}$ is the change of the time-averaged weight from KC i to MBON j at the kth experimental bout:

$$\overline{\Delta w_{KM,i,j}\left(t_k\right)} = \int_0^{t_{on,k}} \left(\Delta x_{KC,i} y_{DAN,j} - \Delta x_{DAN,j} y_{KC,i}\right) dt \quad . \tag{5.9}$$

The initial value of the formula Eq. (5.8) is the synaptic weight from KC i to MBON j at the start of our experiments. Before our experiments, flies had no preference between the CS$^+$ and CS$^-$ odors. Therefore, we set the initial values of synaptic weights from all KCs to MBON j to be the same:

$$\overline{w_{KM,i,j}\left(t_1\right)} = w_{KM,initial,j} \quad . \tag{5.10}$$

To perform the integration in Eq. (5.9), we derived the analytic solution of Eqs. (3.2) and (3.3), and defined a functional ΔW:

$$y_{KC}\left(t\right) = k_{KC} e^{-\gamma_{KC} t} \int_0^t e^{\gamma_{KC} t_1} x_{KC}\left(t_1\right) dt_1 \tag{5.11}$$

$$y_{DAN}\left(t\right) = k_{DAN} e^{-\gamma_{DAN} t} \int_0^t e^{\gamma_{DAN} t_1} x_{DAN}\left(t_1\right) dt_1 \tag{5.12}$$

$$\begin{aligned} &\Delta W\left(\Delta x_{KC}\left(t\right), \Delta x_{DAN}\left(t\right)\right) = \int_0^{t_{on}} \left(\Delta x_{KC}\left(t\right) y_{DAN}\left(t\right) - \Delta x_{DAN}\left(t\right) y_{KC}\left(t\right)\right) dt \\ &= \int_0^{t_{on}} \left(\Delta x_{KC}\left(t\right) k_{DAN} e^{-\gamma_{DAN} t} \int_0^t e^{\gamma_{DAN} t_1} x_{DAN}\left(t_1\right) dt_1 - \Delta x_{DAN}\left(t\right) k_{KC} e^{-\gamma_{KC} t} \int_0^t e^{\gamma_{KC} t_1} x_{KC}\left(t_1\right) dt_1\right) dt \end{aligned} \quad . \tag{5.13}$$

The $\overline{\Delta w_{KM,i,j}\left(t_k\right)}$ defined in Eq. (5.9) equals $\Delta W\left(\Delta x_{KC,i}\left(t\right), \Delta x_{DAN,j}\left(t\right)\right)$. The ΔW defined by Eq. (5.13) is a linear functional of the two input functions $\Delta x_{KC}\left(t\right)$ and $\Delta x_{DAN}\left(t\right)$. Eq. (4.9) implies that the spike rate of each DAN is a linear combination of the punishment-related (US) input and the spike rates of KC and MBON inputs to the DAN. Thus, the changes in the KC→MBON synaptic weights are linear sums of the contributions from the punishment and the spike rates of KCs and MBONs:

$$\begin{aligned} &\Delta W\left(\Delta x_{KC,i}\left(t\right), \Delta x_{DAN,j}\left(t\right)\right) \\ &= \Delta W\left(\Delta x_{KC,i}\left(t\right), w_{punish,j} x_{punish}\left(t\right) + \sum_{i'} w_{KD,i',j} \Delta x_{KC,i'}\left(t\right) + \sum_{l} w_{MD,l,j} \Delta x_{MBON,l}\left(t\right)\right) \\ &= w_{punish,j} \Delta W\left(\Delta x_{KC,i}\left(t\right), x_{punish}\left(t\right)\right) + \sum_{i'} w_{KD,i',j} \Delta W\left(\Delta x_{KC,i}\left(t\right), \Delta x_{KC,i'}\left(t\right)\right) \\ &+ \sum_{l} w_{MD,l,j} \Delta W\left(\Delta x_{KC,i}\left(t\right), \Delta x_{MBON,l}\left(t\right)\right) \end{aligned} \quad . \tag{5.14}$$

Because in each training or imaging bout, we perfuse only one kind of odor, when $\Delta x_{KC,i}\left(t\right) \neq 0$,

$\Delta x_{KC,i'}(t)=0$ if $i'\neq i$. Thus, the term $\sum_{i'} w_{KD,i',j}\Delta W\left(\Delta x_{KC,i}(t),\Delta x_{KC,i'}(t)\right)$ can be simplified into $w_{KD,i,j}\Delta W\left(\Delta x_{KC,i}(t),\Delta x_{KC,i}(t)\right)$. To calculate the terms $\Delta W\left(\Delta x_{KC,i}(t),x_{punish}(t)\right)$, $\Delta W\left(\Delta x_{KC,i}(t),\Delta x_{KC,i}(t)\right)$, and $\Delta W\left(\Delta x_{KC,i}(t),\Delta x_{MBON,l}(t)\right)$ from the mean spike rates of KCs, DANs and MBONs, we first defined two square-wave functions [Eqs. (5.15) and (5.16)] to describe the time-dependence of CS and US presentation:

$$\Delta x_{KC}(t)=\begin{cases}\overline{\Delta x_{KC}} & 0<t\leq\tau\\ 0 & otherwise\end{cases} \tag{5.15}$$

$$\Delta x_{DAN}(t)=\begin{cases}\overline{\Delta x_{DAN}} & \Delta t<t\leq\Delta t+\tau\\ 0 & otherwise\end{cases}, \tag{5.16}$$

where $\overline{\Delta x_{KC}}$ and $\overline{\Delta x_{DAN}}$ are constants that set the square-wave amplitudes, Δt is the time interval between KC and DAN activity, and τ is the duration of odor or punishment delivery. (In our experiments, odor and shock delivery were of equal durations). We then inserted (5.15) and (5.16) into Eq. (5.13), used the linear dependence of ΔW on its inputs, and defined the anti-Hebbian amplitude function $A_{AH}(\Delta t)$ as follows in Eq. (5.17):

$$A_{AH}(\Delta t)=\frac{\Delta W\left(\Delta x_{KC}(t),\Delta x_{DAN}(t)\right)}{\overline{\Delta x_{KC}}\,\overline{\Delta x_{DAN}}}$$
$$=\begin{cases}\frac{k_{DAN}}{\gamma_{DAN}^2}\left(1-e^{-\gamma_{DAN}\tau}\right)^2 e^{-\gamma_{DAN}(-\Delta t-\tau)} & \Delta t\leq-\tau\\ \left(\frac{k_{DAN}}{\gamma_{DAN}}-\frac{k_{KC}}{\gamma_{KC}}\right)(\Delta t+\tau)+\frac{k_{DAN}}{\gamma_{DAN}^2}\left(e^{-\gamma_{DAN}(\tau-\Delta t)}-2e^{-\gamma_{DAN}(-\Delta t)}+1\right)-\frac{k_{KC}}{\gamma_{KC}^2}\left(e^{-\gamma_{KC}(\Delta t+\tau)}-1\right) & -\tau<\Delta t\leq 0,\\ \left(\frac{k_{DAN}}{\gamma_{DAN}}-\frac{k_{KC}}{\gamma_{KC}}\right)(\tau-\Delta t)+\frac{k_{DAN}}{\gamma_{DAN}^2}\left(e^{-\gamma_{DAN}(\tau-\Delta t)}-1\right)-\frac{k_{KC}}{\gamma_{KC}^2}\left(e^{-\gamma_{KC}(\Delta t+\tau)}-2e^{-\gamma_{KC}\Delta t}+1\right) & 0<\Delta t\leq\tau\\ -\frac{k_{KC}}{\gamma_{KC}^2}\left(1-e^{-\gamma_{KC}\tau}\right)^2 e^{-\gamma_{KC}(\Delta t-\tau)} & \Delta t>\tau\ .\end{cases} \tag{5.17}$$

Given the fixed value of Δt_{punish}= 3 s (**Extended Data Fig. 10b**), a single parameter value, $A_{AH}\left(\Delta t_{punish}\right)$, characterizes the joint influence of the CS and US on the plasticity of the KC→MBON synapses. In addition to the US, the odor used for conditioning also has a valence and drives DAN activity, which is shaped by the concurrent odor-evoked inputs from KCs and MBONs (**Fig. 5a,b**; Eq. (5.14)). The influence of this concurrent odor-evoked KC and DAN activity on plasticity of the KC→MBON synapse can also be characterized by a single parameter value, $A_{AH}\left(0\right)$. We thereby achieved Eq. (5.18) using (5.8) and (5.14):

$$\overline{\Delta w_{KM,i,j}\left(t_k\right)} = \overline{\Delta x_{KC,i}\left(t_k\right)} \left[\begin{array}{l} A_{AH}\left(\Delta t_{punish}\right) w_{punish,j} x_{punish}\left(t_k\right) \\ + A_{AH}\left(0\right) \left(w_{KD,i,j} \overline{\Delta x_{KC,i}\left(t_k\right)} + \sum_l w_{MD,l,j} \overline{\Delta x_{MBON,l}\left(t_k\right)} \right) \end{array} \right] . \quad (5.18)$$

Overall, we see that owing to the fixed value of Δt_{punish} = 3 s in our studies, the effects of the parameters k_{CS}, k_{US}, γ_{CS} and γ_{US} in Eqs. (3.2) and (3.3) can be summarized with just two parameters, $A_{AH}\left(\Delta t_{punish}\right)$ and $A_{AH}\left(0\right)$. The values of these latter two parameter were determined directly through fits to experimental data (see **§7**).

Notably, (5.18) can be more easily understood by converting it into a form that highlights the role of odor valence on the change in the KC→MBON synaptic weights. Here, we define the component of firing by DAN *j* that is triggered by the net valence of odor *i*:

$$\overline{v_{i,j}\left(t_k\right)} = w_{KD,i,j} \overline{\Delta x_{KC,i}\left(t_k\right)} + \sum_l w_{MD,l,j} \overline{\Delta x_{MBON,l}\left(t_k\right)} \quad . \quad (5.19)$$

The first term of (5.19) is shaped purely by the innate odor valence, because the KC→DAN weight, $w_{KD,i,j}$, does not change during training. The second term of Eq. (5.19) is influenced by both the innate and learnt valences, since the KC→MBON weight, $w_{KM,i,j}$, changes from its initial value during

training and causes $\overline{\Delta x_{MBON,l}\left(t_k\right)}$ to differ between the CS$^+$ and CS$^-$ odors. Using (5.19), Eq. (5.18) simplifies into (5.20), which describes plasticity at the KC→MBON synapse in the models' recursive formulation:

$$\overline{\Delta w_{KM,i,j}\left(t_k\right)} = \overline{\Delta x_{KC,i}\left(t_k\right)}\left[A_{AH}\left(\Delta t_{punish}\right)w_{punish,j}x_{punish}\left(t_k\right) + A_{AH}\left(0\right)\overline{v_{i,j}\left(t_k\right)}\right] \quad . \qquad (5.20)$$

In **Extended Data Fig. 10a**, we plot Eq. (5.20) with $\overline{v_{i,j}\left(t_k\right)}$ on the *x*-axis and $\overline{\Delta w_{KM,i,j}\left(t_k\right)}$ on the *y*-axis (in units of $\overline{\Delta x_{KC,i}\left(t_k\right)}$) for different DANs, either with or without electric shock. The plots are linear with a slope of $A_{AH}\left(0\right)$ and *y*-intercepts of $A_{AH}\left(\Delta t_{punish}\right)w_{punish,j}x_{punish}\left(t_k\right)$. The plots are distinct for different DANs, because $w_{punish,j}$ differs between DANs.

Altogether, through the above approximations we achieved a complete set of recursive equations [Eqs. (4.11), (4.12), (4.13), (5.1), (5.2), (5.3), (5.4), (5.8) and (5.18)]. The time needed to simulate the model with this equation set and one set of parameter values was ~0.02 s, about ~700-fold faster than simulating the full set of differential equations, (2.1–2.3) and (3.1–3.5). We used the recursive formulation for fits of the experimental data to two different versions of the model, one with three memory modules (γ1, α2, and α3 compartments; **Fig. 5a**) and one with just two modules (γ1 and α3; **Extended Data Fig. 10i**). Results from these fits are discussed below in **§7** and **§8**.

§6. Theoretical analysis of the recurrent circuitry connecting DANs and MBONs.

In this section, we show why it was important to represent the MBON activation functions as nonlinear (piecewise linear) functions and to include the baseline and maximum MBON spike rates in the set of model parameters (**Supplementary Table 2**). This was not the case for KCs and DANs, for which we used linear activation functions.

The main nonlinearity in the model indeed arises via the piecewise linear MBON activation function in Eq. (4.6). If the MBON spike rates are between their lower and upper bounds, we find from Eq. (4.7)

$$\overline{\Delta\vec{x}_{MBON}\left(t_k\right)} = \sum_i \overline{\vec{w}_{KM,i}\left(t_k\right)}\,\overline{\Delta x_{KC,i}\left(t_k\right)} + W_{MM}^T\,\overline{\Delta\vec{x}_{MBON}\left(t_k\right)} \quad , \tag{6.1}$$

where $\overline{\Delta\vec{x}_{MBON}\left(t_k\right)}$ is a column vector whose j^{th} element is $\overline{\Delta x_{MBON,j}\left(t_k\right)}$, $\overline{\vec{w}_{KM,i}\left(t_k\right)}$ is a column vector whose j^{th} element is $\overline{w_{KM,i,j}\left(t_k\right)}$, and W_{MM} is a matrix whose element in row i and column j is $w_{MM,i,j}$. The reason for using these vector and matrix forms is that they allow us to simplify the format of (5.18) through the use of the expression for the inverse matrix, $\left(I - W_{MM}\right)^{-1}$:

$$\begin{aligned}\overline{\Delta\vec{w}_{KM,i}\left(t_k\right)} &= \overline{\Delta x_{KC,i}\left(t_k\right)} A_{AH}\left(\Delta t_{punish}\right)\vec{w}_{punish}x_{punish}\left(t_k\right) + \left[\overline{\Delta x_{KC,i}\left(t_k\right)}\right]^2 A_{AH}\left(0\right)\vec{w}_{KD,i} \\ &+ \left[\overline{\Delta x_{KC,i}\left(t_k\right)}\right]^2 A_{AH}\left(0\right)W_{MD}^T\left(I - W_{MM}^T\right)^{-1}\overline{\vec{w}_{KM,i}\left(t_k\right)}\end{aligned} \quad . \tag{6.2}$$

Here, $\overline{\Delta\vec{w}_{KM,i}\left(t_k\right)}$ is a column vector whose j^{th} element is $\overline{\Delta w_{KM,i,j}\left(t_k\right)}$, $\vec{w}_{punish}$ is a column vector whose j^{th} element is $w_{punish,j}$, and W_{MD} is a weight matrix whose element in row i and column j is $w_{MD,i,j}$. To identify the temporal stability or instability (6.2), we must determine whether the matrix in front of $\overline{\vec{w}_{KM,i}\left(t_k\right)}$ has a positive eigenvalue or not. This matrix is:

$$\begin{aligned}&\left[\overline{\Delta x_{KC,i}\left(t_k\right)}\right]^2 A_{AH}\left(0\right)W_{MD}^T\left(I - W_{MM}^T\right)^{-1} \\ &= \left[\overline{\Delta x_{KC,i}\left(t_k\right)}\right]^2 A_{AH}\left(0\right)\begin{pmatrix} w_{MD,1,1} & 0 & 0 \\ w_{MD,1,2} & w_{MD,2,2} & 0 \\ w_{MD,1,3} & w_{MD,2,3} & w_{MD,3,3}\end{pmatrix}\begin{pmatrix} 1 & 0 & 0 \\ -w_{MM,1,2} & 1 & 0 \\ -w_{MM,1,3} & 0 & 1\end{pmatrix}^{-1} \\ &= \left[\overline{\Delta x_{KC,i}\left(t_k\right)}\right]^2 A_{AH}\left(0\right)\begin{pmatrix} w_{MD,1,1} & 0 & 0 \\ w_{MD,1,2} + w_{MM,1,2}w_{MD,2,2} & w_{MD,2,2} & 0 \\ w_{MD,1,3} + w_{MM,1,2}w_{MD,2,3} + w_{MM,1,3}w_{MD,3,3} & w_{MD,2,3} & w_{MD,3,3}\end{pmatrix}\end{aligned} \quad . \tag{6.3}$$

We note that $\left[\overline{\Delta x_{KC,i}\left(t_k\right)}\right]^2$ is non-negative, $A_{AH}\left(0\right)$ is negative owing to the anti-Hebbian plasticity rule, and $w_{MD,1,1}$ is negative owing to the GABAergic transmission at the MBON-γ1→PPL1-γ1 synapse[6]. Thus, at least one eigenvalue of the matrix, $\left[\overline{\Delta x_{KC,i}\left(t_k\right)}\right]^2 A_{AH}\left(0\right) w_{MD,1,1}$, is non-negative. This implies that the connection from MBON-γ1pedc>α/β to PPL1-γ1pedc constitutes a positive feedback loop that steadily increases the weight of the KC→MBON-γ1 synapse. Further, we can again use the known neurotransmitter identities within the MB circuitry[6–8] (**Supplementary Table 3**; **Fig 5a**) to conclude that $w_{MD,1,2} + w_{MM,1,2} w_{MD,2,2}$ and $w_{MD,1,3} + w_{MM,1,2} w_{MD,2,3} + w_{MM,1,3} w_{MD,3,3}$ are also negative. Therefore, the feedback connections from MBON-γ1pedc>α/β to PPL1-α'2α2 and -α3 have the net effect of increasing KC→MBON synaptic weights and memory formation in the MB α-lobe.

Crucially, however, this positive feedback does not lead to a divergence of the weight values. When the absolute values of the KC→MBON weights are very large, MBON spike rates reach their lower bounds, and activity in the circuit's feedback and feedforward pathways becomes saturated, precluding any further increases in DAN activity (**Extended Data Fig. 11**). This crucial prevention of divergence in the model stems directly from the piecewise linear form of the MBON activation function.

§7. Estimating the parameter values of the model.

We fit the experimental data (**Figs. 2i, 3e, 3f, 4c, 4d, 5c; Extended Data Figs. 8b,c** and **10c,d,j**) using the recursive model equations to estimate values for the set of model parameters, $\boldsymbol{\theta}$. We assumed that the measured spike rates of neuron *i* at time point *k* were drawn from independent normal distributions, $N\left(\mu_{i,k}\left(\boldsymbol{\theta}\right), \sigma_{i,k}^2\right)$. Thus, the log-likelihood of the empirical data given a set of parameter values, $\boldsymbol{\theta}$, is:

$$\ln \mathrm{L}\left(\boldsymbol{\theta}\right) = -\sum_{i,k}\left(\ln \sigma_{i,k} + \frac{1}{2}\ln 2\pi\right) - \frac{1}{2}\sum_{i,k}\left(\frac{x_{i,k} - \mu_{i,k}\left(\boldsymbol{\theta}\right)}{\sigma_{i,k}}\right)^2 \quad , \tag{7.1}$$

where, $x_{i,k}$ and $\sigma_{i,k}$ are the mean and SEM values of the spike rate of neuron *i* at time point *k,* and $\mu_{i,k}(\boldsymbol{\theta})$ is the spike rate of neuron *i* at time point *k* as computed from the model with parameter set $\boldsymbol{\theta}$. Maximum likelihood estimation of the model's parameters is equivalent to minimizing the weighted sum of squared errors (*WSSE*):

$$WSSE = \sum_{i,k} \left(x_{i,k} - \mu_{i,k}(\boldsymbol{\theta}) \right)^2 \Big/ \sigma_{i,k}^2 \quad . \tag{7.2}$$

To find the parameter set minimizing the *WSSE* in Eq. (7.2), we used an algorithm that combined the MATLAB (Mathworks) genetic algorithm function ga() and the gradient descent algorithm function fmincon(). Although gradient descent optimized the parameter set faster than the genetic algorithm, it sometimes caused the parameter set to be trapped in a local minimum of the *WSSE*. Therefore, we first used the genetic algorithm to find a rough estimate of the parameter set, and then we used gradient descent to fine-tune the parameters (**Supplementary Table 3**). The function fmincon() provides the matrix of second derivatives of the *WSSE* (*i.e.*, the Hessian matrix of *WSSE*, H_{WSSE}) for the optimized parameter set (Eq. (7.3)):

$$H_{WSSE} = \nabla_{\boldsymbol{\theta}}^2 \sum_{i,k} \left(x_{i,k} - \mu_{i,k}(\boldsymbol{\theta}) \right)^2 \Big/ \sigma_{i,k}^2 \Bigg|_{\boldsymbol{\theta}=\hat{\boldsymbol{\theta}}} \quad , \tag{7.3}$$

where $\hat{\boldsymbol{\theta}}$ is the set of optimized parameter values, and the symbol $\nabla_{\boldsymbol{\theta}}^2$ denotes second derivatives.

Based on Eqs. (7.1) and (7.3), $H_{WSSE}/2$ equals the observed Fisher information provided by the optimized parameter set about the underlying, real biological parameter values. Using the Fisher information approach, the Hessian matrix can be used to estimate the 68% confidence interval (16th–84th percentiles) for each parameter:

$$CI_{\theta_j} = \hat{\theta}_j \pm \sqrt{\left(\left(H_{WSSE}/2 \right)^{-1} \right)_{jj}} \, , \tag{7.4}$$

where $\hat{\theta}_j$ is the optimized value of the jth parameter and $\left(\left(H_{WSSE}/2\right)^{-1}\right)_{jj}$ is the element in the jth row and the jth column of the covariance matrix $\left(H_{WSSE}/2\right)^{-1}$.

Supplementary Table 3 below shows the optimized values and confidence intervals of the parameter set for the two- and three-module versions of the model. **Supplementary Fig. 1** shows that the fit parameters used in both model variants were statistically indistinguishable and unchanged by the omission of the α2 module in the simpler variant. This indicates that inclusion of the α2 compartment is not needed to account for our results about the other compartments, suggesting a minimal role for α2 in the conditioning and extinction protocols explored in this paper.

We compared the numbers of synapses between pairs of neurons, as found in the connectome data, to the synaptic weight values found via parametric fits. Linear regression analyses showed that these two sets of synaptic weights are uncorrelated, regardless of whether the model has two or three modules ($P = 0.319$ and $P = 0.572$, respectively). There are several reasons why it may be hard to infer the relative weights of synapses in the model directly from synapse number counts. One possibility is that while there may be several types of synapses that use different neurotransmitters or neuromodulators, these distinctions and their impact on functional connection strengths cannot be readily inferred from the electron microscopy data. Another possible reason is that the connectome data are from a single fly, but the synaptic weight values in the fitted models were determined from the large number (>500) of flies tested in our experiments. A third possibility is that, in our model, the synaptic weights represent functional connection strengths that summarize the cumulative impact of both direct and indirect connections between two neurons, the latter of which is not reflected in the synapse counts.

When we used our model to predict neural spike rates, we determined the spike rate values generated from the model with the maximum *a posteriori* probability by inputting the values of the optimized parameter set into our model (**Fig. 5**; **Extended Data Fig. 10**). To estimate the confidence

intervals of the predicted spike rate values, we sampled 10,000 different sets of parameters from the normal distribution with the covariance matrix $\left(H_{WSSE}/2\right)^{-1}$ and ran the model simulation with each of them. We used the 16th–84th percentiles of the distribution of the model-predicted values to determine the confidence intervals.

To evaluate whether the fitted parameter values for the two-module model differed significantly from the values of the corresponding parameters in the three-module model, we calculated the ratios of the parameter values for the two models and tested whether these ratios were significantly different from one (**Supplementary Figure 1**). To do this, we took 10,000 sets of parameters for each model and calculated the ratios of the parameter values between the two models. We used the 50th percentile value of each parameter ratio to determine its median value. We used the 16th–84th percentile range for each parameter ratio to determine its 68% confidence interval. As shown in **Supplementary Figure 1**, all of the 68% confidence intervals flank unity, implying that none of the parameter values in the two-module model differ significantly ($P > 0.32$) from the corresponding values in the three-module model.

§8. Supplementary Tables and Figure.

Supplementary Table 1 | Synapse numbers in the MB circuit of the fly connectome.

Pre-synaptic neuron	Connectome ID#*	Post-synaptic neuron	Connectome ID#*	Number of synapses
		PPL1-γ1pedc	393766777	71
		PPL1-α'2α2	5813019513	12
		PPL1-α3	331662710	25
MBON-γ1pedc>α/β	424767514	MBON-γ1pedc>α/β	424767514	0
		MBON-α2sc	5813020828	48
		MBON-α3	300972942	40
		MBON-α3	5813068729	51
		PPL1-γ1pedc	393766777	0
		PPL1-α'2α2	5813019513	12
		PPL1-α3	331662710	17
MBON-α2sc	5813020828	MBON-γ1pedc>α/β	424767514	1
		MBON-α2sc	5813020828	0
		MBON-α3	300972942	0
		MBON-α3	5813068729	0
		PPL1-γ1pedc	393766777	0
		PPL1-α'2α2	5813019513	0
		PPL1-α3	331662710	4
MBON-α3	300972942	MBON-γ1pedc>α/β	424767514	0
		MBON-α2sc	5813020828	0
		MBON-α3	300972942	0
		MBON-α3	5813068729	41
		PPL1-γ1pedc	393766777	2
		PPL1-α'2α2	5813019513	0
		PPL1-α3	331662710	24
MBON-α3	5813068729	MBON-γ1pedc>α/β	424767514	0
		MBON-α2sc	5813020828	0
		MBON-α3	300972942	97
		MBON-α3	5813068729	0

* The Connectome ID# is the neuron ID# in the fly hemi-brain connectome database (version 1.2.1)[1,2].

Supplementary Table 2 | Empirically measured parameter values input directly into the model.

Category	Neuron(s)	Symbol	Equation	Value	Source
**Ratios of Punishment weights**	PPL1-γ1pedc / PPL1-α3	$w_{punish,1} / w_{punish,3}$	(5.18)	2.44	**Fig. 2a,d**
	PPL1-α’2α2 / PPL1-α3	$w_{punish,2} / w_{punish,3}$	(5.18)	0	
**Maximum spiking rates (s^{-1})**	MBON-γ1pedc>α/β	$M_{MBON,1}$	(4.6)	7.17E+01	**Extended Data Figs. 4,6**
	MBON-α2sc	$M_{MBON,2}$	(4.6)	1.79E+01	
	MBON-α3	$M_{MBON,3}$	(4.6)	3.12E+01	
**Baseline spiking rates (s^{-1})**	MBON-γ1pedc>α/β	$B_{MBON,1}$	(4.13)	3.52E+01	**Extended Data Figs. 4,6**
	MBON-α2sc	$B_{MBON,2}$	(4.13)	0.90E+01	
	MBON-α3	$B_{MBON,3}$	(4.13)	1.12E+01	
**Time-constants (s)**	Kenyon cells	$\tau_{KC,adapt}$	(5.2)	2.00E+01	Murmu, M. S. *et al.*, (2011) [3]

Supplementary Table 3 | Definitions and values of parameters found by model fitting.

Category	Name	Symbol	Equation	Three-module model			Two-module model		
				Value	16th percentile	84th percentile	Value	16th percentile	84th percentile
**Anti-Hebbian amplitudes**	Anti-Hebbian amplitude of KCs and MBONs	$A_{AH}(0)$	(5.18)	-7.12	-9.21	-5.50	-7.04	-8.78	-5.64
	Anti-Hebbian amplitude on PPL1-α3	$A_{AH}(\Delta t_{punish}) w_{punish,3}$	(5.18)	-2.13E+01	-2.73E+01	-1.65E+01	-2.02E+01	-2.40E+01	-1.70E+01
**Synaptic weights**	KC to PPL1-γ1pedc (Attractive odor)	$w_{KD,1,1}, w_{KD,2,1}$	(4.12), (5.18)	-2.11	-3.25	-9.79E-01	-1.94	-2.55	-1.34
	KC to PPL1-α'2α2 (Attractive odor)	$w_{KD,1,2}, w_{KD,2,2}$	(4.12), (5.18)	-3.75	-6.10	-1.41			
	KC to PPL1-α3 (Attractive odor)	$w_{KD,1,3}, w_{KD,2,3}$	(4.12), (5.18)	4.68	3.77	5.59	3.97	2.43	5.52
	KC to PPL1-γ1pedc (Repulsive odor)	$w_{KD,3,1}, w_{KD,4,1}$	(4.12), (5.18)	5.14	3.97	6.31	5.02	4.02	6.02
	KC to PPL1-α'2α2 (Repulsive odor)	$w_{KD,3,2}, w_{KD,4,2}$	(4.12), (5.18)	2.94	1.37	4.51			
	KC to PPL1-α3 (Repulsive odor)	$w_{KD,3,3}, w_{KD,4,3}$	(4.12), (5.18)	1.37E+01	1.22E+01	1.52E+01	1.26E+01	1.08E+01	1.43E+01
	Initial value of KC to MBON-γ1pedc>α/β	$w_{KM,initial,1}$	(5.10)	2.54E+01	2.36E+01	2.73E+01	2.48E+01	2.30E+01	2.67E+01
	Initial value of KC to MBON-α'2α2	$w_{KM,initial,2}$	(5.10)	1.73E+01	1.48E+01	1.99E+01			
	Initial value of KC to MBON-α3	$w_{KM,initial,3}$	(5.10)	1.63E+01	1.50E+01	1.77E+01	1.66E+01	1.53E+01	1.78E+01
	MBON-γ1pedc>α/β to PPL1-γ1pedc	$w_{MD,1,1}$	(4.12), (5.18)	-3.83E-02	-1.16E-01	-1.27E-02	-3.68E-02	-7.29E-02	-1.86E-02
	MBON-γ1pedc>α/β to PPL1-α'2α2	$w_{MD,1,2}$	(4.12), (5.18)	-7.48E-02	-1.35E-01	-4.15E-02			
	MBON-γ1pedc>α/β to PPL1-α3	$w_{MD,1,3}$	(4.12), (5.18)	-3.82E-01	-4.41E-01	-3.30E-01	-3.50E-01	-4.22E-01	-2.90E-01
	MBON-γ1pedc>α/β to MBON-α2sc	$w_{MM,1,2}$	(4.13)	-3.09E-01	-3.99E-01	-2.39E-01			
	MBON-γ1pedc>α/β to MBON-α3	$w_{MM,1,3}$	(4.13)	-2.09E-09	-8.61E-09	-5.07E-10	-2.07E-09	-8.62E-09	-4.99E-10
	MBON-α2sc to PPL1-α'2α2	$w_{MD,2,2}$	(4.12), (5.18)	1.55E-01	8.21E-02	2.92E-01			
	MBON-α2sc to PPL1-α3	$w_{MD,2,3}$	(4.12), (5.18)	2.09E-09	5.04E-10	8.66E-09			
	MBON-α3 to PPL1-α3	$w_{MD,3,3}$	(4.12), (5.18)	2.09E-09	5.04E-10	8.67E-09	2.07E-09	4.95E-10	8.69E-09
**Time-constants (s)**	τMBON-STM (KC to MBON-γ1pedc>α/β)	$\tau_{u,1}$	(5.18)	2.02E+03	1.73E+03	2.37E+03	1.87E+03	1.64E+03	2.14E+03
	τMBON-STM (KC to MBON-α'2α2 and KC to MBON-α3)	$\tau_{u,2}, \tau_{u,3}$ ($t \le 3$ hrs)	(5.18)	6.22E+03	5.53E+03	7.00E+03	6.30E+03	5.70E+03	6.96E+03
	τMBON-LTM (KC to MBON-α'2α2 and KC to MBON-α3)	$\tau_{u,2}, \tau_{u,3}$ ($t > 3$ hrs)	(5.18)	2.43E+05	1.22E+05	4.82E+05	2.61E+05	1.30E+05	5.21E+05
	τKC-recover	$\tau_{KC,recover}$	(5.2), (5.3)	7.92E+02	6.75E+02	9.29E+02	7.12E+02	5.85E+02	8.68E+02

Supplementary Figure 1 | Comparisons of parameter values in the 2- and 3-module model variants.

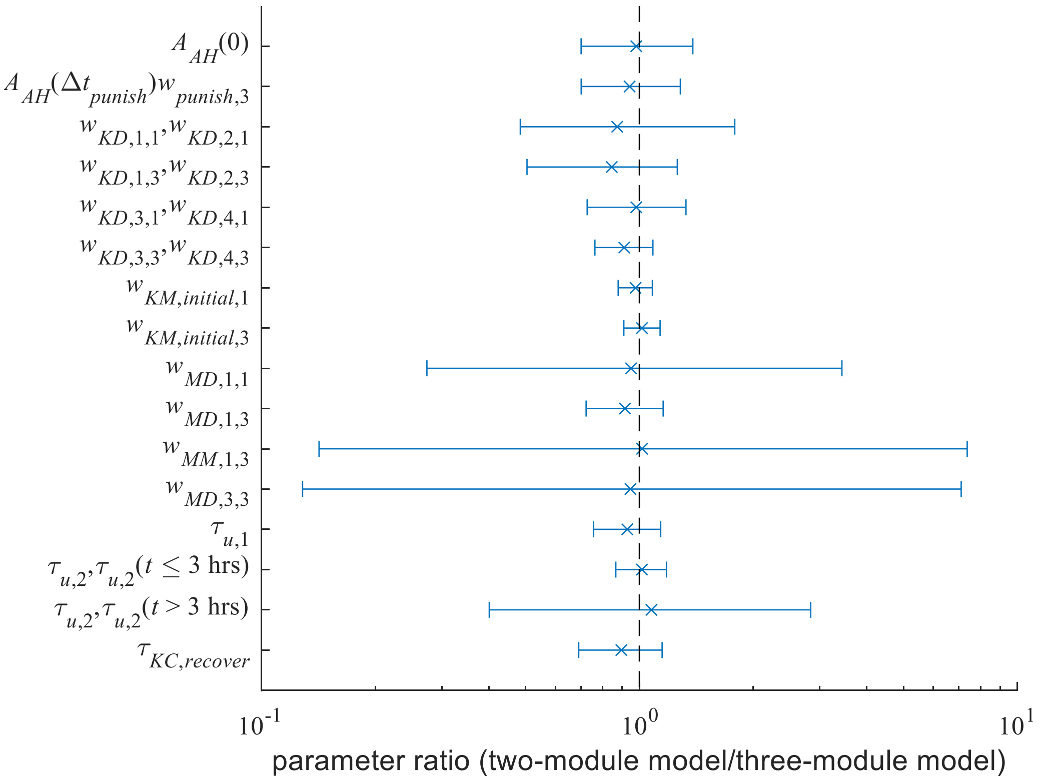

To compare parameter values in the two-module model with the corresponding values in the three-module model, we plotted the ratios of the parameter values obtained by fitting the two different models to the experimental spike rate data. We then tested whether each ratio differed significantly from unity. In this plot, the data points (× symbols) mark the median values of the parameter ratios, and the error bars denote the 68% confidence intervals, based on 10,000 sets of parameters for each model. All of these confidence intervals flank unity (vertical dashed line). Thus, all fitted parameter values in the two-module model are statistically indistinguishable ($P > 0.32$; Monte Carlo testing) from the corresponding parameter values in the three-module model.

§9. References.

1. Scheffer, L. K. *et al.* A connectome and analysis of the adult Drosophila central brain. *Elife* **9**, (2020).
2. Li, F. *et al.* The connectome of the adult Drosophila mushroom body provides insights into function. *Elife* **9**, (2020).
3. Murmu, M. S., Stinnakre, J., Réal, E. & Martin, J.-R. Calcium-stores mediate adaptation in axon terminals of olfactory receptor neurons in Drosophila. *BMC Neurosci.* **12**, 105 (2011).
4. Gerber, B. *et al.* Pain-relief learning in flies, rats, and man: basic research and applied perspectives. *Learn. Mem.* **21**, 232–252 (2014).
5. Aso, Y. & Rubin, G. M. Dopaminergic neurons write and update memories with cell-type-specific rules. *Elife* **5**, (2016).
6. Tanaka, N. K., Tanimoto, H. & Ito, K. Neuronal assemblies of the Drosophila mushroom body. *J. Comp. Neurol.* **508**, 711–755 (2008).
7. Séjourné, J. *et al.* Mushroom body efferent neurons responsible for aversive olfactory memory retrieval in Drosophila. *Nat. Neurosci.* **14**, 903–910 (2011).
8. Chiang, A.-S. *et al.* Three-dimensional reconstruction of brain-wide wiring networks in Drosophila at single-cell resolution. *Curr. Biol.* **21**, 1–11 (2011).
9. Takemura, S.-Y. *et al.* A connectome of a learning and memory center in the adult Drosophila brain. *Elife* **6**, e26975 (2017).
10. Wang, G. *et al.* The reconstruction and functional mapping of a recurrent microcircuit in Drosophila mushroom body. *bioRxiv* (2019) doi:10.1101/819227.
11. Amin, H., Apostolopoulou, A. A., Suárez-Grimalt, R., Vrontou, E. & Lin, A. C. Localized inhibition in the Drosophila mushroom body. *Elife* **9**, (2020).

12. Eschbach, C. *et al.* Recurrent architecture for adaptive regulation of learning in the insect brain. *Nat. Neurosci.* **23**, 544–555 (2020).

13. Aso, Y. *et al.* Mushroom body output neurons encode valence and guide memory-based action selection in Drosophila. *Elife* **3**, e04580 (2014).

14. Siegenthaler, D., Escribano, B., Bräuler, V. & Pielage, J. Selective suppression and recall of long-term memories in Drosophila. *PLoS Biol.* **17**, e3000400 (2019).

15. Candia, C., Jara-Figueroa, C., Rodriguez-Sickert, C., Barabási, A.-L. & Hidalgo, C. A. The universal decay of collective memory and attention. *Nat. Hum. Behav.* **3**, 82–91 (2019).

16. Igarashi, N., Okada, Y., Sayama, H. & Sano, Y. A two-phase model of collective memory decay with a dynamical switching point. *Sci. Rep.* **12**, 21484 (2022).

17. Margulies, C., Tully, T. & Dubnau, J. Deconstructing memory in Drosophila. *Curr. Biol.* **15**, R700-13 (2005).
